# Transition-Metal-Free One-Pot Synthesis of (Hetero)chalcones with Cysteine Protease Inhibitory Activity

**DOI:** 10.1021/acsomega.5c08888

**Published:** 2026-01-29

**Authors:** Thais Rodrigues Arroio, Franco Jazon Caires, Gabriela de Oliveira Almeida, Victor Hugo Catricala Fernandes, Isabela Wada Ferreira Pinto, Luíz Vinícius Santos de Oliveira, Paulo Cezar Vieira, Giuliano Cesar Clososki

**Affiliations:** Department of Biomolecular Sciences, School of Pharmaceutical Sciences of Ribeirão Preto, University of São Paulo, Av. do Café s/n, Ribeirão Preto, Sao Paulo 14040-903, Brazil

## Abstract

We report a new and efficient one-pot methodology for the synthesis of (hetero)­chalcones via direct C–H functionalization. The protocol employs directed organolithiation of aromatic and heteroaromatic substrates, followed by in situ formylation using inexpensive DMF, which not only serves as the formylating agent but also generates lithium dimethylamide as the base for the subsequent aldol condensation with (hetero)­aryl ketones. This transition-metal-free and additive-free approach enables the preparation of 23 chalcone derivatives with broad structural diversity, varying both aromatic and heteroaromatic units, in isolated yields up to 85%. To demonstrate the synthetic utility of the obtained chalcones, a model chalcone was further transformed into two different derivatives, including a pyrazole and a thioacetic acid derivative. The synthesized chalcones were evaluated through enzymatic inhibition assays against cysteine proteases papain and Cathepsin B (CatB), with compound **3c** (bearing a *para*-chloro substituent) showing the highest potency (IC_5__0_ = 7.54 ± 0.99 μM) against papain. These results were supported by molecular docking studies, which highlighted key interactions between (hetero)­chalcones, especially compound **3c**, and catalytic residues, reinforcing its potential as a fragment-like starting point for drug design.

## Graphical abstract

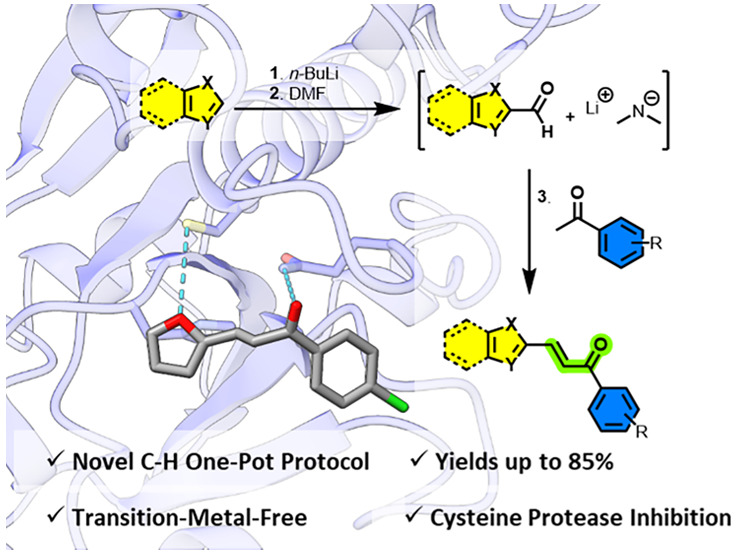

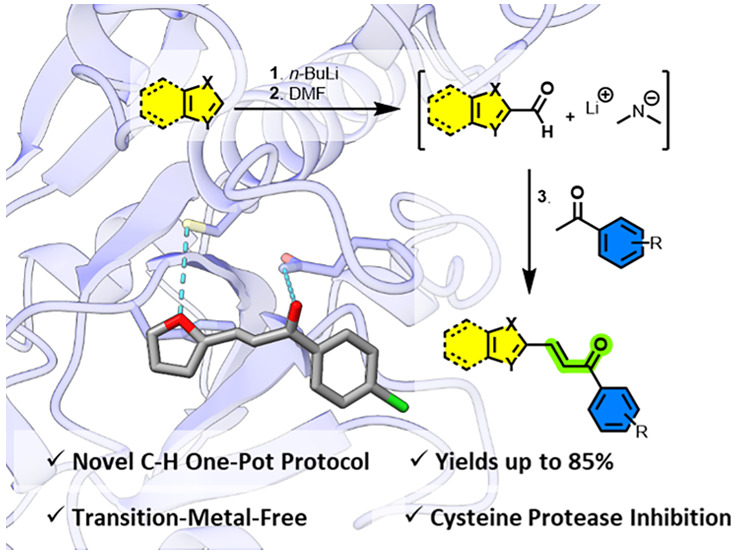

## Introduction

As a significant group of organic compounds, chalcones have been extensively studied in various fields of scientific research.^1^ This class of molecules represents numerous natural products, and the presence of a 1,3-diaryl-2-propen-1-one core characterizes their structure.^2–4^ Such conjugated α,β-unsaturated systems make them act as Michael acceptors toward thiol-containing biomolecules.^5^ In medicinal chemistry, the chalcone motif has a wide range of biological properties it may exhibit, as antifungal,^6^ antimicrobial, and antibacterial activities,^7,8^ anti-inflammatory,^9^ anticancer,^10,11^ antiviral,^12^ α-amylase and α-glucosidase inhibition,^13^ antitubercular,^14^ antimalarial,^15^ antidepressant,^16^ anticonvulsant,^17^ antidiabetic^18^ and antioxidant.^19^ Some notable examples include Metochalcone, approved for clinical use as a choleretic and diuretic agent, and Sofalcone, an oral medication for antiulcer and gastroprotective effects, as shown in Figure 1.^20^

**Figure 1:**
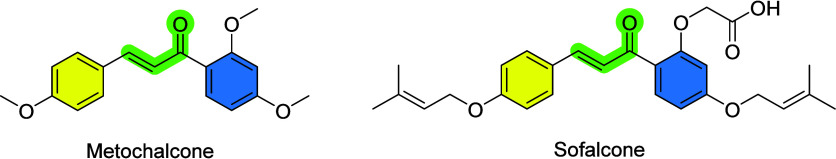
Approved drugs containing a chalcone moiety.

All the medicinal applications of chalcones have attracted increasing attention due to their broad biotechnological and pharmacological potential.^20^ Due to their relatively small molecular size, straightforward and efficient synthetic accessibility, and the possibility of fine-tuning lipophilicity (logP) through suitable substituents, chalcone derivatives have emerged as highly attractive scaffolds in drug discovery research.^21^ In this context, in vitro studies and molecular modeling have shown that certain chalcones are capable of inhibiting cysteine proteases (CPs).^22,23^ Their activity to target CPs results from the nucleophilic thiol group in the catalytic cysteine, which can interact covalently or reversibly with electrophilic motifs, highlighting their promise as a starting point for fragment-based drug design (FBDD).^24,25^

These proteases are involved in key physiological processes such as extracellular matrix remodeling, protein degradation, immune system regulation, and programmed cell death.^26^ However, CPs also play crucial roles in the pathogenesis of several disorders, including inflammatory, autoimmune, neurodegenerative, and bone diseases.^27^ Beyond human pathophysiology, CPs are vital for the life cycle and virulence of pathogens like *Trypanosoma cruzi*, *Plasmodium* species, and viruses, including SARS-CoV, facilitating host invasion, replication, and immune evasion.^28,29^

The importance of chalcones is reflected in their versatile synthesis, traditionally achieved through aldol condensation, such as the Claisen–Schmidt reaction,^30^ Wittig reaction,^31^ Julia–Kocienski olefination,^32^ Meyer–Schuster,^33^ and cross-coupling reactions.^34^ An interesting alternative for the preparation of chalcones involves the use of C–H functionalization, a powerful technique that has gained significant attention over the past few decades.^35^

In this context, employing a palladium-mediated oxidative coupling, Miki and co-workers reported a palladium-mediated protocol employing copper acetate as a cocatalyst and requiring specific anilide substrates, which limits its general applicability.^36^ Some years later, Wu and collaborators developed a scalable method using rhodium and silver catalysts, demonstrating broad functional group tolerance under mild conditions; however, this approach involves higher costs and metal waste (Scheme 1).^37^ Although these reported methodologies generally provide high synthetic viability, current methods for (hetero)­chalcone synthesis often rely on expensive precursors and transition-metal catalysts.

**Scheme 1:**
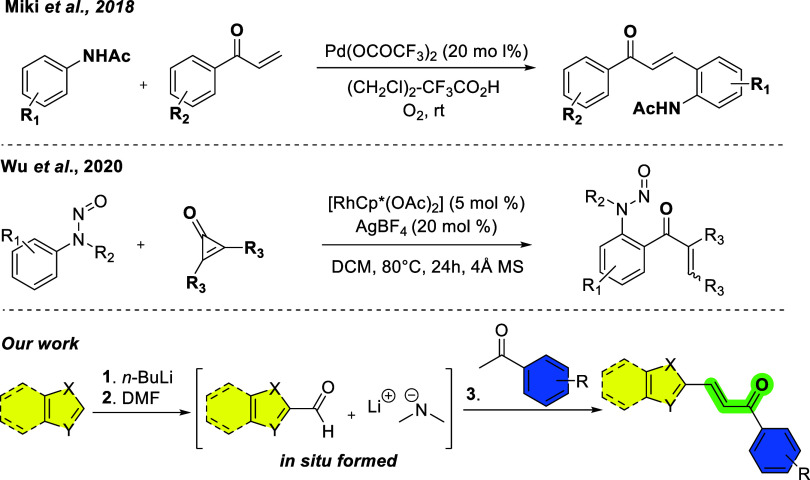
C–H Functionalization Approaches for the Synthesis of Chalcones

Herein, we developed a novel one-pot, transition-metal-free protocol for the preparation of (hetero)­chalcones that requires no prefunctionalized or costly heteroaldehyde precursors, offering a rapid and versatile platform to access this scaffold (Scheme 1). Owing to their favorable size for fragment-based drug discovery, the synthesized compounds were further evaluated for their inhibitory activity against cysteine proteases, aiming to explore their potential as protease inhibitors.

## Results and Discussion

### Synthesis

The study was initiated by selecting furan as a model aromatic substrate (ring **A**) for the directed metalation/C–H functionalization step, owing to its well-documented reactivity and ability to undergo regioselective deprotonation.^38^ Under an inert atmosphere, furan (1.0 equiv) was treated with *n*-BuLi (1.05 equiv) in THF at 0 °C for 1 h to generate the corresponding furan-lithium intermediate. The reaction mixture was then warmed to room temperature, and DMF (1.1 equiv) was added dropwise; formylation was allowed to proceed over 4 h, affording the furan-2-carboxaldehyde in situ. Finally, acetophenone (ring **B**, 1.0 equiv) was introduced at 0–5 °C, and the mixture was stirred for 16 h to effect aldol condensation and dehydration, delivering chalcone **3a**.

To select an optimal formylating reagent, we compared DMF with *N*-methyl-*N*-phenylformamide (N-MFd), 4-fluorobenzaldehyde dimethyl acetal (4-FM), and ethyl formate (EF) under otherwise identical conditions (Table 1, entries 1–4). Both DMF and N-MFd gave the desired chalcone in 67% isolated yield; 4-FM afforded 62%, while ethyl formate failed to produce detectable product. The superior yield and commercial availability of DMF led us to select it as the standard formylating agent. With the formylation protocol established, we evaluated a range of organometallic bases for the key metalation step (Table 1, entries 5–9). Substitution of *n*-BuLi by LDA or LiTMP under analogous conditions resulted in substantially diminished yields (<15%) or no reaction, likely reflecting insufficient deprotonation at the less-activated C–H site. Similarly, magnesiation attempts using TMPMgCl·LiCl and TMP_2_MgCl·2 LiCl at room temperature furnished the chalcone in only 11–29% yield, indicating partial reactivity but poor overall conversion. The turbo-Grignard reagent *i*-PrMgCl·LiCl was completely ineffective in generating the necessary aryl or heteroaryl metal species. These results underscored the unique efficacy of *n*-BuLi for directed metalation in this one-pot sequence. Consequently, the original conditions employing *n*-BuLi, DMF, and acetophenone were adopted as the operationally simplest and highest-yielding protocol for the synthesis of the desired (hetero)­chalcones.

**Table 1:**
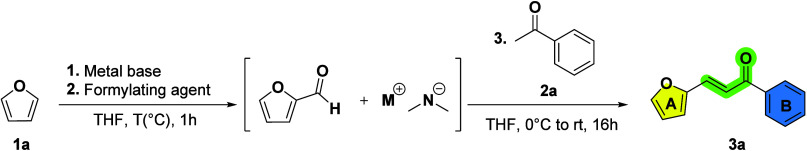
Screening of Reaction Conditions for Chalcone Synthesis

**entry**	metal base (equiv)^a^	***T*** (°C)	**formylating agent** ^b^	**yield** (isolated, %)
1	*n*-BuLi (1.05)	0	DMF	67
2	*n*-BuLi (1.05)	0	N-MFd	67
3	*n*-BuLi (1.05)	0	4-MF	62
4	*n*-BuLi (1.05)	0	EF	n.d.^c^
5	LDA (1.05)	–78	DMF	20
6	LiTMP (1.05)	–78	DMF	28
7	*i*-PrMgCl·LiCl (1.05)	0	DMF	n.d.
8	TMPMgCl·LiCl (1.3)	25	DMF	29
9	TMP_2_MgCl·2LiCl (0.65)	25	DMF	11

a LDA, LiTMP, TMPMgCl·LiCl, and TMP_2_Mg·2LiCl were freshly prepared and titrated when used, as described in the Supporting Information.

b DMF = dimethylformamide, N-MFd = *N*-methyl-*N*-phenylformamide, 4-MF = 4-formylmorpholine and EF = ethyl formate.

c n.d. = no product detected.

To evaluate the generality of our one-pot protocol concerning ring B, a series of (hetero)­aryl ketones bearing both electron-donating and electron-withdrawing substituents (as well as heteroatomic motifs) were subjected to the standard reaction conditions, while retaining furan as ring A (Scheme 2). Interestingly, the results reveal a clear electronic trend, where electron-rich aryl ketones (**3g–3l**) delivered the corresponding chalcones in markedly higher yields (40–81%), reflecting accelerated aldol condensation and dehydration under our mild conditions. In contrast, *para*-substituted EWG substrates (**3b–3f**) provided modest yields (25–48%), whereas the *meta*-substituted EWG groups (**3m**, **3n**, **3o**, **3q**) were less productive (10–31%). Nitro-substituents illustrate a strong positional effect: *o*-NO_2_ (71%) > *p*-NO_2_ (35%) > *m*-NO_2_ (10%), plausibly due to chelation-assisted organization of the reactive pair that offsets EWG-induced enolate deactivation.

**Scheme 2:**
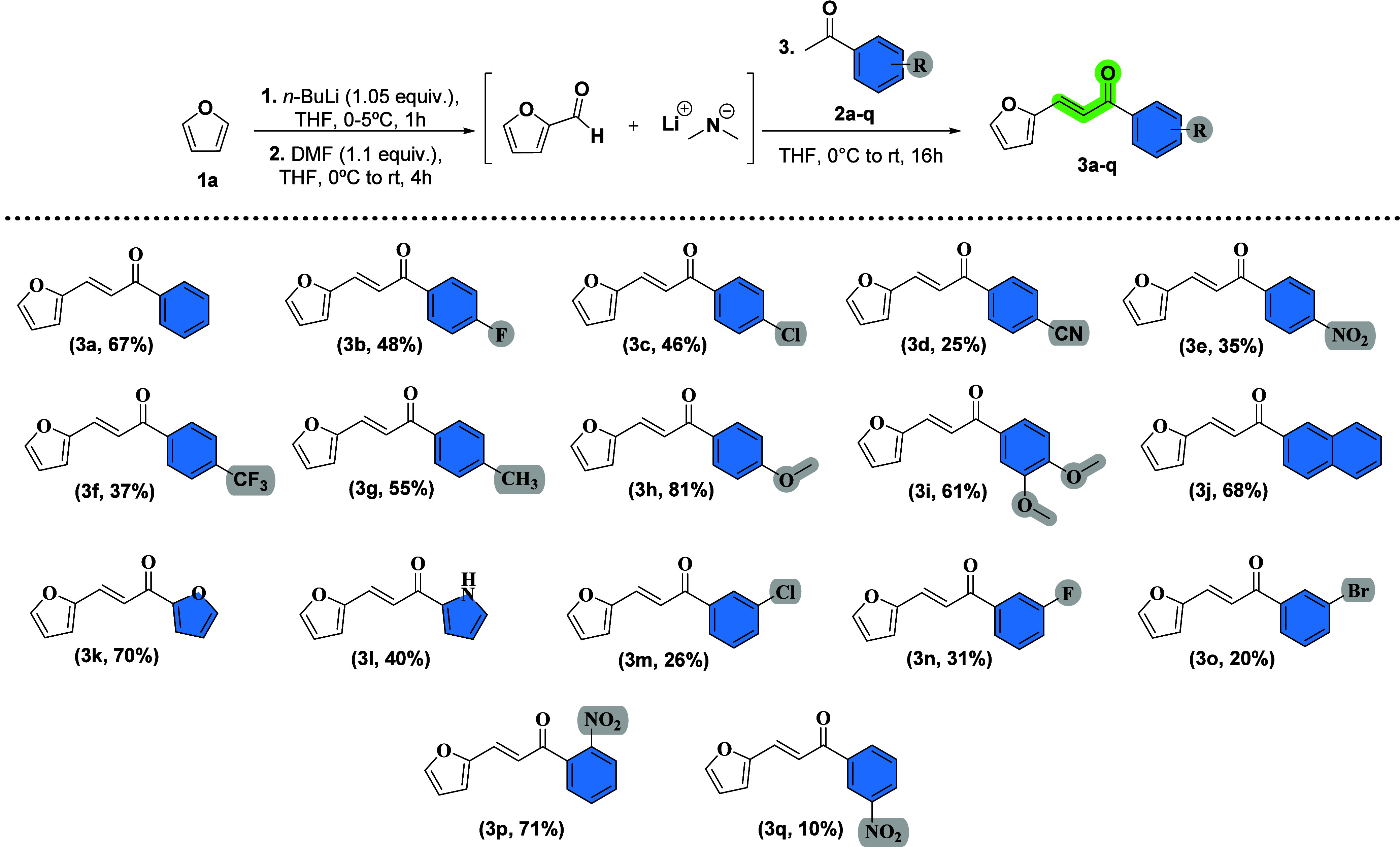
Scope for the Modification of the Chalcone B Ring

After establishing the scope with different (hetero)­aryl ketones for ring B, we next investigated the versatility of our one-pot protocol for modifying ring A by employing a range of heteroaromatic substrates (Scheme 3). Each heteroarene was subjected to directed metalation under an atmosphere of dry nitrogen (N_2_) using *n*-BuLi in THF, however, since heterocycles can display diverse acidity and coordination behavior, the metalation temperature and time were individually selected and optimized based on previously established conditions, available in the literature.^38–41^ Acetophenone was the chosen ketone for the chalcone formation, and each heteroarene derivative was submitted to the same conditions (a solution of 1.0 equiv of acetophenone in THF at 0 °C) after the formylation step. The derivative **5a** was obtained by the lithiation of thiophene using *n*-BuLi (1.05 equiv) in THF at 0 °C for 1 h. Upon addition of DMF (1 equiv), the formylation step for this example proceeded for 4 h, with an isolated yield of 76%. For benzothiophene, the lithiation employed *n*-BuLi (1.1 equiv) at −78 °C for 1 h. Subsequent addition of a solution of DMF (2 equiv in 1 mL of THF) at the same temperature for 3 h afforded the aldehyde in situ. After the ketone addition, the yield for product **5b** was 31%. Benzofuran required similar conditions for the lithiation (1.2 equiv of *n*-BuLi in THF, at −78 °C, for 1 h) and the formylation step (2 equiv of DMF in 1 mL of THF), except that the required time was 4.5 h. After ketone addition, **5c** was obtained with 52%. It is possible to observe that for the products **5b** and **5c** the same trend tends to occur, where the addition of the benzene ring caused a diminution of the yield, due to the delocalization of the aromaticity, deactivating the heteroarene portion where the lithiation occurs. The nitrogenated derivative **5d** required *n*-BuLi (1.0 equiv) at −78 °C, for five minutes to achieve the lithiation of 1-methyl-1*H*-imidazole. The subsequent formylation (2 equiv in THF) was kept at the same temperature for 1 h and an additional 40 min at 25 °C. The standard ketone addition procedure was employed, affording the product in 85%, after isolation. The high acidity of the hydrogen at the C-2 results in a significant lithiation, driving the reaction toward the chalcone formation, resulting in the yield observed for this example.

**Scheme 3:**
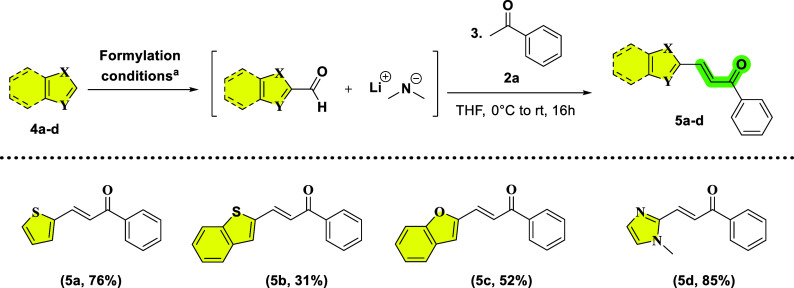
Scope of Heteroarenes for the Modification of Ring A

a The reaction conditions for the formylation of each substrate is based on literature procedures and are referenced as follows: **5a** (1.05 equiv *n*-BuLi, 0 °C, 1h; 1.0 equiv DMF, THF, 0 °C to rt, 4 h),^38^ **5b** (1.1 equiv *n*-BuLi, THF, −78 °C, 1 h; 2.0 equiv DMF, THF, −78 °C, 3 h),^39^ **5c** (1.2 equiv *n*-BuLi, THF, −78 °C, 1 h; 2.0 equiv DMF, THF, −78 °C, 4.5 h),^40^ **5d** (1.0 equiv *n*-BuLi, THF, −78 °C, 5 min; 2.0 equiv DMF, THF, −78 °C, 1 h and rt, 40 min).^41^

The formation of the chalcone product can be rationalized by the in situ generation of a lithium dimethylamide intermediate (**IV**) during the DMF-mediated formylation.^42,43^ Upon addition of acetophenone (**2a**), **IV** serves as a Bro̷nsted base to deprotonate the α-position of the ketone, forming lithium enolate **VI**. Nucleophilic addition of **VI** to the aldehyde (**V**) then gives β-hydroxyketone **VII**, which undergoes rapid dehydration under the basic reaction conditions to furnish chalcone **3a**. The directed metalation followed by formylation and intramolecular aldol condensation proceeds seamlessly in a single vessel (Scheme 4), obviating the need for external catalysts, ligands, or additives.

**Scheme 4:**
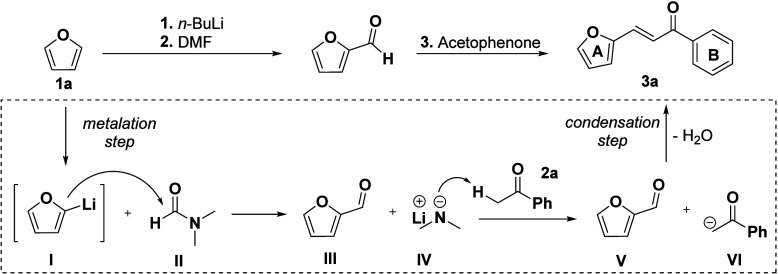
Representation of Our Hypothesis for the Mechanistic Steps Involved in the Formation of the Chalcone

To demonstrate the applicability of the developed methodology, we applied two distinct synthetic transformations to chalcone (**3a**), affording a pyrazole derivative (**6a**) and a previously unreported thioacetic acid derivative (**6b**) in 31 and 42% yields, respectively.^44–46^ These examples highlight the synthetic versatility of chalcones as valuable intermediates for accessing structurally diverse and functionally rich compounds, such as pyrazoles and sulfur-based amino acid analogues, both recognized as privileged scaffolds in medicinal chemistry (Scheme 5).

**Scheme 5:**
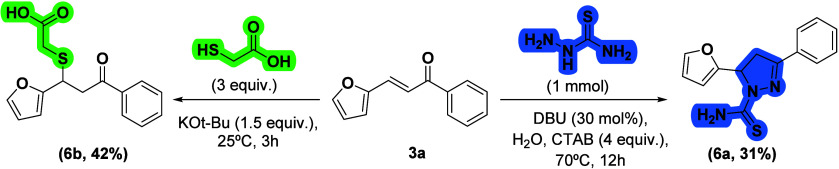
. Synthetic Applications Illustrating the Diversification of Chalcone **3a**

### Enzymatic Inhibition Activity

All synthesized chalcone derivatives were evaluated for their enzymatic inhibitory activity against the cysteine proteases papain and CatB, both classified as members of clan CA, subfamily C1A, according to the MEROPS database. These enzymes share conserved active site residues, including cysteine (Cys), histidine (His), asparagine (Asn), and glutamine (Gln).^47^ Initially, a screening assay was performed in triplicate at a fixed concentration of 50 μM to assess the inhibitory potential of each compound. A minimum inhibition threshold of 50% was established, and compounds showing average inhibition above this value at the tested concentration (disregarding error bars) were selected for IC_5__0_ determination. The enzymatic inhibition results for papain are presented in Table 2, and for CatB in Table 3. Graphs displaying the enzymatic activity of all chalcone derivatives (Figures S1 and S3) as well as the dose–response curves for IC_5__0_ determination (Figures S2 and S4)—including the positive control E-64—are provided in the Supporting Information *.*

**Table 2:**
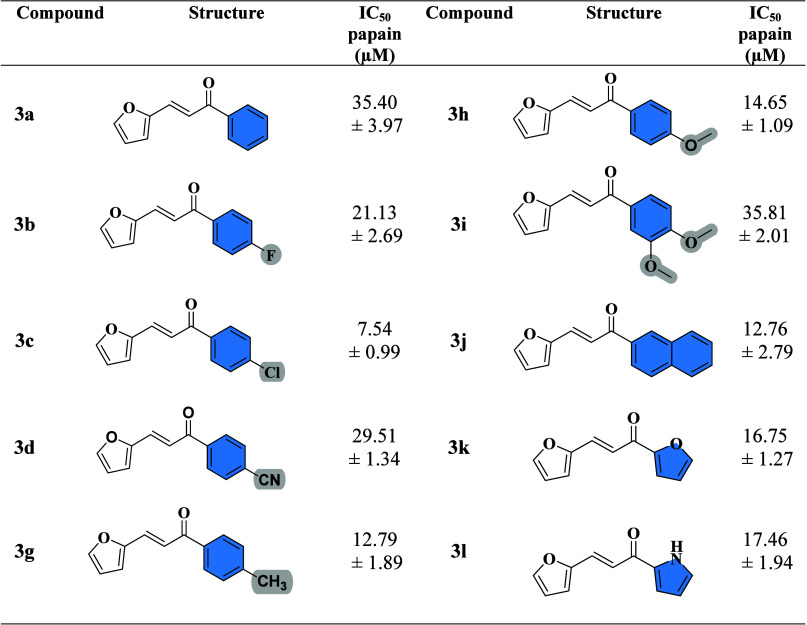
Structures of Chalcone Derivatives and Values of IC_50_ on Papain

**Table 3:**
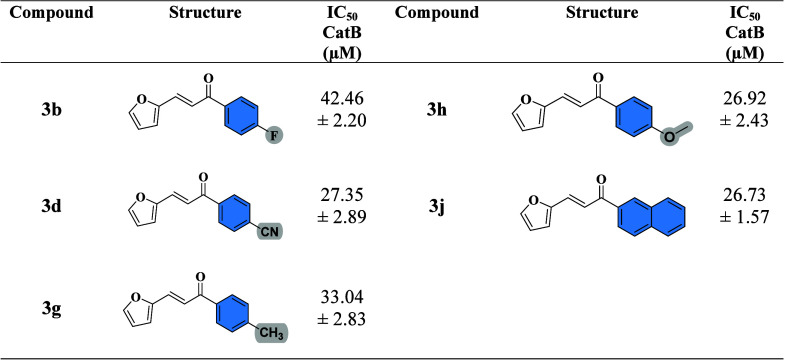
Structures of Chalcone Derivatives and Values of IC_50_ on CatB

Three main structural regions of the chalcone scaffold were targeted for modification: the aryl ring (typically a phenyl group), the heteroaromatic ring (in this case, a furan), and the central α,β-unsaturated carbonyl linker (enone system) that bridges these two moieties. Among these, the integrity of the furan ring and the enone linker proved critical for activity, as any modification within these regions consistently led to a marked decrease or loss of inhibitory potency against both papain and CatB. This suggests that these portions of the molecule are likely involved in key interactions within the active site of these papain-like cysteine proteases, possibly through hydrogen bonding, π–π stacking, or Michael acceptor-like covalent interactions with the catalytic cysteine residue.

Although the unmodified chalcone displayed only weak activity against papain (44.72 ± 4.16% at 50 μM) and CatB (27.31 ± 4.16% at 50 μM), we calculated the IC_50_ for papain due to its proximity to the 50% threshold and the role of the compound as the standard chalcone (**3a**, IC_5__0_ = 35.40 ± 3.97 μM). However, targeted substitutions on the phenyl ring significantly enhanced inhibitory activity, particularly toward papain. The most potent compound was **3c**, bearing a *para*-chloro substituent, which showed a sharp increase in activity (IC_5__0_ = 7.54 ± 0.99 μM). This result may be attributed to improved electronic or hydrophobic interactions introduced by the chlorine atom, potentially enhancing binding affinity or positioning the molecule favorably within the enzyme’s active site.

Other modifications that resulted in significant activity improvement included the introduction of a *para*-methyl substituent (**3g**, IC_5__0_ = 12.79 ± 1.89 μM), naphthalene extension (**3j**, IC_5__0_ = 12.76 ± 2.79 μM), a *para*-methoxy group (**3h**, IC_5__0_ = 14.65 ± 1.09 μM), and replacement of the phenyl ring with a furan or pyrrole ring (**3k**, IC_5__0_ = 16.75 ± 1.27 μM and **3l**, IC_5__0_ = 17.46 ± 1.94 μM). These findings indicate that both electronic and steric factors play a role in modulating enzyme binding. The addition of a *para*-fluoro group (**3b**) resulted in only a slight improvement (IC_5__0_ = 21.13 ± 2.69 μM), while other derivatives, such as **3d** (*para*-cyano, IC_5__0_ = 29.51 ± 1.34 μM) and **3i** (3,4-dimethoxy, IC_5__0_ = 35.81 ± 2.01 μM), showed little or no enhancement compared to the parent chalcone. Interestingly, while the *para*-methoxy substituent alone improved activity, adding a methoxy group at the *meta* position appeared to reduce potency. The same trend was observed for *ortho*- and *meta*- substituents on the phenyl ring (ring B), which generally resulted in loss of activity (**3m-3q,** IC_5__0_ ≥ 50 μM), possibly due to unfavorable steric hindrance or electronic interference. The positive control, E-64, displayed potent inhibition of papain with an IC_5__0_ of 7.70 ± 1.25 nM, in agreement with literature values,^48^ thus validating the assay conditions.

In contrast, for CatB, all compounds selected for IC_5__0_ determination exhibited lower potency compared to their activity against papain. While the parent chalcone showed weak inhibition, phenyl ring modifications led to modest increases in activity. Notably, several derivatives that were highly active against papain—such as **3c**, **3i**, and **3k**—demonstrated poor activity against CatB, suggesting potential issues of selectivity. The most striking example was **3c**, the most potent papain inhibitor, which displayed only 27.47 ± 7.54% inhibition at 50 μM against CatB. This divergence highlights possible differences in the active site architecture between papain and CatB, including variation in surrounding residues or differences in pocket size and shape, which could affect ligand binding. Selectivity may also be influenced by the presence of exosites or differences in surface charge distribution, which could hinder optimal orientation of certain derivatives within the catalytic groove. Once again, the strong inhibitory activity of E-64 against CatB (IC_5__0_ = 9.22 ± 0.61 nM) confirmed the reliability and reproducibility of the enzymatic assay.^49,50^

### Molecular Docking

Some selected compounds were evaluated through molecular docking studies using papain (PDB ID: 1BQI) to identify the most plausible binding pockets and to understand how structural variations influence their activity. Papain was selected as the exclusive target due to the higher inhibitory potency observed in the biological assays and its relevance as a model for the rational design of cysteine protease inhibitors, including potential applications against SARS-CoV.^29^

Most of the compounds complied with the Rule of Three, exhibiting physicochemical properties consistent with fragment-like molecules.^25^ Their structural simplicity, combined with the synthetic accessibility provided by our green and modular methodology, makes them suitable starting points for FBDD, particularly for structure-guided fragment growing strategies.^25^ Only compounds **3j** and **6a** exceeded the thresholds typically applied for fragment classification.

Two docking strategies were employed to explore the binding behavior of the compounds. The first involved targeted docking at the catalytic site, corresponding to the location of the cocrystallized inhibitor in the reference structure. The second strategy used blind docking to explore the entire protein surface, allowing the identification of alternative binding pockets, including potential allosteric sites.^51^

To further analyze the relationship between activity and binding behavior, three representative compounds were selected: **3c**, the most active (IC_50_ = 7.54 ± 0.99 μM), **3a**, with moderate activity (IC_50_ = 35.40 ± 3.97 μM), and **5d**, an inactive compound (IC_5__0_ ≥ 50 μM). All three compounds displayed consistent binding poses at the catalytic site, with superimposition observed in the same region (green, Figure 2A). Comparison of the docking scores revealed that this site allowed favorable interactions across the compounds (Figure 2B).

**Figure 2:**
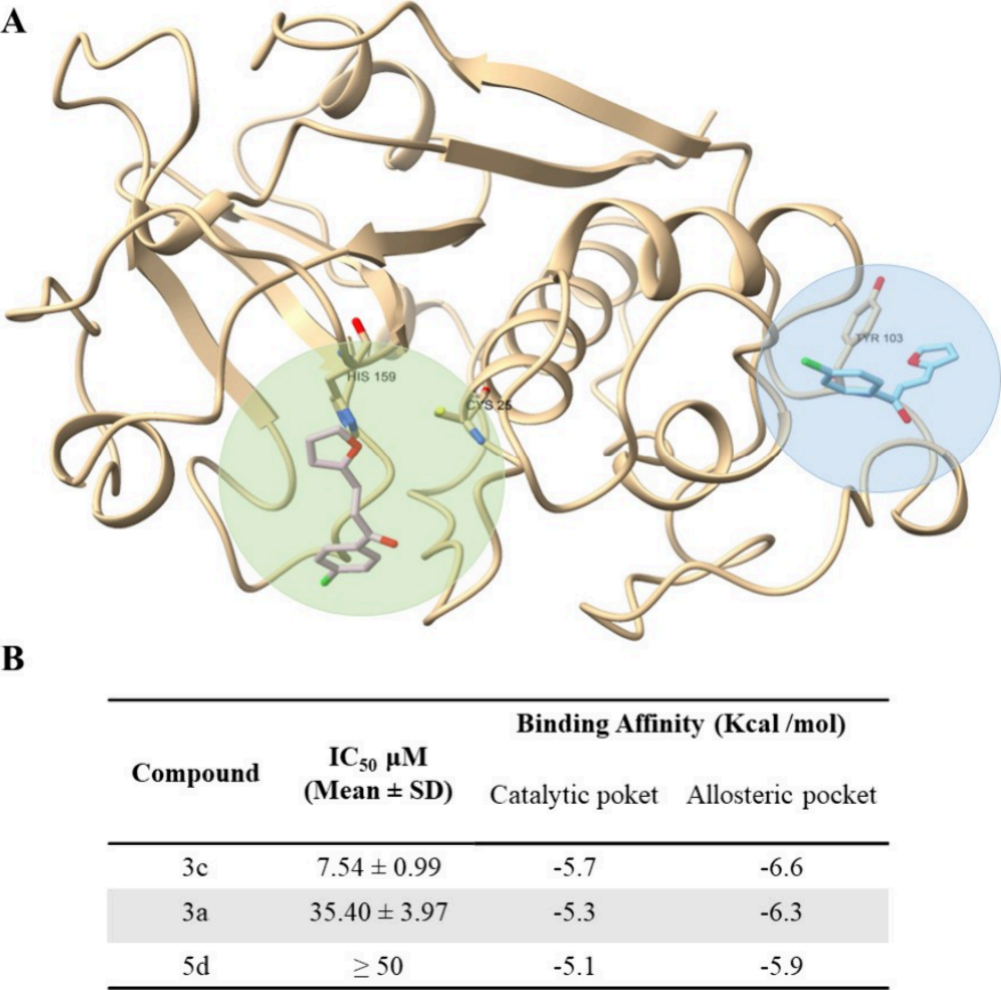
Binding pocket comparison and inhibitory activities of selected compounds docked to papain (PDB ID: 1BQI). (A) Superposition of the best binding poses of compounds **3c** from targeted and blind docking. The catalytic pocket is highlighted in green, and the possible allosteric pocket is shown in blue. (B) Table summarizing IC_5__0_ values and binding affinities (in kcal/mol) of the selected compounds for both catalytic and allosteric sites, obtained from molecular docking simulations.

Although the possible allosteric pocket (blue, Figure 2A) exhibited slightly better binding energies in some cases, the compounds did not dock in the same region as compound **3c**, which is expected in blind docking protocols. Moreover, when docking was specifically performed targeting the blue region, the predicted binding poses did not align well with the experimental enzymatic activity results. For this reason, and based on the presence of key interactions only at the catalytic site, further discussion focused primarily on the docking results obtained within the catalytic pocket.

Compound **3c** exhibited the most favorable binding pose at the catalytic site. Key interactions were observed with the essential catalytic residues Cys25, His159, and Gln19 (Figure 3A).^52^ A hydrogen bond was formed between the oxygen atom of the furan ring and Cys25, while a T-shaped π–π interaction was identified between the furan ring and the imidazole ring of His159. Additionally, the aromatic ring of the compound engaged in a π–π stacking interaction with Trp177, with an optimal angle and distance for this type of interaction. The compound was well accommodated within the hydrophobic pocket (Figure 3B), showing excellent spatial orientation and suggesting clear potential for fragment expansion through synthetic modification.

**Figure 3:**
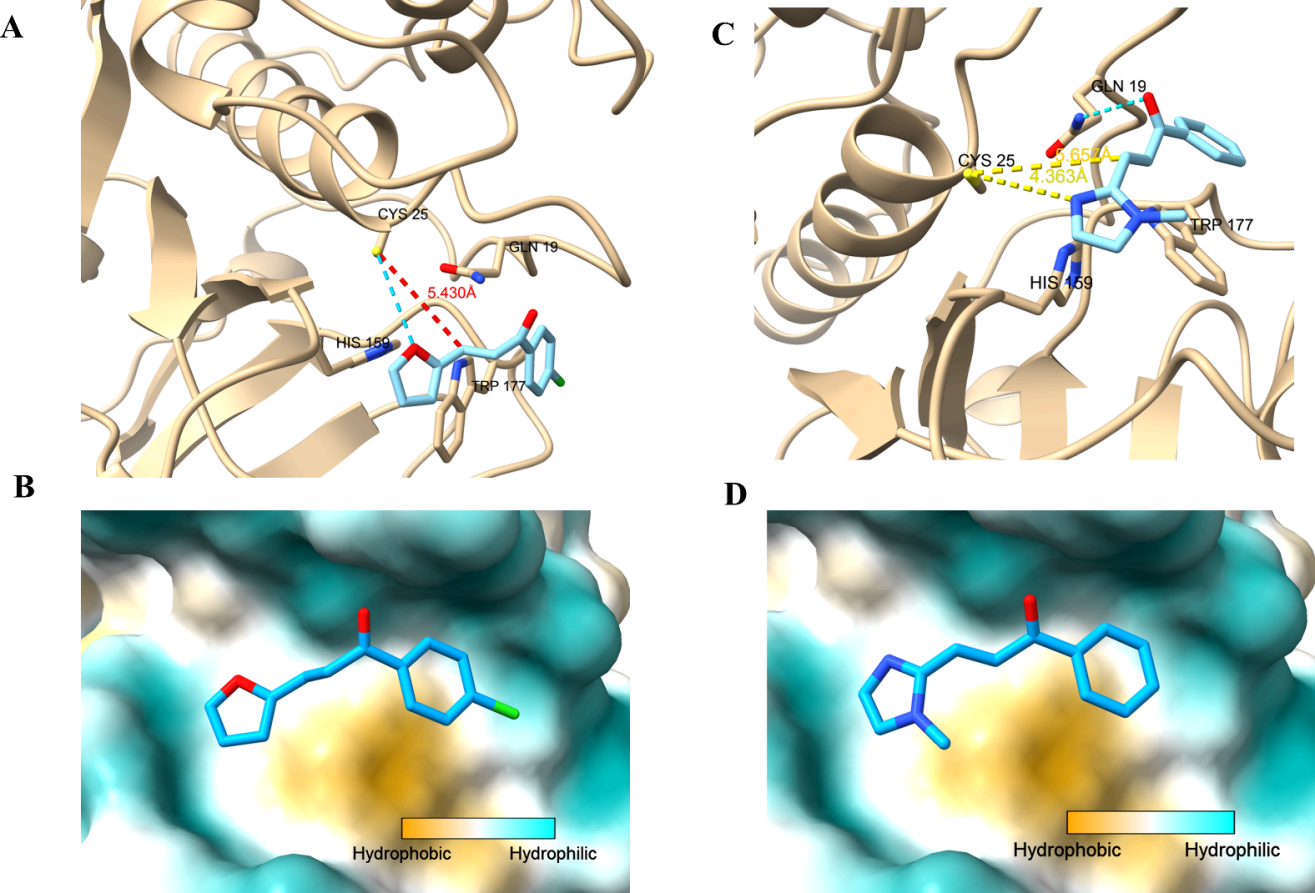
Docking analysis of compounds **3c** and **5d** at the catalytic site of papain (PDB ID: 1BQI). (A) Predicted binding pose of compound **3c**, showing key interactions with catalytic residues. Hydrogen bonds are represented in blue dashed lines, and the distance between the β-carbon of the Michael acceptor and the sulfur of Cys25 is indicated in red. (B) Molecular surface of the papain catalytic pocket colored by hydrophobicity, with compound **3c** shown in stick representation. (C) Predicted binding pose of the inactive compound **5d**, highlighting the hydrogen bond with Gln19 (blue) and the distances to Cys25 and His159 (yellow dashed lines). (D) Hydrophobic surface representation of papain with docked **5d**, illustrating its position relative to the active site.

The possibility of covalent inhibition via Michael addition to the catalytic cysteine was also evaluated. However, none of the compounds, including **3c**, positioned their α,β-unsaturated ketone moiety close enough to Cys25 to support such a mechanism. In the case of **3c**, the distance between the β-carbon and the sulfur atom of Cys25 was 5.43 Å (line in red, Figure 3A), and the angle of approach was not favorable for nucleophilic attack, indicating that covalent inhibition is unlikely and that the observed activity is due to reversible, noncovalent interactions.

In contrast, compound **5d**, which showed no significant inhibition in enzymatic assays (<50% inhibition), exhibited a different interaction profile in the docking study. Although it also occupied the catalytic site, it did not form a hydrogen bond with Cys25 and interacted only with Gln19 (Figure 3C). The molecule was positioned farther from both Cys25 and His159, preventing the formation of key hydrogen bonds or π–π interactions such as those observed for **3c**. Moreover, the imidazole ring was located in a relatively neutral lipophilic region, resulting in a limited contribution to binding affinity (Figure 3D). Nevertheless, the docking pose suggests that extending the linker region could allow better positioning of the imidazole in a more hydrophilic pocket, enabling favorable polar interactions.

The CatB assay revealed a different activity profile likely due to structural differences in the enzyme’s active site. To further explore these results, we performed molecular docking studies of CatB (PDB ID: 1CSB) with **3c**.

For compound **3c**, the docking results showed hydrogen bonds with Cys29 and Trp221, indicating that **3c** can interact with the catalytic site of CatB (Figure 4). As Cys29 is known to be the residue responsible for the enzyme’s catalytic activity, these interactions are compatible with active-site binding.^53^ However, unlike in papain, the α,β-unsaturated ketone moiety of **3c** is positioned closer to the cysteine residue in CatB, but the overall binding pose is significantly distorted. This unfavorable orientation likely prevents efficient interaction between the electrophilic center of the ligand and the nucleophilic thiol of cysteine, which could explain the weak inhibitory activity of **3c**. In addition, structural alignment of the docked conformations of compound **3c** in CatB and papain revealed a high RMSD value, indicating poor overlap between the binding poses and supporting the hypothesis of limited adaptation of **3c** to the CatB active site (Supporting Information, Figure S5).

**Figure 4:**
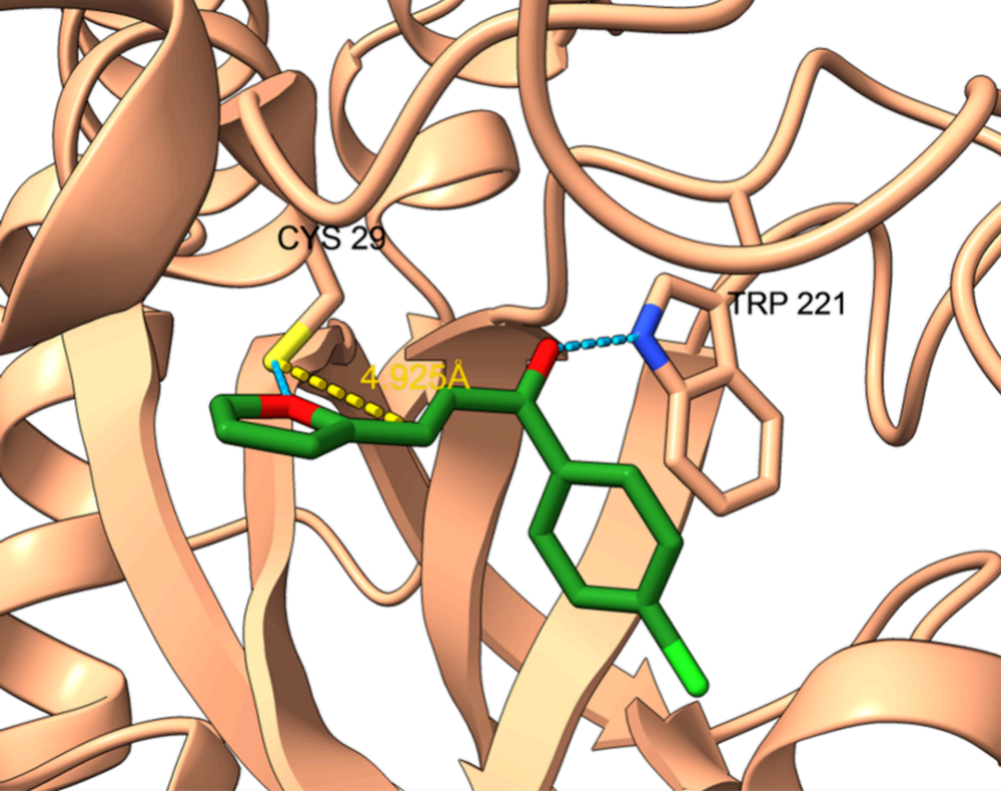
Docking analysis of compounds **3c** at the catalytic site of CatB (PDB ID: 1CSB). Binding pose of compound **3c**, showing key interactions with catalytic residues. Hydrogen bonds are represented in blue dashed lines, and the distance between the β-carbon of the Michael acceptor and the sulfur of Cys29 is indicated in yellow.

Taken together, these docking results agree with the experimental inhibition data and highlight compound **3c** as a promising fragment hit for further development. Its favorable interaction pattern and high ligand efficiency support its use as a starting point for structure-based optimization. Furthermore, the synthetic accessibility of this scaffold, combined with our environmentally friendly and efficient methodology, reinforces its potential for generating new cysteine protease inhibitors through fragment-based approaches.

## Materials and Methods

### Materials and Reagents

The starting materials, electrophiles, *n*-BuLi, *i*-PrMgCl·LiCl, and 2,2,6,6-tetramethylpiperidine were purchased from Merck, while diisopropylamine was obtained from Oakwood Chemical. All water-sensitive reactions were performed using dry solvents under anhydrous conditions and a nitrogen atmosphere. Standard syringe techniques were employed for the transfer of dry solvents and air-sensitive reagents. Progression and results were monitored by TLC on Merck silica gel (TLC silica gel 60 F254) by using UV light as a visualizing agent. Sigma-Aldrich silica gel (particle size 0.040–0.063 nm) was used for flash chromatography. Gas chromatography studies were conducted using a Shimadzu GC-2010plus chromatograph fitted with a capillary column (Restek, DB17MS-1, 30 m × 0.25 mm) and a flame ionization detector (FID). Nitrogen was used as the mobile phase. NMR spectra were recorded on Bruker DRX 300, 400, and 500 instruments (300, 400, and 500 MHz, respectively) and the chemical shifts (δ) are reported in parts per million (ppm), relative to the residual CDCl_3_ solvent peak. Mass spectra (MS) were acquired on a Shimadzu GCMS-QP 2010 equipped with DB-5 MS column and the ionization method was electron impact mode (EI, 70 eV). Helium was used as the mobile phase. Melting points were measured on a BÜCHI M-560 Type, Labortechnik AG 9230.

### Methods

#### General Procedure for the Synthesis of Furan-Based Chalcones (GP1; 3a–3q)

The chalcone derivatives were prepared using standard Schlenk techniques. In a dry nitrogen-flushed round-bottom flask under magnetic stirring, dry furan (1 mmol, 1 equiv, 0.072 mL) in anhydrous THF (4 mL, 0.25 M) was kept at 0–5 °C using an ice bath. *n*-BuLi (1.05 mmol, 1.05 equiv, 0.44 mL, 2.35 M) was added dropwise, and the reaction was allowed to stir at the same temperature for 1 h. Then, *N*,*N*-dimethylformamide (DMF) (1.1 mmol, 1.1 equiv, 0.08 mL) in THF (1 mL) was added in one portion. The ice bath was removed after 5 min, and the reaction was carried out at room temperature for 4 h. Afterward, the resulting mixture was cooled to 0–5 °C, and a solution of the corresponding acetophenone (1.0 mmol, 1.0 equiv) in THF (1 mL) was added dropwise. The reaction was continued at room temperature and monitored by TLC accordingly to the substrate. The mixture was quenched with saturated NH_4_Cl_(aq)_ until the reaction medium clarified. The resulting biphasic mixture was extracted with EtOAc (3 × 15 mL), dried over MgSO_4_, concentrated, and the crude material was purified by column chromatography on silica gel.^38^ The characterization of the purified products were conducted in GC-MS and NMR analysis, thus providing the compounds **3a–3q**, as shown in Scheme 2. The mobile phase for column chromatography and spectroscopic data for each derivative is available in the Supporting Information *.*

#### General Procedure for the Synthesis of Heterocycle-Based Chalcone Derivatives (GP2; 5a–5d)

The procedures for the formylation of substrates used in the preparation of different heterocycles were based on methodologies available in the literature and are referenced for each example. After the formation of formylated-heterocycles, the resulting mixture was cooled to 0 °C, and a solution of acetophenone (1.0 mmol, 1.0 equiv, 0.11 mL) in THF (1 mL) was added dropwise. The reaction was continued at room temperature and monitored by TLC accordingly to the substrate. The mixture was quenched with saturated NH_4_Cl­(aq) until the reaction medium clarified. The resulting biphasic mixture was extracted with EtOAc (3 × 15 mL), dried over MgSO_4_, and then concentrated. The crude material was purified by column chromatography on silica gel based on the retention factor (*R*_f_) of each product and the obtained yields for the compounds are shown in the Scheme 3, alongside with their respective literature reference.^38–41^ Characterization details are available in the Supporting Information *.*

#### Synthetic Applications for the Diversification (6a–6b)

The diversification examples of the model chalcone **3a** were synthesized following the literature procedure.^44,45^ For the compound **6a**, a mixture of the derivative **3a** (1 mmol), thiosemicarbazide (1 mmol), and DBU (30 mol %) were added to a homogeneous solution of CTAB (4 mmol) in water (10 mL). The reaction mixture was stirred at 70 °C for 12 h. Then, the reaction mixture was extracted with ethyl acetate and washed with water (3 × 20 mL). The organic phase was dried with MgSO4, filtered, and evaporated under reduced pressure. The crude material was purified by chromatography on silica gel (automatic column—Biotage, 50g) in a mixture of hexanes and EtOAc (7:3, v/v), providing a pale yellow solid in 31% yield. For the product **6b**, KO*t*-Bu (1.5 mmol) was added to **3a** (1 mmol) and thioglicolic acid (3 mmol), and the reaction mixture was stirred at room temperature for 3 h. After the reaction time, the mixture was extracted in dichloromethane, washed with diluted HCl (1 M) and water (3 × 20 mL). Then, the organic phase was dried with MgSO_4_, filtered, and evaporated under reduced pressure. The crude material was purified by chromatography on silica gel (automatic column—Biotage, 25g) in a mixture of EtOAc and hexanes (2:1, v/v), resulting in a pale yellow solid (42%) as seen in Scheme 5. The characterization data is available in the Supporting Information *.*

#### Enzymatic Assay General Procedure

The enzymatic activity of cysteine proteases was evaluated based on the hydrolysis of the fluorogenic substrate Z-Phe-Arg-4-methylcoumaryl-7-amide (Z-Phe-Arg-MCA), as described by Silva and coauthors.^54^ In the absence of an inhibitor, the protease cleaves ZFR-MCA, releasing the fluorescent product 7-amino-4-methylcoumarin (AMC), which can be monitored by Spectrofluorometry. Assays were performed in black, opaque 96-well ELISA plates by initially adding 5 μL of enzyme solution (either papain or cathepsin B) at 80 nM (final concentration 2 nM), 2 μL of dithiothreitol (DTT) at 500 mM (final concentration 5 mM), and 158 μL of sodium acetate buffer 100 mM sodium acetate buffer with 5 mM EDTA (pH 5.5) to each well. The plate was incubated at 37 °C for 10 min to ensure activation of the enzyme (reduction of the catalytic cysteine residue). Subsequently, 5 μL of each test compound (dissolved in DMSO), negative control (DMSO), or positive control (E-64 at 100 nM) was added. Compounds were initially tested at 50 μM to screen for inhibitory activity. After the addition of inhibitors, the plate was incubated again at 37 °C for 5 min to allow interaction with the enzyme active site. Next, 30 μL of ZFR-MCA substrate solution was added to initiate the reaction (final volume 200 μL per well). Final substrate concentrations were adjusted according to the reported Km values of each enzyme (90 μM for papain and 185 μM for cathepsin B).^54^ Fluorescence was monitored over 5 min using a SpectraMax M3 microplate reader (SoftMax Pro, Molecular Devices, San Jose, CA, USA) with excitation and emission wavelengths set at 380 and 460 nm, respectively. The enzymatic activity was determined by calculating the slope of fluorescence over time. Each experiment was performed in triplicate. Percentage inhibition was calculated by comparing the slope of each compound with the negative control. A threshold of 50% mean inhibition was used to select compounds for IC_5__0_ determination. IC_5__0_ values were calculated by nonlinear regression using GraphPad Prism 8.0.1 software.

#### Molecular Docking Studies

The molecular structures were designed using ChemDraw Ultra 12.0 and geometry-optimized with Avogadro 2.0. The crystal structures of papain (PDB ID: 1BQI) and Cathepsin B (PDB ID: 1CSB) were retrieved from the Protein Data Bank (https://www.rcsb.org) and prepared using AutoDock Tools. Molecular docking simulations were performed using AutoDock Vina, integrated with AMDock software. For directed docking, the grid box was centered on the catalytic cysteine residue (Cys25 for papain and Cys29 for CatB) with a grid size of 20 Å. Blind docking was also carried out for papain using the same software, employing the search space function to identify potential alternative binding cavities across the entire protein surface. The lowest binding energy conformation for each ligand was selected as the representative binding pose. The best docking results were further analyzed to characterize molecular interactions between the enzymes and ligands **3a**, **3c**, and **5d**. Finally, structural alignment of the docked complexes of compound **3c** in papain and CatB was performed using Discovery Studio to compare the conformational differences between both systems.

## Conclusions

In summary, we report a protocol for the synthesis of (hetero)­chalcones enabled by C–H functionalization in a one-pot fashion. A key feature of this method is the in situ generation of lithium dimethylamide from inexpensive DMF, which drives the aldol condensation and affords the desired (hetero)­chalcones. This transition-metal-free strategy allows the use of readily available starting materials that can be selectively converted to aldehydes using established methods. As a result, it avoids the need for costly prefunctionalized reagents and offers flexibility in designing specific chalcone derivatives. To explore their biological potential, all synthesized chalcones were evaluated for their ability to inhibit two papain-like cysteine proteases: papain and CatB. Our results revealed that modifications at the phenyl moiety with substituents in *para*-position significantly influenced the inhibitory activity, while changes at the central enone system or the furan ring generally led to loss of activity, suggesting their importance for binding to the enzyme active site. Several derivatives exhibited enhanced inhibition of papain compared to the parent chalcone, with compound **3c** (bearing a *para*-chlorophenyl substituent) showing the highest potency (IC_5__0_ = 7.54 ± 0.99 μM). In contrast, the inhibitory profiles against CatB were generally lower, suggesting potential selectivity among papain-like enzymes.

Molecular docking studies provided a structural basis for the observed activities. Compound **3c** showed a well-oriented binding pose in papain, engaging key residues (Cys25, His159, Trp177) consistent with its high potency. In contrast, **5d** lacked these interactions, explaining its inactivity. In the case of Cathepsin B, compound **3c** displayed a distinct binding orientation at the catalytic site, suggesting that its altered pose may underlie the observed differences in activity.

Taken together, these findings not only validate the biological results but also underscore the relevance of our synthetically accessible chalcone and quinoline derivatives as valuable fragment-like scaffolds for FBDD approaches targeting cysteine proteases.

## Supplementary Material



## Notes

General information, experimental procedures, enzymatic inhibition assay, molecular docking, and NMR spectra (PDF)
