# Supplementary material for: Transition-Metal-Free One-Pot Synthesis of (Hetero)chalcones with Cysteine Protease Inhibitory Activity
Source: ACS Omega. 2026 Jan 29;11(5):7616–26. doi: 10.1021/acsomega.5c08888 (PMC12903000; doi:10.1021/acsomega.5c08888)
Supplement: Supplementary file 1 [file ao5c08888_si_001.pdf]

Supporting Information

Transition-Metal-Free One-Pot Synthesis of (Hetero)Chalcones with Cysteine Protease Inhibitory Activity

Thais Rodrigues Arroio[(a)], Franco Jazon Caires[(a)], Gabriela de Oliveira Almeida[(a)], Victor Hugo Catricala Fernandes[(a)], Isabela Wada Ferreira Pinto[(a)], Luíz Vinícius Santos de Oliveira[(a)], Paulo Cezar Vieira[(a)], and Giuliano Cesar Clososki[(a)]*.

[(a)] Department of Biomolecular Sciences, School of Pharmaceutical Sciences of Ribeirão Preto, University of São Paulo, Av. do Café *s/n*, Ribeirão Preto, SP 14040-903, Brazil.

* *Email:* gclososki@usp.br

Table of Contents

1. General information 3

2. Experimental Procedures 4

2.1 General Procedure for Enzymatic Assay 4

2.2 General Procedure for Docking Studies 4

2.3 Typical Procedure for the Preparation of LiTMP (TPP 1) 5

2.4 Typical Procedure for the Preparation of TMPMgCl·LiCl (TPP 2) 5

2.5 Typical Procedure for the Preparation of $TMP_2Mg·2LiCl$ (TPP 3) 5

2.6 Typical Procedure for the Preparation of LDA (TPP 4) 6

2.7 General Procedure for the Preparation of Furan-Based Chalcones 3a-q (GP 1) 6

2.8 General Procedure for the Preparation of the Heterocycle-Based Substrates used in the scope of Chalcones 5a-d (GP 2) 15

2.9 Synthetic applications for diversification of chalcone 3a 18

3. Enzymatic Inhibition Assay 20

3.1 Enzymatic inhibition of papain 20

3.2 Enzymatic inhibition of cathepsin B (CatB) 22

4. Molecular Docking 23

5. NMR Spectra 24

6. References 47

1. General information

All solvents and reagents were purified according to standard procedures.[1] The starting materials, electrophiles, *n*-butyllithium (*n*-BuLi), *i*-PrMgCl·LiCl, and 2,2,6,6-tetramethylpiperidine were purchased from Sigma-Aldrich, while diisopropylamine was obtained from Oakwood Chemical. All water-sensitive reactions were performed using dry solvents under anhydrous conditions and a nitrogen atmosphere. Standard syringe techniques were employed for the transfer of dry solvents and air-sensitive reagents.

Chromatography

The reactions were monitored by TLC on Merck silica gel (TLC silica gel 60 F_{254}) by using UV light as a visualizing agent. Sigma-Aldrich silica gel (particle size 0.040-0.063 nm) was used for flash chromatography. Gas chromatography studies were conducted using a Shimadzu GC-2010plus chromatograph fitted with a capillary column (Restek, DB17MS - 1, 30 m × 0.25 mm) and a flame ionization detector (FID). Nitrogen was used as the mobile phase.

NMR spectra

NMR spectra were recorded on Bruker DRX 300, 400 and 500 instruments (300, 400 and 500 MHz for ^{1}H; 75, 101 and **125** for ^{13}C, respectively). Chemical shifts (δ) are reported in parts per million (ppm) relative to the residual solvent peak as the internal reference: $CDCl_3$ (δ = 7.26 ppm for ^{1}H and δ = 77.16 ppm for ^{13}C), DMSO-d_6 (δ = 2.50 ppm for ^{1}H and δ = 39.52 ppm for ^{13}C) and CD_3OD (δ = 3.31 ppm for ^{1}H and δ = 49.00 ppm for ^{13}C). Coupling constants (*J*) are given in Hz and multiplicities of the signals are abbreviated as follows: s = singlet; bs = broad singlet; d = doublet; t = triplet; m = multiplet; dd = doublet of doublets and td = triplet of doublets and app = apparent.

Mass spectra

Mass spectra (MS) were acquired on a Shimadzu GCMS-QP 2010 equipped with DB-5 MS column and the ionization method was electron impact mode (EI, 70 eV). Helium was used as the mobile phase.

Melting point

Melting points were measured on a BÜCHI M-560 Type, Labortechnik AG 9230.

2. Experimental Procedures

2.1 General Procedure for Enzymatic Assay

The enzymatic activity of cysteine proteases was evaluated based on the hydrolysis of the fluorogenic substrate Z-Phe-Arg-4-methylcoumaryl-7-amide (Z-Phe-Arg-MCA), as described by Silva et al. (2020).[2] In the absence of an inhibitor, the protease cleaves ZFR-MCA, releasing the fluorescent product 7-amino-4-methylcoumarin (AMC), which can be monitored by Spectrofluorometry. Assays were performed in black, opaque 96-well ELISA plates by initially adding 5 µL of enzyme solution (either papain or cathepsin B) at 80 nM (final concentration 2 nM), 2 µL of dithiothreitol (DTT) at 500 mM (final concentration 5 mM), and 158 µL of sodium acetate buffer 100 mM sodium acetate buffer with 5 mM EDTA (pH 5.5) to each well. The plate was incubated at 37 °C for 10 minutes to ensure activation of the enzyme (reduction of the catalytic cysteine residue). Subsequently, 5 µL of each test compound (dissolved in DMSO), negative control (DMSO), or positive control (E-64 at 100 nM) was added. Compounds were initially tested at 50 µM to screen for inhibitory activity. After the addition of inhibitors, the plate was incubated again at 37 °C for 5 minutes to allow interaction with the enzyme active site. Next, 30 µL of ZFR-MCA substrate solution was added to initiate the reaction (final volume 200 µL per well). Final substrate concentrations were adjusted according to the reported Km values of each enzyme (90 µM for papain and 185 µM for cathepsin B).[2] Fluorescence was monitored over 5 minutes using a SpectraMax M3 microplate reader (SoftMax Pro, Molecular Devices, San Jose, CA, USA) with excitation and emission wavelengths set at 380 and 460 nm, respectively. The enzymatic activity was determined by calculating the slope of fluorescence over time. Each experiment was performed in triplicate. Percentage inhibition was calculated by comparing the slope of each compound with the negative control. A threshold of 50% mean inhibition was used to select compounds for IC_{50} determination. IC_{50} values were calculated by nonlinear regression using GraphPad Prism 8.0.1.

2.2 General Procedure for Docking Studies

The molecular structures were designed using ChemDraw Ultra 12.0 and geometry-optimized with Avogadro 2.0. The crystal structures of papain (PDB ID: 1BQI) and Cathepsin B (PDB ID: 1CSB) were retrieved from the Protein Data Bank (https://www.rcsb.org) and prepared using AutoDock Tools.

Molecular docking simulations were performed using AutoDock Vina, integrated with AMDock software. For directed docking, the grid box was centered on the catalytic cysteine residue (Cys25 for papain and Cys29 for CatB) with a grid size of 20 Å. Blind docking was also carried out for papain using the same software, employing the search space function to identify potential alternative binding cavities across the entire protein surface.

The lowest binding energy conformation for each ligand was selected as the representative binding pose. The best docking results were further analyzed to characterize molecular interactions between the enzymes and ligands **3a**, **3c** and **5d**.

Finally, structural alignment of the docked complexes of compound **3c** in papain and CatB was performed using Discovery Studio to compare the conformational differences between both systems.

2.3 Typical Procedure for the Preparation of LiTMP (TPP 1)

In a dry nitrogen-flushed round-bottom flask under magnetic stirring containing 2,2,6,6-tetramethylpiperidine (0.18 mL, 1.05 mmol) in dry THF (2.0 mL), LiTMP was prepared by the slow addition of *n*-BuLi (2.35 M in hexanes, 1.0 mmol, 0.42 mL) at −70°C, using a dry ice bath. Following the addition, the resulting mixture was allowed to stir at this temperature for 10 minutes. Subsequently, the resulting pale-yellow solution was stirred for an additional 20 minutes at 0°C in an ice bath.

2.4 Typical Procedure for the Preparation of TMPMgCl·LiCl (TPP 2)

In a dry nitrogen-flushed round-bottom flask under magnetic stirring, containing *i*-PrMgCl·LiCl (1.0M in THF, 20.0 mL, 20.0 mmol), was added 2,2,6,6-tetramethylpiperidine (3.52 mL, 21.0 mmol) dropwise through a syringe over 5 min. The mixture was stirred at 20-25°C for 48 hours. Titration with benzoic acid in THF in the presence of 4-(phenylazo)diphenylamine indicated that the base concentration ranged from 0.9 to 0.98 M. Quenching with distilled benzaldehyde may be necessary to ensure the absence of turbo-Grignard species in the final mixture.

2.5 Typical Procedure for the Preparation of $TMP_2Mg·2LiCl$ (TPP 3)

In a dry nitrogen-flushed round-bottom flask under magnetic stirring containing 2,2,6,6-tetramethylpiperidine (0.9 mL, 5.25 mmol) in dry THF (10.0 mL), LiTMP was prepared by the slow addition of *n*-BuLi (2.35 M in hexanes, 5.0 mmol, 2.1 mL) at −70°C, using a dry ice bath. After addition, the resulting mixture was allowed to stir at this temperature for 10 minutes. After this time, the resulting pale-yellow solution stirred for 20 minutes at 0 °C (ice bath). In this temperature, TMPMgCl·LiCl (1 equiv., previously prepared and titrated according to the **TPP 2**) was slowly added to the reaction system and kept stirring for a further 15 minutes at room temperature before use. Titration with benzoic acid in THF in the presence of 4-(phenylazo)diphenylamine indicated quantitative preparation of $TMP_2Mg·2LiCl$ in all cases.

2.6 Typical Procedure for the Preparation of LDA (TPP 4)

In a dry nitrogen-flushed round-bottom flask under magnetic stirring containing *N,N*-diisopropylethylamine (DIPA) (0.18 mL, 1.08 mmol) in dry THF (1.6 mL), *n*-BuLi (2.35 M in hexanes, 1.0 mmol, 0.42 mL) was slowly added at −70°C, using a dry ice bath. The resulting mixture was allowed to stir at this temperature for 10 minutes. Subsequently, the temperature was increased to 0°C and maintained under stirring for an additional 20 minutes.

2.7 General Procedure for the Preparation of Furan-Based Chalcones 3a-q (GP 1)

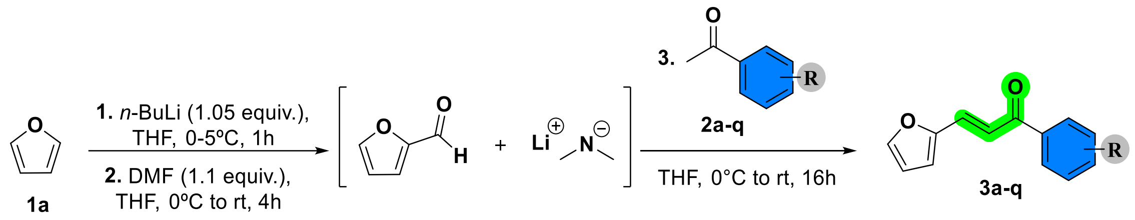

In a dry nitrogen-flushed round-bottom flask under magnetic stirring, dry furan (1 mmol, 1 equiv., 0.072 mL) in anhydrous THF (4 mL, 0.25 M) was kept at 0-5 °C using an ice bath. *n*-BuLi (1.05 mmol, 1.05 equiv., 0.44 mL, 2.35 M) was added dropwise, and the reaction was allowed to stir at the same temperature for 1 hour. Then, *N,N*-dimethylformamide (DMF) (1.1 mmol, 1.1 equiv., 0.08 mL) in THF (1 mL) was added in one portion. The ice bath was removed after 5 minutes, and the reaction was carried out at room temperature for 4 hours. Afterward, the resulting mixture was cooled to 0-5 °C, and a solution of the corresponding acetophenone (1.0 mmol, 1.0 equiv.) in THF (1 mL) was added dropwise. The reaction was continued at room temperature and monitored by TLC accordingly to the substrate. The mixture was quenched with saturated $NH_4Cl_{(aq)}$ until the reaction medium clarified. The resulting biphasic mixture was extracted with EtOAc (3 × 15 mL), dried over $MgSO_4$, concentrated, and the crude material was purified by column chromatography on silica gel.

(*E*)-3-(furan-2-yl)-1-phenylprop-2-en-1-one (3a)

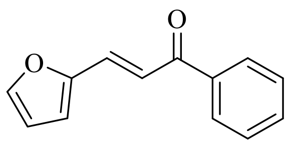

Prepared according to **GP 1** from furan (1 mmol, 1 equiv., 0.072 mL), *n*-BuLi (1.05 mmol, 1.05 equiv., 0.44 mL, 2.35 M), *N,N*-dimethylformamide (DMF) (1.1 mmol, 1.1 equiv., 0.08 mL), and acetophenone (1.0 mmol, 1.0 equiv.,0.11 mL). The crude material was purified by column chromatography on silica gel in a mixture of *Hexanes* and EtOAc (9:1, v/v). **CAS Nº:** 717-21-5. Yellow oil, 1 mmol scale (132 mg, 67%).

^{1}H NMR (400 MHz, $CDCl_3$, ppm): δ 8.01 – 7.98 (m, 2H), 7.57 (d, *J* = 15.3 Hz, 1H), 7.53 – 7.49 (m, 1H), 7.47 – 7.46 (m, 1H), 7.46 – 7.40 (m, 3H), 6.66 (d, *J* = 3.4 Hz, 1H), 6.44 (dd, *J* = 3.4, 1.8 Hz, 1H)

^{13}C NMR (101 MHz, CDCl$_3$, ppm): δ 189.7, 151.6, 145.0, 138.1, 132.8, 130.7, 128.6 (2C), 128.4 (2C), 119.2, 116.4, 112.8.

GC-MS (EI, 70 eV) (*m/z*, relative abundance %): 105 (100), 77 (83), 39 (78), 65 (54), 51 (53), 198 (49).

R$_f$ = 0.48 (*Hexanes*/EtOAc, 9:1).

(*E*)-1-(4-fluorophenyl)-3-(furan-2-yl)prop-2-en-1-one (3b)

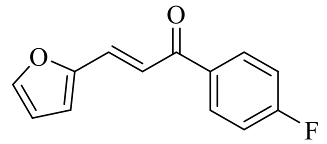

Prepared according to **GP 1** from furan (1 mmol, 1 equiv., 0.072 mL), *n*-BuLi (1.05 mmol, 1.05 equiv., 0.44 mL, 2.35 M), *N,N*-dimethylformamide (DMF) (1.1 mmol, 1.1 equiv., 0.08 mL), and 4′-fluoroacetophenone (1.0 mmol, 1.0 equiv., 0.12 mL). The crude material was purified by column chromatography on silica gel in a mixture of *Hexanes* and EtOAc (9:1, v/v). **CAS Nº:** 1565-90-8. Yellow solid, 1 mmol scale (104 mg, 48%).

^{1}H NMR (400 MHz, CDCl$_3$, ppm): δ 8.08 – 8.04 (m, 2H), 7.59 (d, *J* = 15.3 Hz, 1H), 7.55 – 7.50 (m, 1H), 7.42 (d, *J* = 15.3 Hz, 1H), 7.17 (app t, *J* = 9.0 Hz, 2H), 6.73 (d, *J* = 3.4 Hz, 1H), 6.52 (dd, *J* = 3.4, 1.8 Hz, 1H).

^{13}C NMR (101 MHz, CDCl$_3$, ppm): δ 188.1, 166.9, 164.4, 151.6, 145.0, 134.5 (d, $J_{C\text{-}F}$ = 3.0 Hz), 130.9 (d, $J_{C\text{-}F}$ = 9.2 Hz), 130.8, 118.9, 116.3, 115.8, 115.6, 112.7.

GC-MS (EI, 70 eV) (*m/z*, relative abundance %): 123 (100), 95 (51), 216 (41), 65 (35), 39 (32), 159 (26).

R$_f$ = 0.43 (*Hexanes*/EtOAc, 9:1).

MP = 65.3 – 67.2ºC.

(*E*)-1-(4-chlorophenyl)-3-(furan-2-yl)prop-2-en-1-one (3c)

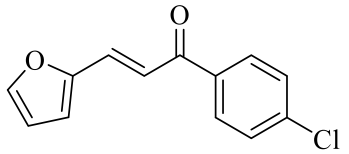

Prepared according to **GP 1** from furan (1 mmol, 1 equiv., 0.072 mL), *n*-BuLi (1.05 mmol, 1.05 equiv., 0.44 mL, 2.35 M), *N,N*-dimethylformamide (DMF) (1.1 mmol, 1.1 equiv., 0.08 mL), and 4′-chloroacetophenone (1.0 mmol, 1.0 equiv., 154.6 mg). The crude material was purified by column chromatography on silica gel in a mixture of *Hexanes* and EtOAc (9:1, v/v). **CAS Nº:** 14385-65-0. Yellow solid, 1 mmol scale (106 mg, 46%).

^{1}H NMR (400 MHz, CDCl$_3$, ppm): δ 7.97 (app d, *J* = 8.8 Hz, 2H), 7.60 (d, *J* = 15.3 Hz, 1H), 7.55 – 7.52 (m, 1H), 7.47 (app d, *J* = 8.8 Hz, 2H), 7.41 (d, *J* = 15.3 Hz, 1H), 6.74 (d, *J* = 3.4 Hz, 1H), 6.53 (dd, *J* = 3.4, 1.8 Hz, 1H).

^{13}C NMR (101 MHz, CDCl$_3$, ppm): δ 188.5, 151.6, 145.1, 139.2, 136.5, 131.1, 129.8 (2C), 128.9 (2C), 118.8, 116.5, 112.8.

GC-MS (EI, 70 eV) (*m/z*, relative abundance %): 139 (100), 39 (67), 65 (64), 141 (54), 75(47), 232 (33).

$\mathbf{R}_f$ = 0.53 (*Hexanes*/EtOAc, 9:1).

MP = 68.6 – 70.3ºC.

(*E*)-4-(3-(furan-2-yl)acryloyl)benzonitrile (3d)[3]

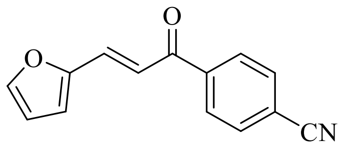

Prepared according to **GP 1** from furan (1 mmol, 1 equiv., 0.072 mL), *n*-BuLi (1.05 mmol, 1.05 equiv., 0.44 mL, 2.35 M), *N,N*-dimethylformamide (DMF) (1.1 mmol, 1.1 equiv., 0.08 mL), and 4-acetylbenzonitrile (1.0 mmol, 1.0 equiv., 145.2 mg). The crude material was purified by column chromatography on silica gel in a mixture of *Hexanes* and EtOAc (6:4, v/v). Orange solid, 1 mmol scale (55 mg, 25%).

^{1}H NMR (400 MHz, CDCl$_3$, ppm): δ 8.08 (app d, *J* = 8.4 Hz, 2H), 7.79 (app d, *J* = 8.6 Hz, 2H), 7.62 (d, *J* = 15.3 Hz, 1H), 7.58 – 7.54 (m, 1H), 7.38 (d, *J* = 15.3 Hz, 1H), 6.78 (d, *J* = 3.4 Hz, 1H), 6.54 (dd, *J* = 3.4, 1.8 Hz, 1H).

^{13}C NMR (101 MHz, CDCl$_3$, ppm): δ 188.3, 151.3, 145.5, 141.4, 132.5 (2C), 132.0, 128.8 (2C), 118.3, 118.0, 117.4, 115.9, 113.0.

GC-MS (EI, 70 eV) (*m/z*, relative abundance %): 39 (100), 130 (97), 65 (79), 102 (58), 75 (27), 223 (23).

$\mathbf{R}_f$ = 0.46 (*Hexanes*/EtOAc, 6:4).

MP = 134.2 – 136.0ºC.

(*E*)-3-(furan-2-yl)-1-(4-nitrophenyl)prop-2-en-1-one (3e)

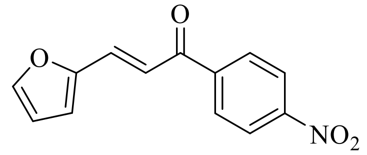

Prepared according to **GP 1** from furan (1 mmol, 1 equiv., 0.072 mL), *n*-BuLi (1.05 mmol, 1.05 equiv., 0.44 mL, 2.35 M), *N,N*-Dimethylformamide (DMF) (1.1 mmol, 1.1 equiv., 0.08 mL), and 4′-Nitroacetophenone (1.0 mmol, 1.0 equiv., 165.1 mg). The crude material was purified by column chromatography on silica gel in a mixture of *Hexanes* and EtOAc (7:3, v/v). **CAS Nº:** 4332-89-2. Orange solid, 1 mmol scale (85 mg, 35%).

^{1}H NMR (400 MHz, CDCl$_3$, ppm): δ 8.34 (app d, *J* = 8.8 Hz, 2H), 8.15 (app d, *J* = 8.8 Hz, 2H), 7.63 (d, *J* = 15.2 Hz, 1H), 7.57 (d, *J* = 1.2 Hz, 1H), 7.40 (d, *J* = 15.2 Hz, 1H), 6.80 (d, *J* = 3.4 Hz, 1H), 6.55 (dd, *J* = 3.4, 1.8 Hz, 1H).

^{13}C NMR (101 MHz, CDCl$_3$, ppm): δ 188.2, 151.3, 150.1, 145.6, 143.0, 132.1, 129.3 (2C), 123.8 (2C), 118.4, 117.6, 113.0.

GC-MS (EI, 70 eV) (*m/z*, relative abundance %):150 (100), 65 (82), 39 (50), 121 (42), 76 (34), 243 (31).

R*f* = 0.55 (*Hexanes*/EtOAc, 7:3).

MP = 147.6 – 149.3ºC.

(*E*)-3-(furan-2-yl)-1-(4-(trifluoromethyl)phenyl)prop-2-en-1-one (3f)

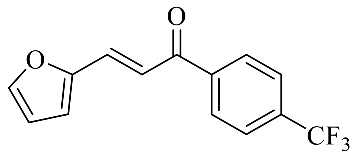

Prepared according to **GP 1** from furan (1 mmol, 1 equiv., 0.072 mL), *n*-BuLi (1.05 mmol, 1.05 equiv., 0.44 mL, 2.35 M), *N,N*-dimethylformamide (DMF) (1.1 mmol, 1.1 equiv., 0.08 mL), and 4′-(trifluoromethyl)acetophenone (1.0 mmol, 1.0 equiv., 188.1 mg). The crude material was purified by column chromatography on silica gel in a mixture of *Hexanes* and EtOAc (9:1, v/v). **CAS Nº:** 339018-12-1. Orange solid, 1 mmol scale (98 mg, 37%).

^{1}H NMR (400 MHz, CDCl3, ppm): δ 8.11 (d, *J* = 8.6 Hz, 2H), 7.75 (d, *J* = 8.6 Hz, 2H), 7.62 (d, *J* = 15.3 Hz, 1H), 7.55 (m, 1H), 7.41 (d, *J* = 15.3 Hz, 1H), 6.77 (d, *J* = 3.4 Hz, 1H), 6.53 (dd, *J* = 3.4, 1.8 Hz, 1H).

^{13}C NMR (101 MHz, CDCl3, ppm): δ 188.8, 151.4, 145.3, 141.0, 134.0 (q, J_{C-F} = 32 Hz), 131.6, 128.7(2C), 125.6 (q, J_{C-F} = 3 Hz), 125.1, 122.3, 118.8, 117.0, 112.8.

GC-MS (EI, 70 eV) (*m/z*, relative abundance %): 173 (100), 39 (97), 65 (72), 145 (60), 266 (35), 121 (32).

R*f* = 0.41 (*Hexanes*/EtOAc, 9:1).

MP = 74.9 – 75.9ºC.

(*E*)-3-(furan-2-yl)-1-(p-tolyl)prop-2-en-1-one (3g)

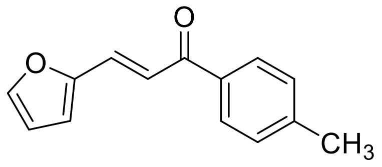

Prepared according to **GP 1** from furan (1 mmol, 1 equiv., 0.072 mL), *n*-BuLi (1.05 mmol, 1.05 equiv., 0.44 mL, 2.35 M), *N,N*-dimethylformamide (DMF) (1.1 mmol, 1.1 equiv., 0.08 mL), and 4′-methylacetophenone (1.0 mmol, 1.0 equiv., 0.13 mL). The crude material was purified by column chromatography on silica gel in a mixture of *Hexanes* and EtOAc (9:1, v/v). **CAS Nº:** 14385-63-8. Orange solid, 1 mmol scale (116 mg, 55%).

^{1}H NMR (400 MHz, CDCl3, ppm): δ 7.85 (app d, *J* = 8.8 Hz, 2H), 7.50 (d, *J* = 15.3 Hz, 1H), 7.43 – 7.42 (m, 1H), 7.37 (d, *J* = 15.3 Hz, 1H), 7.20 (app d, J = 8.0 Hz, 2H), 6.61 (d, *J* = 3.4 Hz, 1H), 6.41 (dd, *J* = 3.4, 1.8 Hz, 1H), 2.33 (s, 3H).

^{13}C NMR (101 MHz, CDCl3, ppm): δ 189.4, 151.9, 144.9, 143.7, 135.7, 130.4, 129.4 (2C), 128.7 (2C), 119.6, 116.0, 112.7, 21.7.

GC-MS (EI, 70 eV) (*m/z*, relative abundance %): 39 (100), 65 (99), 119 (94), 91 (74), 212 (58), 63 (30).

$\mathbf{R_f}$ = 0.72 (*Hexanes*/EtOAc, 7:3).

MP = 59.4 – 61.7ºC.

(*E*)-3-(furan-2-yl)-1-(4-methoxyphenyl)prop-2-en-1-one (3h)

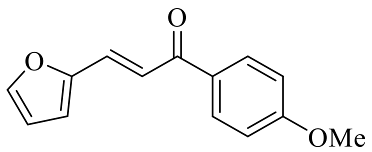

Prepared according to **GP 1** from furan (1 mmol, 1 equiv., 0.072 mL), *n*-BuLi (1.05 mmol, 1.05 equiv., 0.44 mL, 2.35 M), *N,N*-dimethylformamide (DMF) (1.1 mmol, 1.1 equiv., 0.08 mL), and 4′-methoxyacetophenone (1.0 mmol, 1.0 equiv.,150.1 mg). The crude material was purified by column chromatography on silica gel in a mixture of *Hexanes* and EtOAc (7:3, v/v). **CAS Nº:** 5066-65-9. Yellow solid, 1 mmol scale (185 mg, 81%).

^{1}H NMR (400 MHz, CDCl$_3$, ppm): δ 8.04 (app d, *J* = 8.9 Hz, 2H), 7.58 (d, *J* = 15.3 Hz, 1H), 7.52 (d, *J* = 1.4 Hz, 1H), 7.47 (d, *J* = 15.3 Hz, 1H), 6.97 (app d, *J* = 8.9 Hz, 2H), 6.69 (d, *J* = 3.4 Hz, 1H), 6.50 (dd, *J* = 3.4, 1.8 Hz, 1H), 3.88 (s, 3H).

^{13}C NMR (101 MHz, CDCl$_3$, ppm): δ 188.1, 163.5, 151.9, 144.6, 131.2, 130.7 (2C), 130.0, 119.3, 115.6, 113.8 (2C), 112.6, 55.5.

GC-MS (EI, 70 eV) (*m/z*, relative abundance %): 135 (100), 228 (63), 77 (51), 65 (49), 39 (44), 92 (38).

$\mathbf{R_f}$ = 0.63 (*Hexanes*/EtOAc, 7:3).

MP = 78.9 – 80.5ºC.

(*E*)-1-(3,4-dimethoxyphenyl)-3-(furan-2-yl)prop-2-en-1-one (3i)

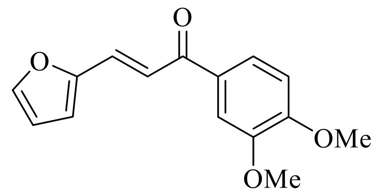

Prepared according to **GP 1** from furan (1 mmol, 1 equiv., 0.072 mL), *n*-BuLi (1.05 mmol, 1.05 equiv., 0.44 mL, 2.35 M), *N,N*-dimethylformamide (DMF) (1.1 mmol, 1.1 equiv., 0.08 mL), and 3′,4′-dimethoxyacetophenone (1.0 mmol, 1.0 equiv., 180.2 mg). The crude material was purified by column chromatography on silica gel in a mixture of *Hexanes* and EtOAc (7:3, v/v). **CAS Nº:** 4538-04-9. Yellow solid, 1 mmol scale (157 mg, 61%).

^{1}H NMR (400 MHz, CDCl$_3$, ppm): δ 7.70 (dd, *J* = 8.4, 2.0 Hz, 1H), 7.62 (d, *J* = 2.0 Hz, 1H), 7.59 (d, *J* = 15.3 Hz, 1H), 7.52 (d, *J* = 1.5 Hz, 1H), 7.48 (d, *J* = 15.3 Hz, 1H), 6.92 (d, *J* = 8.4 Hz, 1H), 6.71 (d, *J* = 3.4 Hz, 1H), 6.51 (dd, *J* = 3.4, 1.8 Hz, 1H), 3.97 (d, *J* = 2.1 Hz, 6H).

^{13}C NMR (101 MHz, CDCl$_3$, ppm): δ 188.0, 153.3, 151.9, 149.3, 144.7, 131.4, 130.0, 123.0, 119.1, 115.7, 112.6, 110.9, 110.1, 56.1, 56.1.

GC-MS (EI, 70 eV) (*m/z*, relative abundance %): 258 (100) 165 (77), 65 (62), 121 (43), 39 (37), 79 (33).

R*f* = 0.42 (*Hexanes*/EtOAc, 7:3).

MP = 74.5 – 76.6ºC.

(*E*)-3-(furan-2-yl)-1-(naphthalen-2-yl)prop-2-en-1-one (3j)

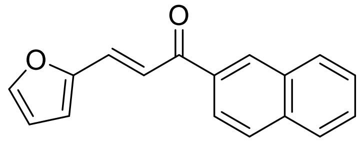

Prepared according to **GP 1** from furan (1 mmol, 1 equiv., 0.072 mL), *n*-BuLi (1.05 mmol, 1.05 equiv., 0.44 mL, 2.35 M), *N,N*-dimethylformamide (DMF) (1.1 mmol, 1.1 equiv., 0.08 mL), and 2-acetonaphthone (1.0 mmol, 1.0 equiv., 170.2 mg). The crude material was purified by column chromatography on silica gel in a mixture of *Hexanes* and EtOAc (7:3, v/v). **CAS Nº:** 15462-59-6. Orange solid, 1 mmol scale (170 mg, 68%).

^{1}H NMR (400 MHz, CDCl$_3$, ppm): δ 8.56 (bs, 1H), 8.12 (dd, *J* = 8.6, 1.8 Hz, 1H), 8.00 (d, *J* = 8.0 Hz, 1H), 7.93 (d, *J* = 8.7 Hz, 1H), 7.89 (d, *J* = 8.0 Hz, 1H), 7.65 (d, *J* = 4.6 Hz, 2H), 7.63 – 7.54 (m, 3H), 6.75 (d, *J* = 3.4 Hz, 1H), 6.53 (dd, *J* = 3.4, 1.8 Hz, 1H).

^{13}C NMR (101 MHz, CDCl$_3$, ppm): δ 189.6, 151.8, 144.9, 135.6, 135.5, 132.6, 130.6, 129.9, 129.6, 128.5, 128.3, 127.8, 126.7, 124.4, 119.5, 116.2, 112.7.

GC-MS (EI, 70 eV) (*m/z*, relative abundance %): 127 (100), 155 (93), 248 (80), 65 (57), 39 (40), 191 (34).

R*f* = 0.70 (*Hexanes*/EtOAc, 7:3).

MP = 85.3 – 87.9ºC.

(*E*)-1,3-di(furan-2-yl)prop-2-en-1-one (3k)

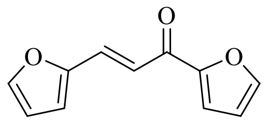

Prepared according to **GP 1** from furan (1 mmol, 1 equiv., 0.072 mL), *n*-BuLi (1.05 mmol, 1.05 equiv., 0.44 mL, 2.35 M), *N,N*-dimethylformamide (DMF) (1.1 mmol, 1.1 equiv., 0.08 mL), and 2-furyl methyl ketone (1.0 mmol, 1.0 equiv., 110.1 mg). The crude material was purified by column chromatography on silica gel in a mixture of *Hexanes* and EtOAc (7:3, v/v). **CAS Nº:** 3988-76-9. White solid, 1 mmol scale (131 mg, 70%).

^{1}H NMR (400 MHz, CDCl$_3$, ppm): δ 7.61 (d, *J* = 15.6 Hz, 2H), 7.51 (d, *J* = 1.6 Hz, 1H), 7.31 (d, *J* = 8.8 Hz, 1H), 7.29 (d, *J* = 3.2 Hz, 1H), 6.70 (d, *J* = 3.4 Hz, 1H), 6.56 (dd, *J* = 3.6, 1.6 Hz, 1H), 6.49 (dd, *J* = 3.2, 1.6 Hz, 1H).

^{13}C NMR (101 MHz, CDCl$_3$, ppm): δ 177.7, 153.8, 151.6, 146.5, 145.0, 129.8, 118.9, 117.3, 116.2, 112.7, 112.4.

GC-MS (EI, 70 eV) (*m/z*, relative abundance %): 95 (100), 39 (77), 188 (49), 65 (45), 131 (34), 103 (21).

$\mathbf{R}_f$ = 0.60 (*Hexanes*/EtOAc, 7:3).

MP = 87.7 – 90.1ºC.

(*E*)-3-(furan-2-yl)-1-(1H-pyrrol-2-yl)prop-2-en-1-one (3l)

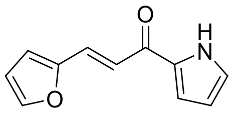

Prepared according to **GP1** from furan (1 mmol, 1 equiv., 0.072 mL), *n*-BuLi (1.05 mmol, 1.05 equiv., 0.44 mL, 2.35 M), *N,N*-Dimethylformamide (DMF) (1.1 mmol, 1.1 equiv., 0.08 mL) and N-Boc-acetopyrrole (1.0 mmol, 1.0 equiv., 209.2 mg). The crude was purified by column chromatography on silica gel in a mixture of *Hexanes* and EtOAc (7:3, v/v). **CAS Nº**: 4911-94-8. Orange-brown solid, 1 mmol scale (116 mg, 40%).

^{1}H NMR (500 MHz, CD_3OD, ppm): δ 7.66 (app d, *J* = 1.4 Hz, 1H), 7.50 (d, *J* = 15.5 Hz, 1H), 7.33 (d, *J* = 15.5 Hz, 1H), 7.17 – 7.14 (m, 2H), 6.82 (d, *J* = 3.4 Hz, 1H), 6.57 (dd, *J* = 3.4, 1.8 Hz, 1H), 6.31 (dd, *J* = 3.8, 2.5 Hz, 1H).

^{13}C NMR (125 MHz, CD_3OD, ppm): δ 180.3, 153.1, 146.4, 134.3, 129.3, 127.6, 120.9, 118.6, 116.5, 113.7, 111.7.

GC-MS (EI, 70 eV) (*m/z*, relative abundance %): 187 (100), 94 (77), 130 (47), 158 (27), 66 (23), 133 (22).

$\mathbf{R}_f$ = 0.43 (*Hexanes*/EtOAc, 7:3).

MP = 110.2 – 113.7ºC.

(*E*)-1-(3-chlorophenyl)-3-(furan-2-yl)prop-2-en-1-one (3m)

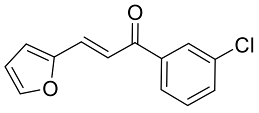

Prepared according to **GP1** from furan (1 mmol, 1 equiv., 0.072 mL), *n*-BuLi (1.05 mmol, 1.05 equiv., 0.44 mL, 2.35 M), *N,N*-Dimethylformamide (DMF) (1.1 mmol, 1.1 equiv., 0.08 mL) and 3-chloroacetophenone (1.0 mmol, 1.0 equiv., 154.6 mg). The crude was purified by column chromatography on silica gel in a mixture of *Hexanes* and EtOAc (9:1, v/v). **CAS Nº**: 1213779-51-1. Bright-yellow solid, 2 mmol scale (123 mg, 26%).

^{1}H NMR (400 MHz, $CDCl_3$, ppm): δ 7.99 (app t, *J* = 1.9 Hz, 1H), 7.89 (dt, *J* = 7.9, 1.3 Hz, 1H), 7.60 (d, *J* = 15.3 Hz, 1H), 7.55 – 7.52 (m, 2H), 7.43 (t, *J* = 7.9 Hz, 1H), 7.38 (d, *J* = 15.3 Hz, 1H), 6.75 (d, *J* = 3.4 Hz, 1H), 6.52 (dd, *J* = 3.5, 1.8 Hz, 1H).

^{13}C NMR (101 MHz, $CDCl_3$, ppm): δ 188.5, 151.6, 145.4, 139.8, 135.0, 132.8, 131.4, 130.1, 128.6, 126.6, 118.7, 117.0, 112.9.

GC-MS (EI, 70 eV) (*m/z*, relative abundance %): 139 (100), 232 (64), 141 (48), 121 (39), 65 (28), 111 (26).

R_f = 0.42 (*Hexanes*/EtOAc, 9:1).

MP = 48.4 – 50.2ºC.

(*E*)-1-(3-fluorophenyl)-3-(furan-2-yl)prop-2-en-1-one (3n)

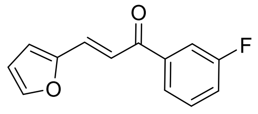

Prepared according to **GP1** from furan (1 mmol, 1 equiv., 0.072 mL), *n*-BuLi (1.05 mmol, 1.05 equiv., 0.44 mL, 2.35 M), *N,N*-Dimethylformamide (DMF) (1.1 mmol, 1.1 equiv., 0.08 mL) and 3-fluoroacetophenone (1.0 mmol, 1.0 equiv., 138.1 mg). The crude was purified by column chromatography on silica gel in a mixture of *Hexanes* and EtOAc (9:1, v/v). **CAS Nº**: 24090-01-5. Orange-yellow solid, 2 mmol scale (136 mg, 31%).

^{1}H NMR (400 MHz, $CDCl_3$, ppm): δ 7.81 (dt, *J* = 7.7, 1.2 Hz, 1H), 7.71 (ddd, *J* = 9.5, 2.6, 1.5 Hz, 1H), 7.61 (d, *J* = 15.3 Hz, 1H), 7.55 (app d, *J* = 1.8 Hz, 1H), 7.48 (td, *J* = 8.0, 5.5 Hz, 1H), 7.40 (d, *J* = 15.3 Hz, 1H), 7.27 (tdd, *J* = 8.3, 2.6, 1.0 Hz, 1H), 6.75 (d, *J* = 3.4 Hz, 1H), 6.53 (dd, *J* = 3.4, 1.8 Hz, 1H).

^{13}C NMR (101 MHz, $CDCl_3$, ppm): δ 188.6 (d, $^4J_{C\text{-}F}$ = 2.2 Hz), 163.0 (d, $^1J_{C\text{-}F}$ = 247.7 Hz), 151.6, 145.3, 140.4 (d, $^3J_{C\text{-}F}$ = 6.3 Hz), 131.4, 130.4 (d, $^3J_{C\text{-}F}$ = 7.7 Hz), 124.2 (d, $^4J_{C\text{-}F}$ = 3 Hz), 119.9 (d, $^2J_{C\text{-}F}$ = 21.5 Hz), 118.8, 117.0, 115.3 (d, $^2J_{C\text{-}F}$ = 22.4 Hz), 112.9.

GC-MS (EI, 70 eV) (*m/z*, relative abundance %): 123 (100), 216 (67), 95 (30), 121 (30), 65 (21), 159 (20).

R_f = 0.41 (*Hexanes*/EtOAc, 9:1).

MP = 60.6 – 62.3ºC.

(*E*)-1-(3-bromophenyl)-3-(furan-2-yl)prop-2-en-1-one (3o)

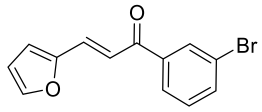

Prepared according to **GP1** from furan (1 mmol, 1 equiv., 0.072 mL), *n*-BuLi (1.05 mmol, 1.05 equiv., 0.44 mL, 2.35 M), *N,N*-Dimethylformamide (DMF) (1.1 mmol, 1.1 equiv., 0.08 mL) and 3-bromoacetophenone (1.0 mmol, 1.0 equiv., 199.0 mg). **CAS Nº**: 1213779-53-3. The crude was purified by column chromatography on silica gel in a mixture of *Hexanes* and EtOAc (9:1, v/v). Pale yellow solid, 2 mmol scale (111 mg, 20%).

^{1}H NMR (400 MHz, $CDCl_3$, ppm): δ 8.14 (app t, *J* = 1.9 Hz, 1H), 7.94 (dt, *J* = 7.8, 1.3 Hz, 1H), 7.71 – 7.67 (m, 1H), 7.60 (d, *J* = 15.2 Hz, 1H), 7.54 (d, *J* = 1.8 Hz, 1H), 7.41 – 7.35 (m, 2H), 6.75 (d, *J* = 3.5 Hz, 1H), 6.53 (dd, *J* = 3.5, 1.8 Hz, 1H).

^{13}C NMR (101 MHz, $CDCl_3$, ppm): δ 188.4, 151.6, 145.4, 140.0, 135.7, 131.5 (d, *J* = 8.5 Hz), 130.3, 127.0, 123.1, 118.6, 117.1, 113.0.

GC-MS (EI, 70 eV) (*m/z*, relative abundance %): 185 (100), 183 (88), 276 (56), 121 (54), 278 (49), 157 (20).

R$_f$ = 0.47 (*Hexanes*/EtOAc, 9:1).

MP = 99.6 – 100.8ºC.

(*E*)-3-(furan-2-yl)-1-(2-nitrophenyl)prop-2-en-1-one (3p)

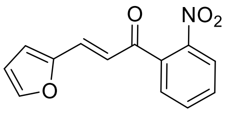

Prepared according to **GP1** from furan (1 mmol, 1 equiv., 0.072 mL), *n*-BuLi (1.05 mmol, 1.05 equiv., 0.44 mL, 2.35 M), *N,N*-Dimethylformamide (DMF) (1.1 mmol, 1.1 equiv., 0.08 mL) and 2-nitroacetophenone (1.0 mmol, 1.0 equiv., 165.1 mg). The crude was purified by column chromatography on silica gel in a mixture of *Hexanes* and EtOAc (7:3, v/v). **CAS Nº**: 158117-48-7. Red-brown solid, 2 mmol scale (349 mg, 71%).

^{1}H NMR (400 MHz, CDCl$_3$, ppm): δ 8.14 (dd, *J* = 8.2, 1.2 Hz, 1H), 7.74 (td, *J* = 7.5, 1.2 Hz, 1H), 7.70 – 7.59 (m, 1H), 7.54 – 7.46 (m, 2H), 7.06 (d, *J* = 15.9 Hz, 1H), 6.86 (d, *J* = 15.8 Hz, 1H), 6.66 (d, *J* = 3.4 Hz, 1H), 6.48 (dd, *J* = 3.5, 1.8 Hz, 1H).

^{13}C NMR (101 MHz, CDCl$_3$, ppm): δ 192.4, 150.6, 146.8, 145.8, 136.4, 134.1, 132.1, 130.7, 128.9, 124.7, 123.3, 117.2, 113.0.

GC-MS (EI, 70 eV) (*m/z*, relative abundance %): 104 (100), 81 (45), 65 (43), 134 (31), 121 (30), 76 (28).

R$_f$ = 0.37 (*Hexanes*/EtOAc, 7:3).

MP = 92.6 – 94.8ºC.

(*E*)-3-(furan-2-yl)-1-(3-nitrophenyl)prop-2-en-1-one (3q)

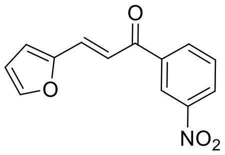

Prepared according to **GP1** from furan (1 mmol, 1 equiv., 0.072 mL), *n*-BuLi (1.05 mmol, 1.05 equiv., 0.44 mL, 2.35 M), *N,N*-Dimethylformamide (DMF) (1.1 mmol, 1.1 equiv., 0.08 mL) and 3-nitroacetophenone (1.0 mmol, 1.0 equiv., 165.1 mg). The crude was purified by column chromatography on silica gel in a mixture of *Hexanes* and EtOAc (9:1, v/v). **CAS Nº**: 15462-51-8. Pale yellow solid, 2 mmol scale (50 mg, 10%).

^{1}H NMR (400 MHz, CDCl$_3$, ppm): δ 8.84 (app t, *J* = 2.0 Hz, 1H), 8.42 (ddd, *J* = 8.2, 2.4, 1.1 Hz, 1H), 8.35 (dt, *J* = 7.8, 1.4 Hz, 1H), 7.71 (d, *J* = 8.0 Hz, 1H), 7.66 (d, *J* = 15.6 Hz, 1H), 7.58 (d, *J* = 1.7 Hz, 1H), 7.44 (d, *J* = 15.2 Hz, 1H), 6.80 (d, *J* = 3.4 Hz, 1H), 6.55 (dd, *J* = 3.4, 1.8 Hz, 1H).

^{13}C NMR (101 MHz, CDCl$_3$, ppm): δ 187.4, 151.4, 148.5, 145.8, 139.6, 134.1, 132.3, 130.0, 127.1, 123.3, 117.9 (d, *J* = 3.3 Hz), 113.1.

GC-MS (EI, 70 eV) (*m/z*, relative abundance %): 150 (100), 243 (37), 121 (31), 65 (20), 104 (15), 115 (9).

R$_f$ = 0.27 (*Hexanes*/EtOAc, 9:1).

MP = 64.7 – 66ºC.

2.8 General Procedure for the Preparation of the Heterocycle-Based Substrates used in the scope of Chalcones 5a-d (GP 2)

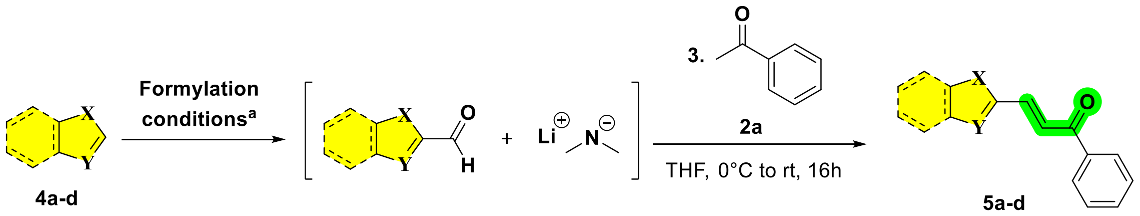

[a] From the literature

The procedures for the formylation of substrates used in the preparation of chalcones **5a-d** were based on methodologies available in the literature and are referenced for each example below. After the formation of formylated-heterocycles, the resulting mixture was cooled to 0 °C, and a solution of acetophenone (1.0 mmol, 1.0 equiv., 0.11 mL) in THF (1 mL) was added dropwise. The reaction was continued at room temperature and monitored by TLC accordingly to the substrate. The mixture was quenched with saturated $NH_4Cl_{(aq)}$ until the reaction medium clarified. The resulting biphasic mixture was extracted with EtOAc (3 × 15 mL), dried over $MgSO_4$, and then concentrated. The crude material was purified by column chromatography on silica gel based on the retention factor (R_f) of each product.

(*E*)-1-phenyl-3-(thiophen-2-yl)prop-2-en-1-one (5a)[4]

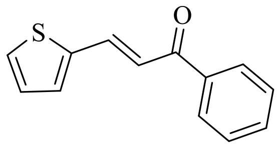

In a dry nitrogen-flushed round-bottom flask under magnetic stirring, thiophene (1 mmol, 1 equiv., 0.08 mL) in anhydrous THF (4 mL, 0.25 M) was kept at 0-5 °C using an ice bath. *n*-BuLi (1.05 mmol, 1.05 equiv., 0.44 mL, 2.35 M) was added dropwise, and the reaction was allowed to stir at the same temperature for 1 hour. Then, *N,N*-dimethylformamide (DMF) (1.0 mmol, 1.0 equiv., 0.08 mL) in THF (1 mL) was added in one portion. The ice bath was removed after 5 minutes, and the reaction was allowed to proceed at room temperature for 4 hours, affording the thiophene-2-carbaldehyde in situ. The chalcone was prepared using **GP 2**, from a solution of acetophenone (1.0 mmol, 1.0 equiv., 0.11 mL) in THF (1 mL). The crude material was purified by column chromatography on silica gel using a gradient of pure *Hexanes* to a mixture of *Hexanes* and EtOAc (9:1, v/v). **CAS Nº:** 2910-81-8. Yellow solid, 1 mmol scale (163 mg, 76%).

^{1}H NMR (400 MHz, $CDCl_3$, ppm): δ 8.02 – 7.98 (m, 2H), 7.95 (d, J = 15.3 Hz, 1H), 7.60 – 7.55 (m, 1H), 7.52 – 7.48 (m, 2H), 7.41 (d, J = 5.1 Hz, 1H), 7.36 (bs, 1H), 7.35 – 7.32 (d, J = 15.3 Hz, 1H), 7.08 (dd, J = 5.1, 3.7 Hz, 1H).

¹³C NMR (101 MHz, CDCl₃, ppm): δ 190.0, 140.5, 138.2, 137.3, 132.9, 132.2, 128.9, 128.7 (2C), 128.5 (2C), 128.5, 120.8.

GC-MS (EI, 70 eV) (m/z, relative abundance %): 214 (100), 137 (65), 77 (53), 185 (44), 109 (37), 65 (24).

R*f* = 0.46 (*Hexanes*/EtOAc, 6:4).

MP = 56.6 – 58.2°C.

(*E*)-3-(benzo[b]thiophen-2-yl)-1-phenylprop-2-en-1-one (5b)[5]

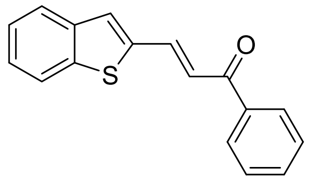

In a dry nitrogen-flushed round-bottom flask under magnetic stirring, benzothiophene (1 mmol, 1 equiv., 134 mg) in anhydrous THF (2 mL, 0.5 M) was kept at -78°C. *n*-BuLi (1.1 mmol, 1.1 equiv., 0.47 mL, 2.35 M) was added dropwise, and the reaction was allowed to stir at the same temperature for 1 hour. Then, *N,N*-dimethylformamide (DMF) (2.0 mmol, 2.0 equiv., 0.16 mL) in THF (1 mL) was added in one portion and kept at the same temperature for an additional 3 hours, affording the benzo[b]thiophene-2-carbaldehyde in situ. The chalcone was prepared using **GP 2**, from a solution of acetophenone (1.0 mmol, 1.0 equiv., 0.11 mL) in THF (1 mL). The crude material was purified by column chromatography on silica gel in a mixture of *Hexanes* and EtOAc (8:2, v/v). Yellow solid, 1 mmol scale (82 mg, 31%).

¹H NMR (400 MHz, CDCl₃, ppm): δ 8.07 – 8.00 (m, 3H), 7.83 – 7.77 (m, 2H), 7.63 – 7.58 (m, 1H), 7.57 – 7.49 (m, 3H), 7.43 – 7.36 (m, 3H).

¹³C NMR (101 MHz, CDCl₃, ppm): δ 189.6, 140.3, 140.3, 139.7, 138.0, 137.6, 132.9, 129.8, 128.7 (2C), 128.5 (2C), 126.5, 125.0, 124.5, 123.2, 122.5.

GC-MS (EI, 70 eV) (*m/z*, relative abundance %): 77 (100), 115 (91), 264 (67), 51 (50), 235 (47), 134 (31).

R*f* = 0.48 (*Hexanes*/EtOAc, 9:1).

MP = 110.2 – 113.1°C.

(*E*)-3-(benzofuran-2-yl)-1-phenylprop-2-en-1-one (5c)[6]

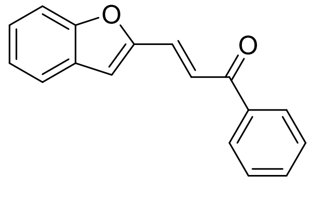

In a dry nitrogen-flushed round-bottom flask under magnetic stirring, benzofuran (1 mmol, 1 equiv., 0.11 mL) in anhydrous THF (5 mL, 0.20 M) was kept at -78°C. *n*-BuLi (1.2 mmol, 1.2 equiv., 0.51 mL, 2.35 M) was added dropwise, and the reaction was allowed to stir at the same temperature for 1 hour. Then, *N,N*-dimethylformamide (DMF) (2.0 mmol, 2.0 equiv., 0.16 mL) in THF (1 mL) was added in one portion and kept at the same temperature for an additional 4.5 hours, affording the benzofuran-2-carbaldehyde in situ. The chalcone was prepared using **GP 2**, from a

solution of acetophenone (1.0 mmol, 1.0 equiv., 0.11 mL) in THF (1 mL). The crude material was purified by column chromatography on silica gel in a mixture of *Hexanes* and EtOAc (8:2, v/v). **CAS Nº:** 79713-08-9. Yellow solid, 1 mmol scale (136 mg, 52%).

^{1}H NMR (300 MHz, $CDCl_3$, ppm): δ 8.01 (d, J = 7.4 Hz, 2H), δ 7.64 (s, 2H), δ 7.56 –7.49 (m, 2H), δ 7.48 – 7.41 (m, 3H), 7.31 (app t, J = 7.7 Hz, 1H), δ 7.18 (app t, J = 7.5 Hz, 1H), 6.96 (s, 1H).

^{13}C NMR (75 MHz, $CDCl_3$, ppm): δ 189.6, 155.6, 153.1, 137.9, 133.1, 130.9, 128.7 (2C), 128.6 (2C), 128.6, 126.8, 123.5, 121.9, 121.9, 112.6, 111.4.

GC-MS (EI, 70 eV) (*m/z*, relative abundance %): 77 (100), 248 (95), 115 (80), 219 (57), 171 (54), 51 (47).

R*f* = 0.60 (*Hexanes*/EtOAc, 9:1).

MP = 89.0 – 91.8°C.

(*E*)-3-(1-methyl-1H-imidazol-2-yl)-1-phenylprop-2-en-1-one (5d)[7]

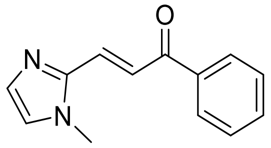

In a dry nitrogen-flushed round-bottom flask under magnetic stirring, 1-methyl-1*H*-imidazole (1 mmol, 1 equiv., 0.08 mL) in anhydrous THF (2 mL, 2 M) was kept at -78°C. *n*-BuLi (1.0 mmol, 1.0 equiv., 0.43 mL, 2.35 M) was added dropwise, and the reaction was allowed to stir at the same temperature for 5 min. Then, *N,N*-dimethylformamide (DMF) (2.0 mmol, 2.0 equiv., 0.16 mL) in THF (1 mL) was added in one portion and kept at the same temperature for an additional 1 hour and at 25°C for 40 minutes, affording the 1-methyl-1*H*-imidazole-2-carbaldehyde in situ. The chalcone was prepared using **GP 2**, from a solution of acetophenone (1.0 mmol, 1.0 equiv., 0.11 mL) in THF (1 mL). The crude material was purified by column chromatography on silica gel in a mixture of *Hexanes* and EtOAc (8:2, v/v). Brown solid, 1 mmol scale (180 mg, 85%).

^{1}H NMR (400 MHz, $CDCl_3$, ppm): δ 8.33 (d, J = 15.0 Hz, 1H), 8.17 – 8.15 (m, 2H), 7.66 (d, J = 15.0 Hz, 1H), 7.62 – 7.57 (m, 1H), 7.54 – 7.48 (m, 2H), 7.27 (bs, 1H), 7.06 (bs, 1H), 3.85 (s, 3H).

^{13}C NMR (101 MHz, $CDCl_3$, ppm): δ 189.2, 143.4, 137.5, 133.4, 128.9 (2C), 128.8 (2C), 128.6, 125.9, 125.6, 123.8, 33.4.

GC-MS (EI, 70 eV) (*m/z*, relative abundance %): 183 (100), 77 (34), 107 (20), 51 (19), 42 (19), 212 (10).

R*f* = 0.22 (*Hexanes*/EtOAc, 6:4).

MP = 110.0 – 111.8°C.

2.9 Synthetic applications for diversification of chalcone 3a

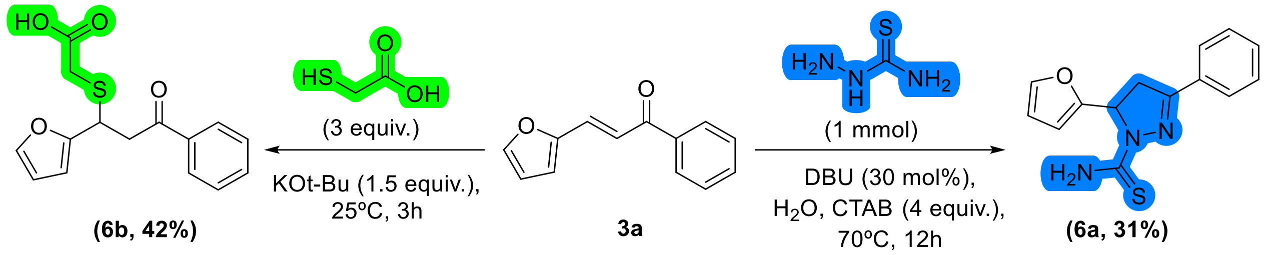

5-(furan-2-yl)-3-phenyl-4,5-dihydro-1H-pyrazole-1-carbothioamide (6a)[8,9]

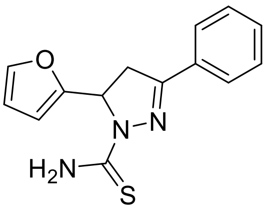

(*E*)-3-(Furan-2-yl)-1-phenylprop-2-en-1-one) (1 mmol), thiosemicarbazide (1 mmol), and DBU (30 mol%) were added to a homogeneous solution of CTAB (4 mmol) in water (10 mL), and the mixture was stirred at 70°C for 12h. Then, the reaction mixture was extracted with ethyl acetate and washed with water (3x20 mL). The organic phase was dried with $MgSO_4$, filtered, and evaporated under reduced pressure. The crude material was purified by chromatography on silica gel (automatic column – Biotage, 50g) in a mixture of *Hexanes* and EtOAc (7:3, v/v). Pale yellow solid, 1 mmol scale (84 mg, 31%).

^{1}H NMR (300 MHz, DMSO-d$_6$, ppm): δ 8.06 (bs, 1H), 7.91 – 7.88 (m, 2H), 7.85 (bs,1H), 7.52 (dd, *J* = 1.7, 0.7 Hz, 1H), 7.47 (dd, *J* = 5.2, 1.8 Hz, 3H), 6.38 (dd, *J* = 3.2, 1.8 Hz, 1H), 6.30 (bd, *J* = 3.1 Hz, 1H), 6.00 (dd, *J* = 11.4, 3.4 Hz, 1H), 3.78 (dd, *J* = 17.9, 11.5 Hz, 1H), 3.38 – 3.31 (m,1H).

^{13}C NMR (75 MHz, DMSO-d$_6$, ppm): δ 176.4, 155.6, 153.6, 142.5, 131.3, 131.1, 129.2 (2C), 127.6 (2C), 110.9, 107.9, 57.1, 39.1.

GC-MS (EI, 70 eV) (*m/z*, relative abundance %): 81 (100), 153 (94), 109 (93), 211 (58) 271 (81), 242 (36).

R$_f$ = 0.26 (*Hexanes*/EtOAc, 7:3)

MP = 203.7 – 205°C.

2-((1-(furan-2-yl)-3-oxo-3-phenylpropyl)thio)acetic acid (6b)[10]

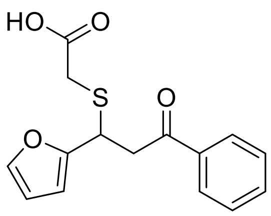

To a mixture of (E)-3-(furan-2-yl)-1-phenylprop-2-en-1-one (1 mmol) and thioglicolic acid (3 mmol) was added KOt-Bu (1.5 mmol), and the reaction mixture was stirred at room temperature for 3 h. After the reaction time, the mixture was extracted in dichloromethane, washed with diluted HCl (1M) and water (3x20 mL). Then, the organic phase was dried with $MgSO_4$, filtered, and evaporated under reduced pressure. The crude material was purified by chromatography on silica gel

(automatic column – Biotage, 25g) in a mixture of EtOAc and *Hexanes* (2:1, v/v). Pale yellow solid, 1 mmol scale (123 mg, 42%).

^{1}H NMR (300 MHz, $CDCl_3$, ppm): δ 9.31 (bs, 1H), 7.98 – 7.91 (m, 2H), 7.60 – 7.53 (m, 1H), 7.50 – 7.42 (m, 2H), 7.37 – 7.34 (m, 1H), 6.32 – 6.27 (m, 2H), 4.85 (dd, *J* = 7.8, 6.4 Hz, 1H), 3.76 (dd, *J* = 17.5, 7.9 Hz, 1H), 3.56 (dd, *J* = 17.5, 6.3 Hz, 1H), 3.23 (s, 2H).

^{13}C NMR (75 MHz, $CDCl_3$, ppm): δ 196.1, 176.0, 152.0, 142.6, 136.3, 133.6, 128.7 (2C), 128.2 (2C), 110.4, 108.4, 41.8, 37.8, 32.9.

HRMS: (m/z): $[M+Na]^{+}$: 313.0505, calc. 313.0511.

R_f = 0.25 (EtOAc/ *Hexanes*, 7:3)

MP = 94.1 – 95.0ºC.

3. Enzymatic Inhibition Assay

3.1 Enzymatic inhibition of papain

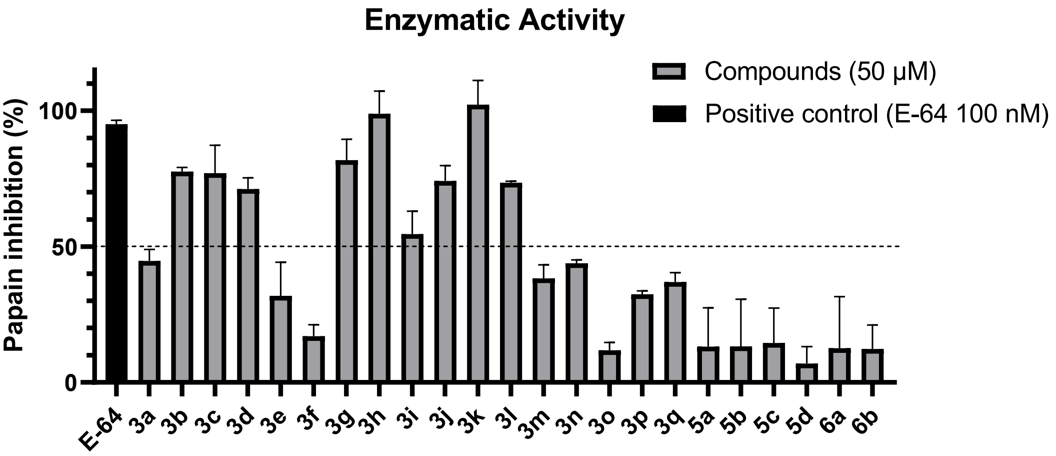

Figure S1. Enzymatic inhibition of papain by synthetic chalcone derivatives. The assay was performed in triplicate using Z-FR-MCA as the fluorogenic substrate. Enzyme activity was measured by the slope of fluorescence increase over time, and the percent inhibition was calculated by comparing each slope to that of the negative control (neat DMSO). E-64 (100 nM) was used as the positive control. The dashed line indicates the 50% inhibition threshold used to select compounds for IC_{50} determination. All chalcones were tested at a final concentration of 50 µM.

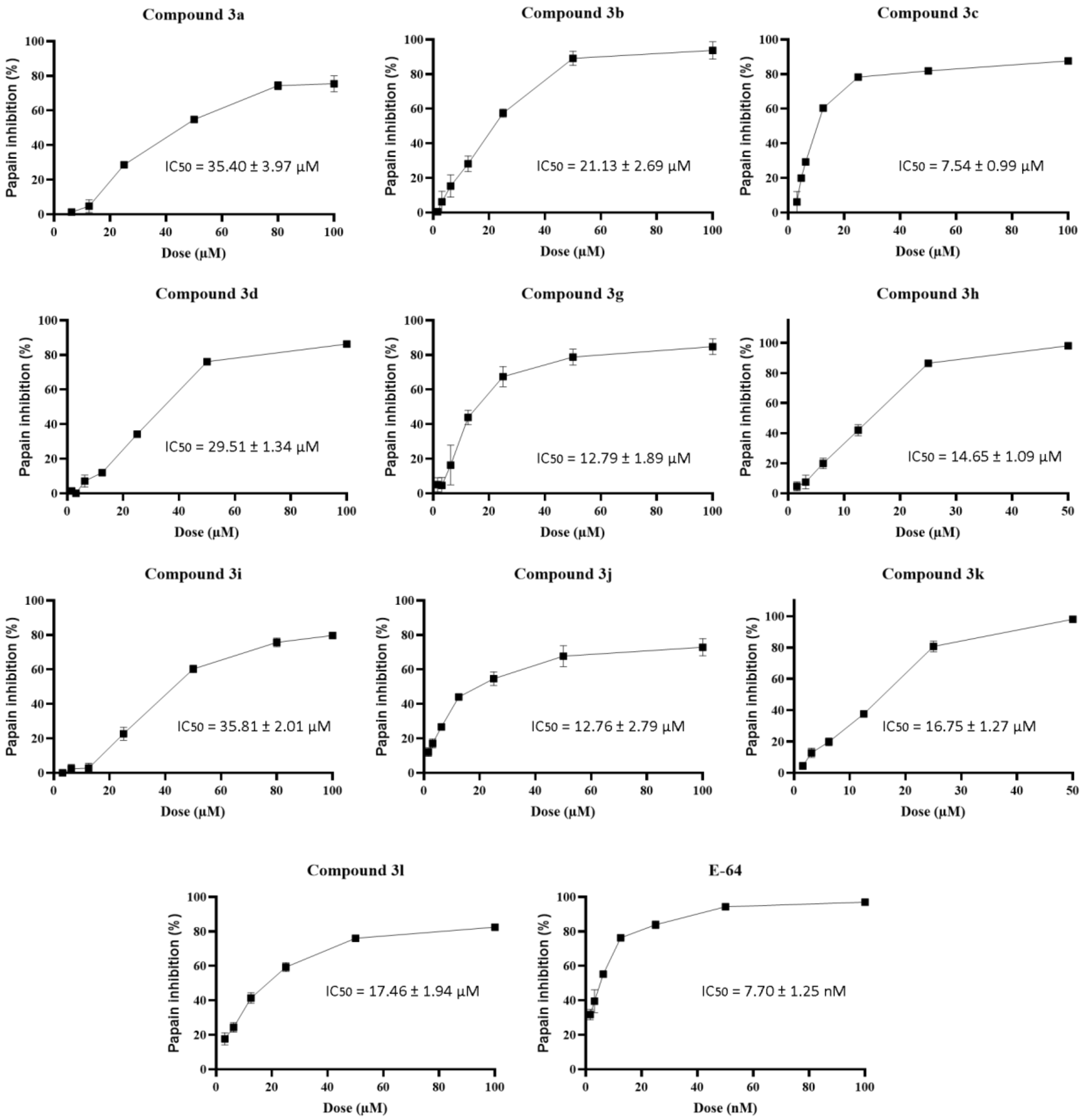

Figure S2. Determination of IC_{50} values for enzymatic inhibition of papain by chalcone derivatives **3a**, **3b**, **3c**, **3d**, **3g**, **3h**, **3i**, **3j**, **3k**, **3l** and positive control E-64. The IC_{50} values were calculated using nonlinear regression analysis based on the dose-response model. Each experiment was performed in triplicate.

3.2 Enzymatic inhibition of cathepsin B (CatB)

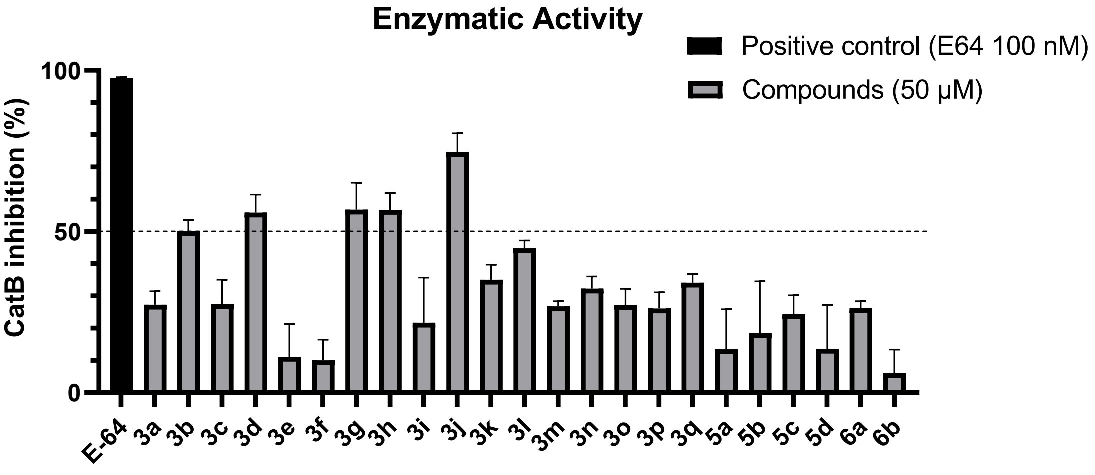

Figure S3. Enzymatic inhibition of cathepsin B by synthetic chalcone derivatives. The assay was performed in triplicate using Z-FR-MCA as the fluorogenic substrate. Enzyme activity was measured by the slope of fluorescence increase over time, and the percent inhibition was calculated by comparing each slope to that of the negative control (DMSO). E-64 (100 nM) was used as the positive control. The dashed line indicates the 50% inhibition threshold used to select compounds for IC_{50} determination. All chalcones were tested at a final concentration of 50 µM.

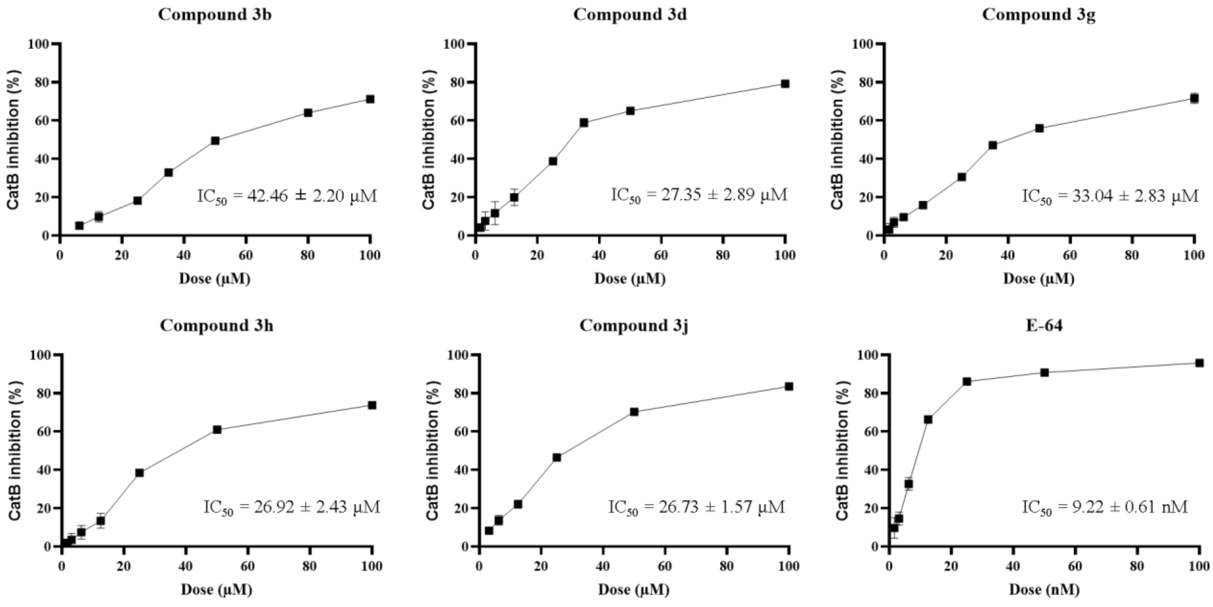

Figure S4. Determination of IC_{50} values for enzymatic inhibition of cathepsin B by chalcone derivatives **3b**, **3d**, **3g**, **3h**, **3j** and positive control E-64. The IC_{50} values were calculated using nonlinear regression analysis based on the dose-response model. Each experiment was performed in triplicate.

4. Molecular Docking

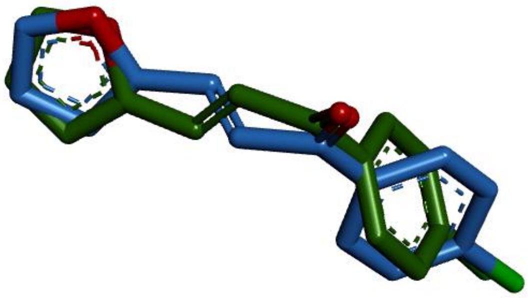

Figure S5. Structural alignment of compound 3c docked into Cathepsin B (PDB: 1CSB) and papain (PDB: 1BQI) performed using Discovery Studio. The green conformation represents the docking pose in CatB, while the blue conformation corresponds to the pose in papain. The alignment highlights the conformational differences between both complexes, suggesting a less favorable orientation of **3c** in the CatB active site.

5. NMR Spectra

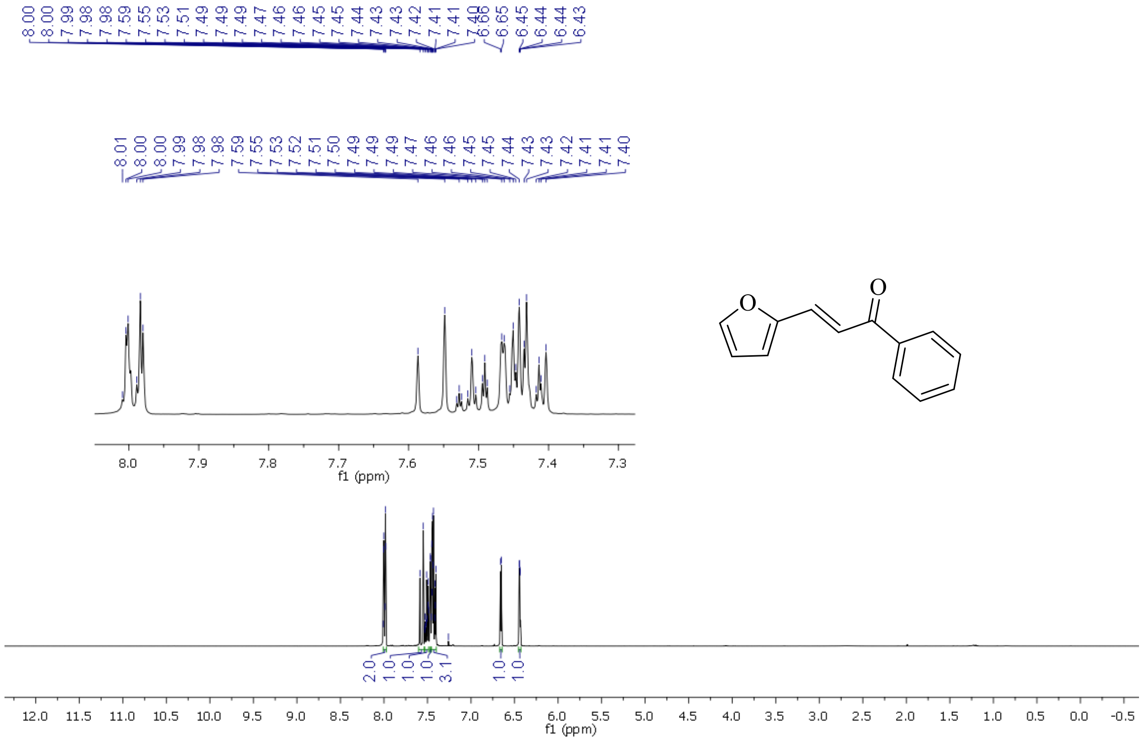

Figure S6. ^{1}H NMR (400 MHz, $CDCl_3$, ppm) of (*E*)-3-(furan-2-yl)-1-phenylprop-2-en-1-one (3a).

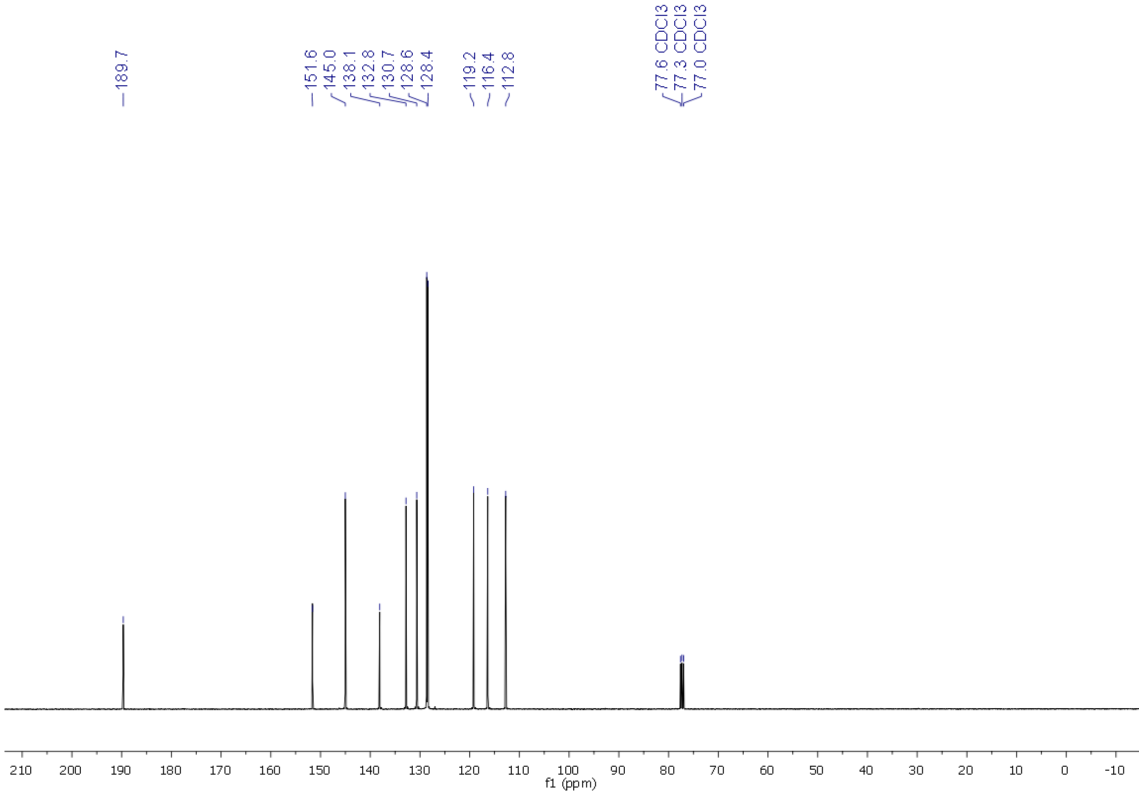

Figure S7. ^{13}C NMR (101 MHz, $CDCl_3$, ppm) of (*E*)-3-(furan-2-yl)-1-phenylprop-2-en-1-one (3a).

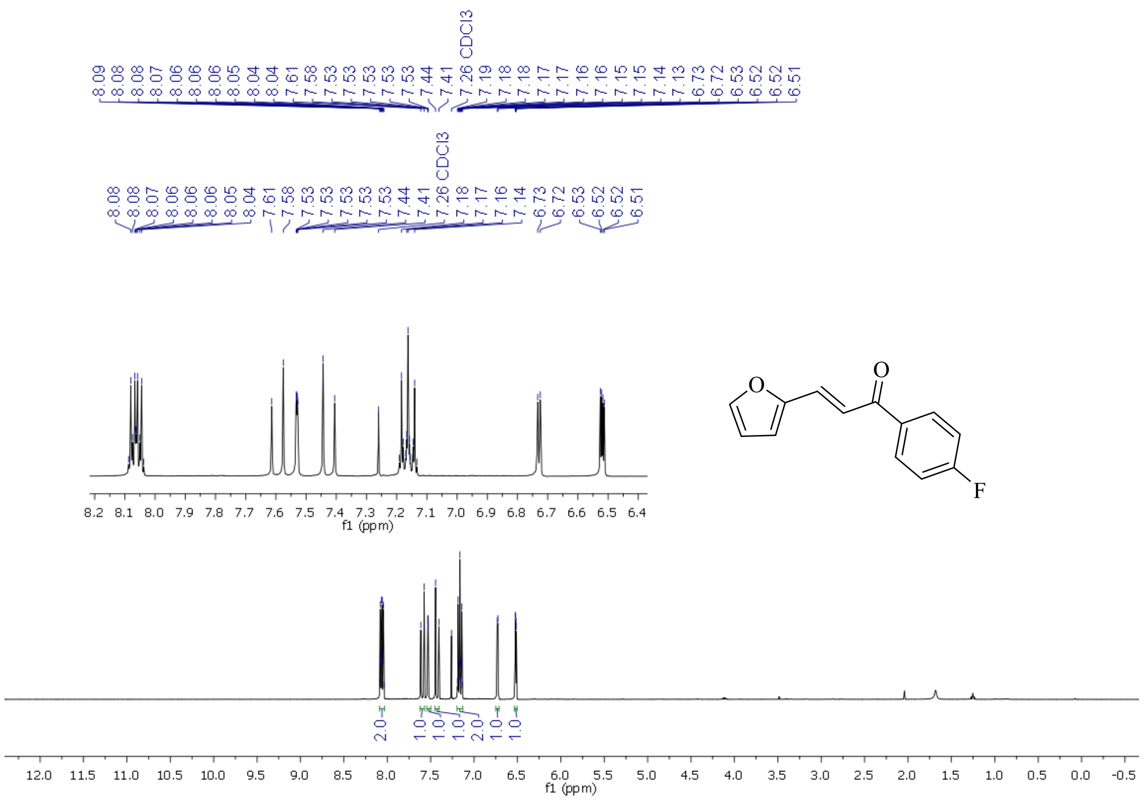

Figure S8. ^{1}H NMR (400 MHz, $CDCl_3$, ppm) of (*E*)-1-(4-fluorophenyl)-3-(furan-2-yl)prop-2-en-1-one (3b).

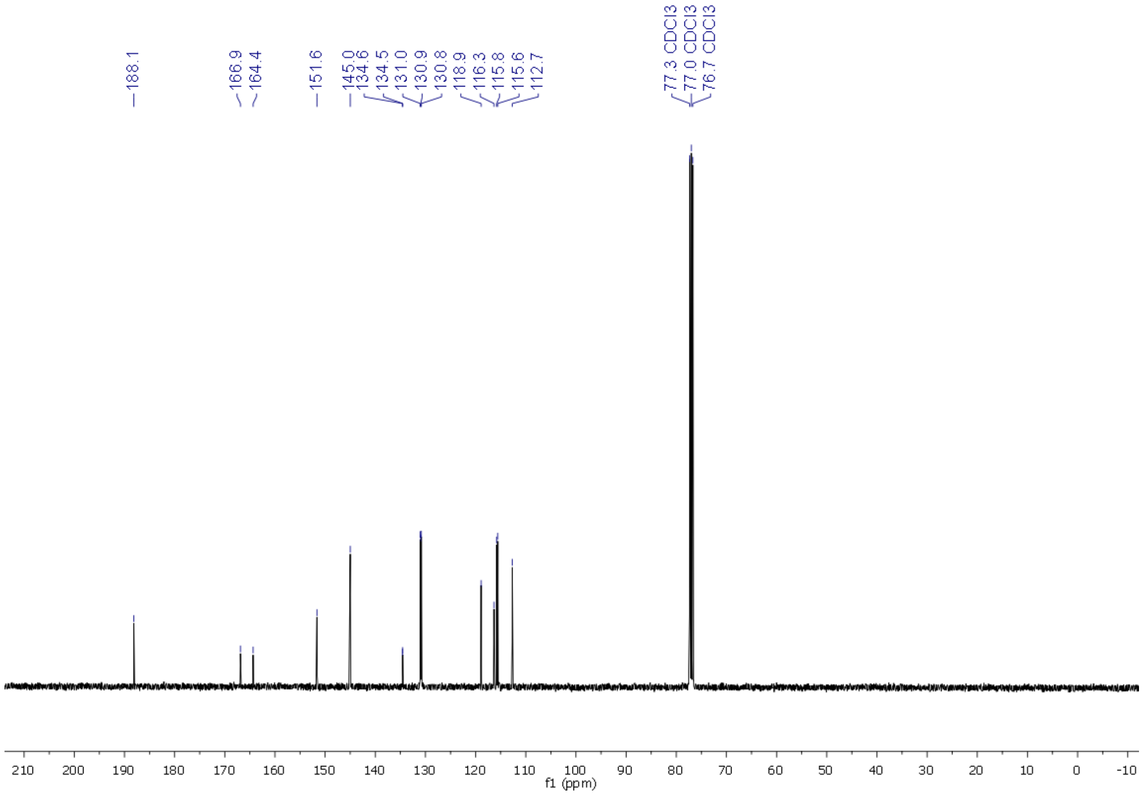

Figure S9. ^{13}C NMR (101 MHz, $CDCl_3$, ppm) of (*E*)-1-(4-fluorophenyl)-3-(furan-2-yl)prop-2-en-1-one (3b).

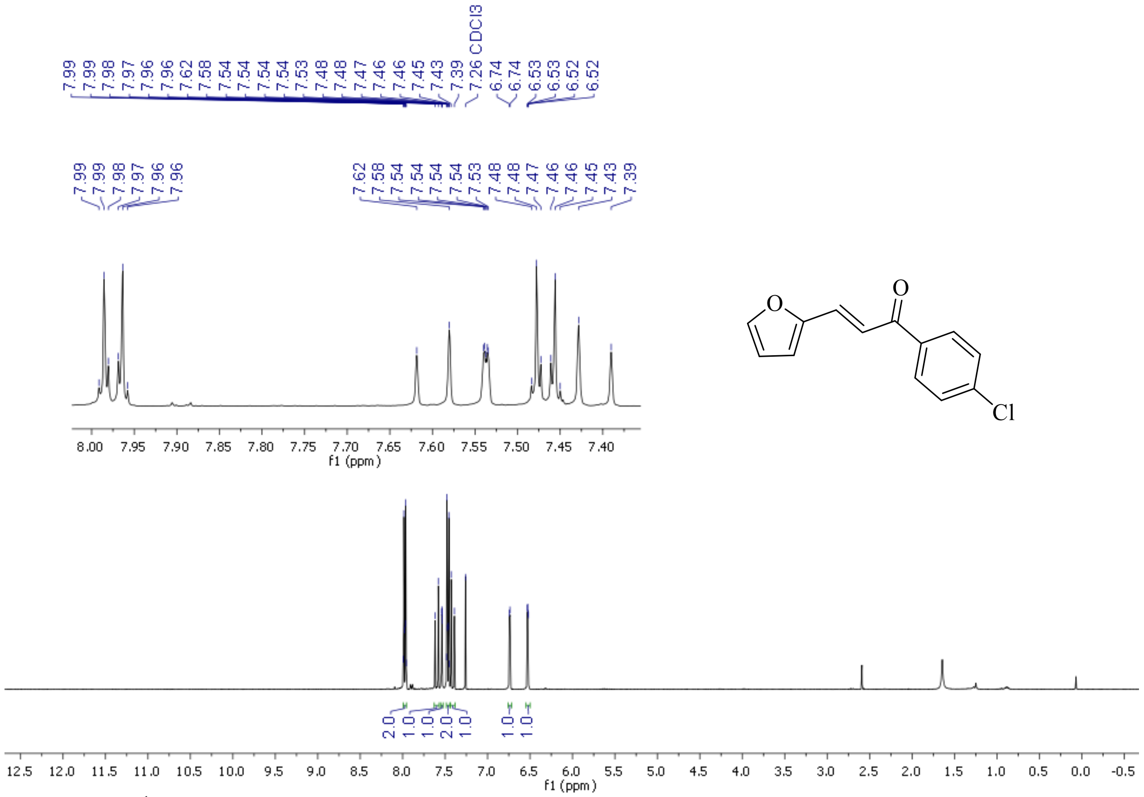

Figure S10. ^{1}H NMR (400 MHz, $CDCl_3$, ppm) of (*E*)-1-(4-chlorophenyl)-3-(furan-2-yl)prop-2-en-1-one (3c).

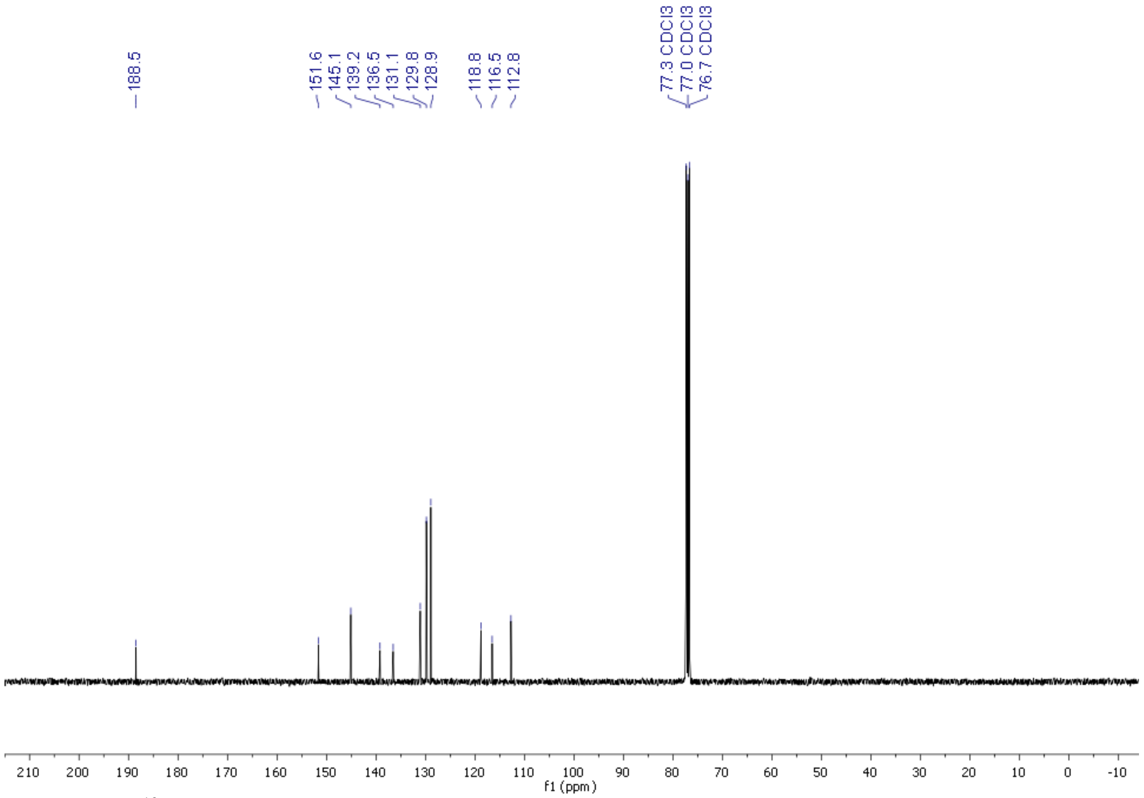

Figure S11. ^{13}C NMR (101 MHz, $CDCl_3$, ppm) of (*E*)-1-(4-chlorophenyl)-3-(furan-2-yl)prop-2-en-1-one (3c).

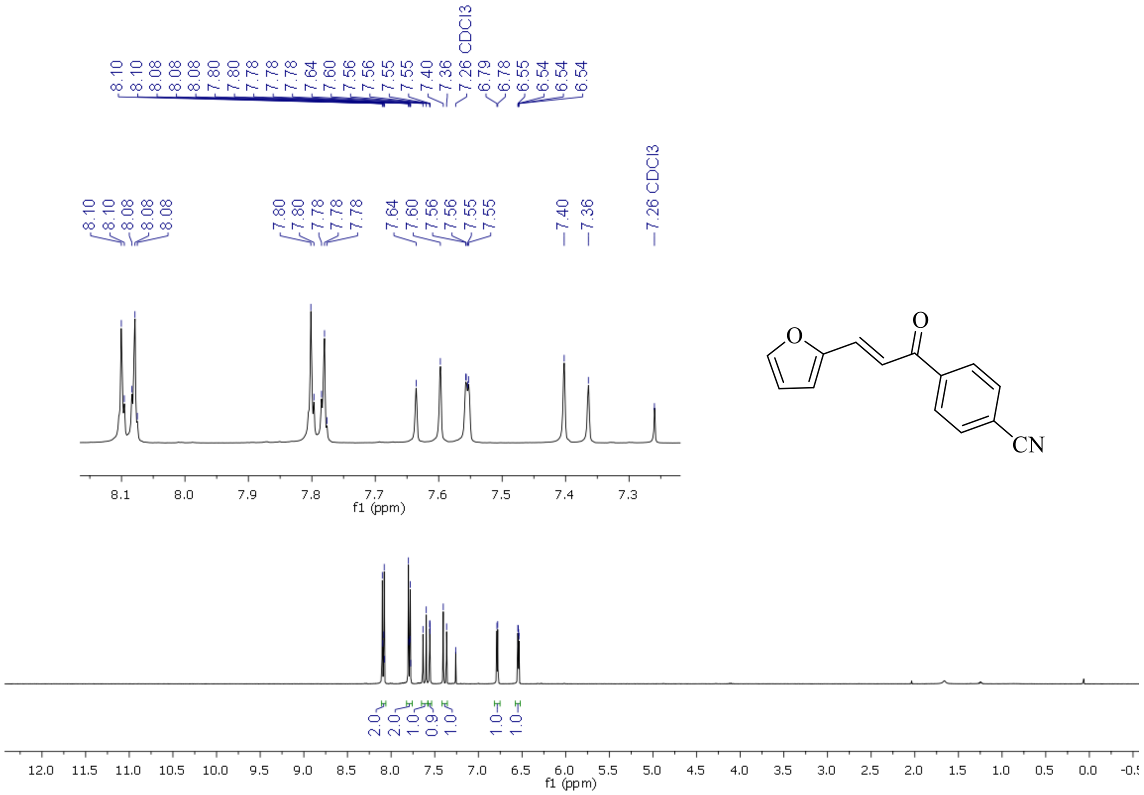

Figure S12. ^{1}H NMR (400 MHz, $CDCl_3$, ppm) of (*E*)-4-(3-(furan-2-yl)acryloyl)benzonitrile (3d).

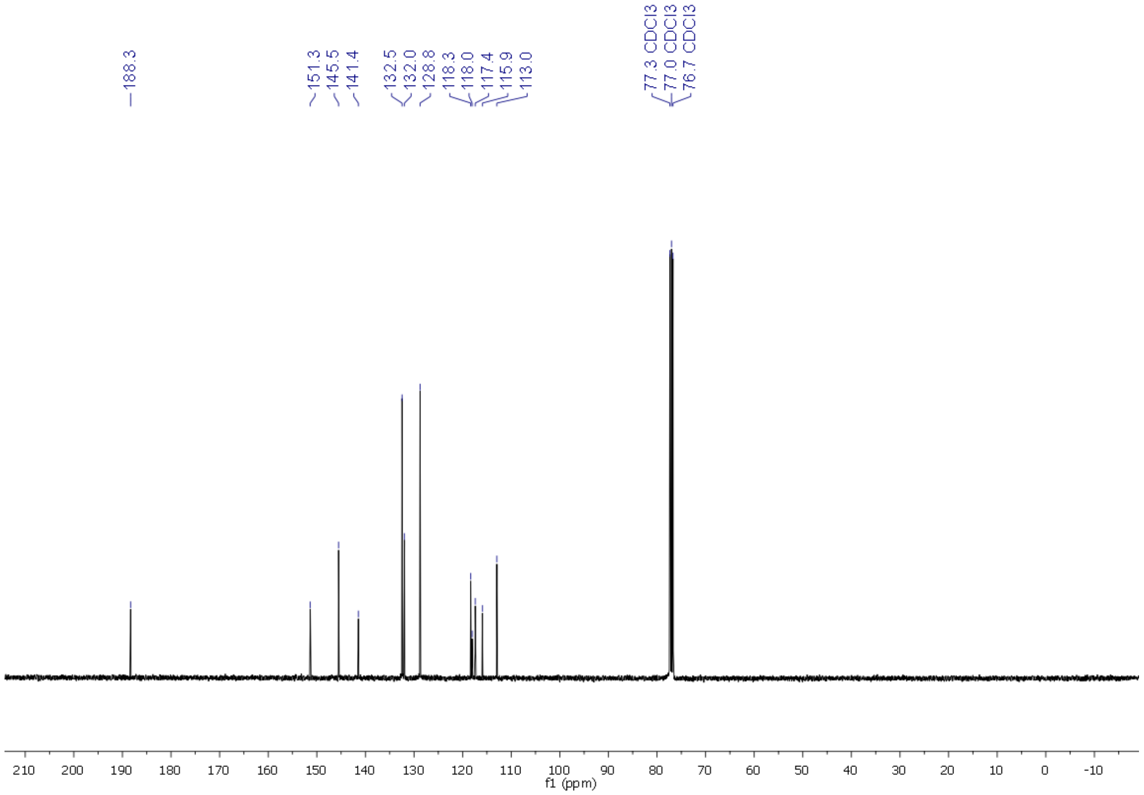

Figure S13. ^{13}C NMR (101 MHz, $CDCl_3$, ppm) of (*E*)-4-(3-(furan-2-yl)acryloyl)benzonitrile (3d).

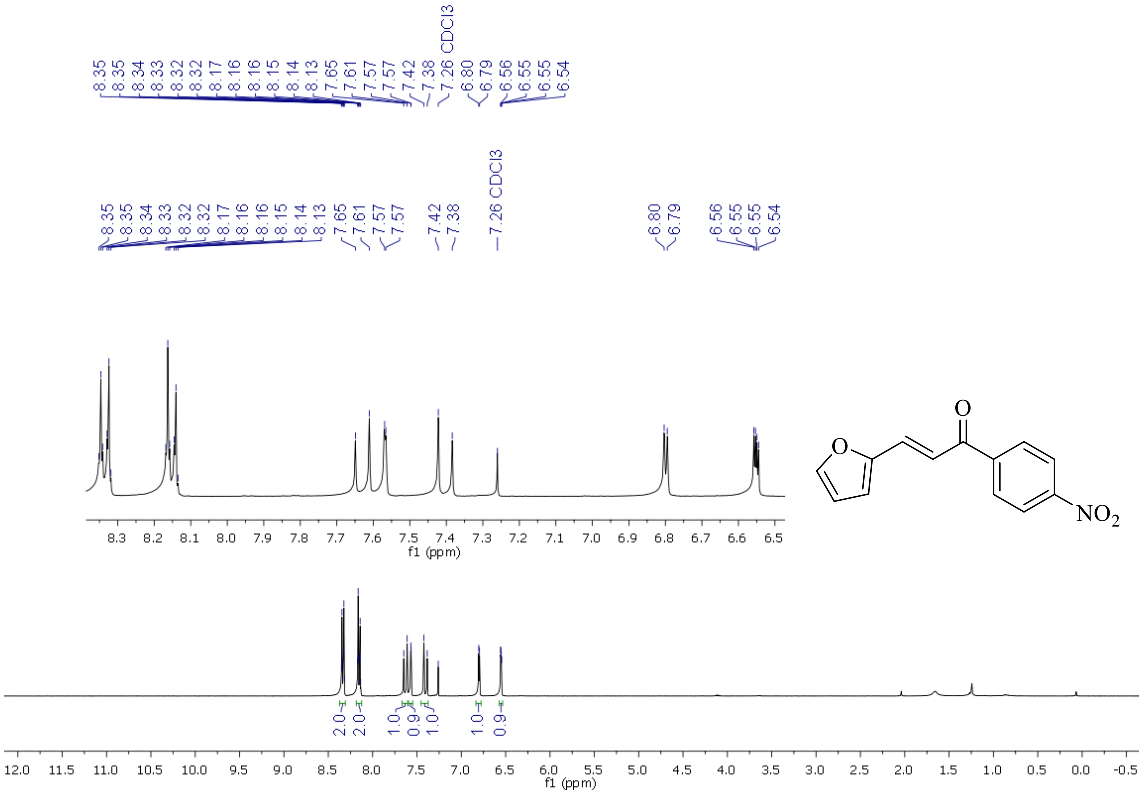

Figure S14. ^{1}H NMR (400 MHz, $CDCl_3$, ppm) of (*E*)-3-(furan-2-yl)-1-(4-nitrophenyl)prop-2-en-1-one (3e).

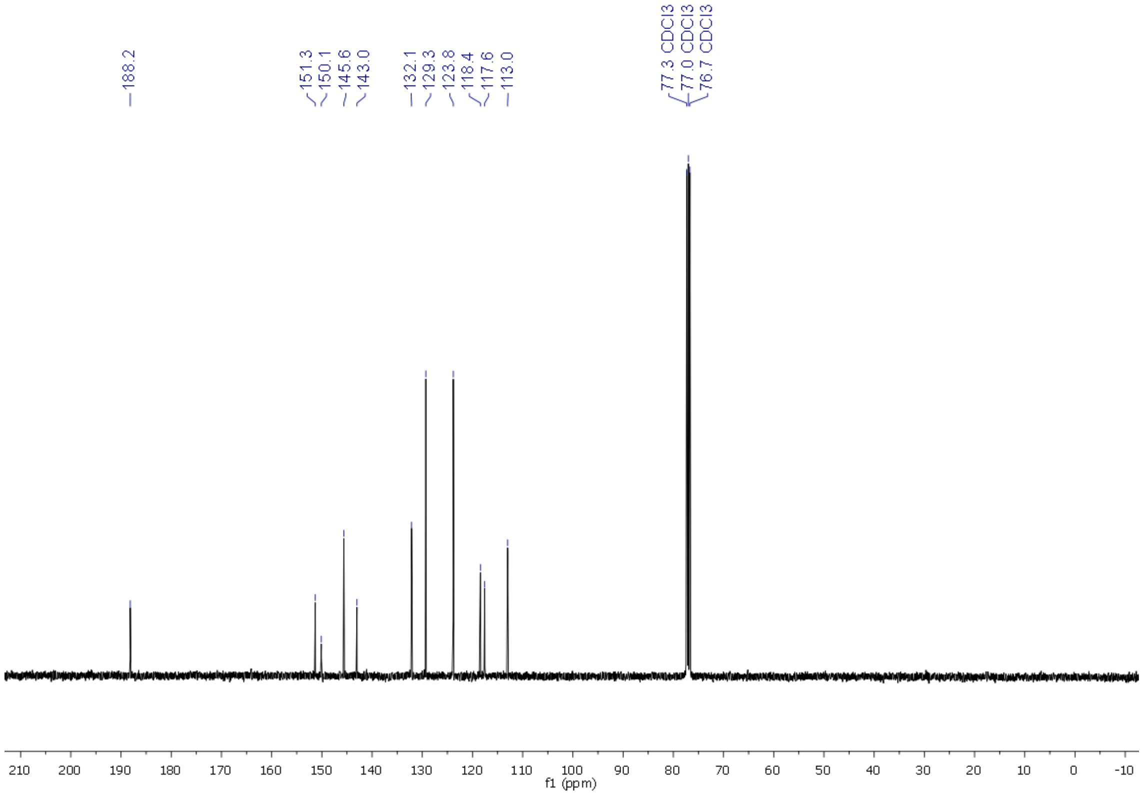

Figure S15. ^{13}C NMR (101 MHz, $CDCl_3$, ppm) of (*E*)-3-(furan-2-yl)-1-(4-nitrophenyl)prop-2-en-1-one (3e).

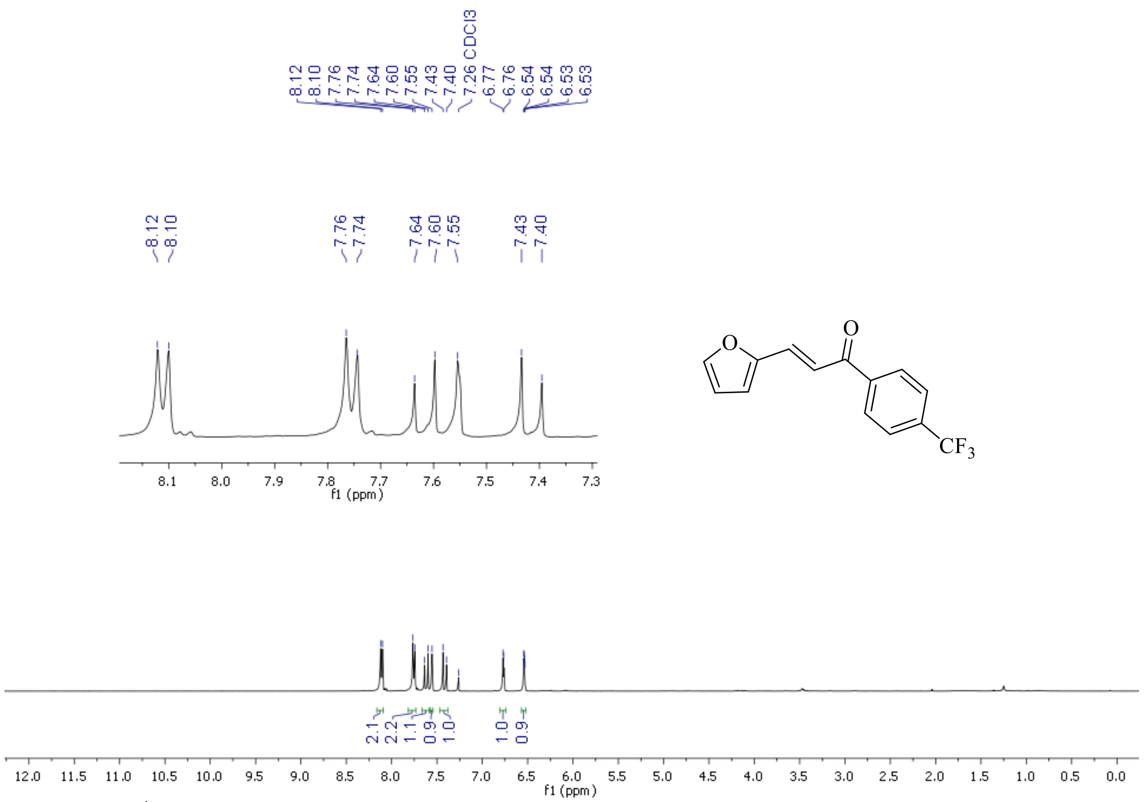

Figure S16. ^{1}H NMR (400 MHz, $CDCl_3$, ppm) of (*E*)-3-(furan-2-yl)-1-(4-(trifluoromethyl)phenyl)prop-2-en-1-one (3f).

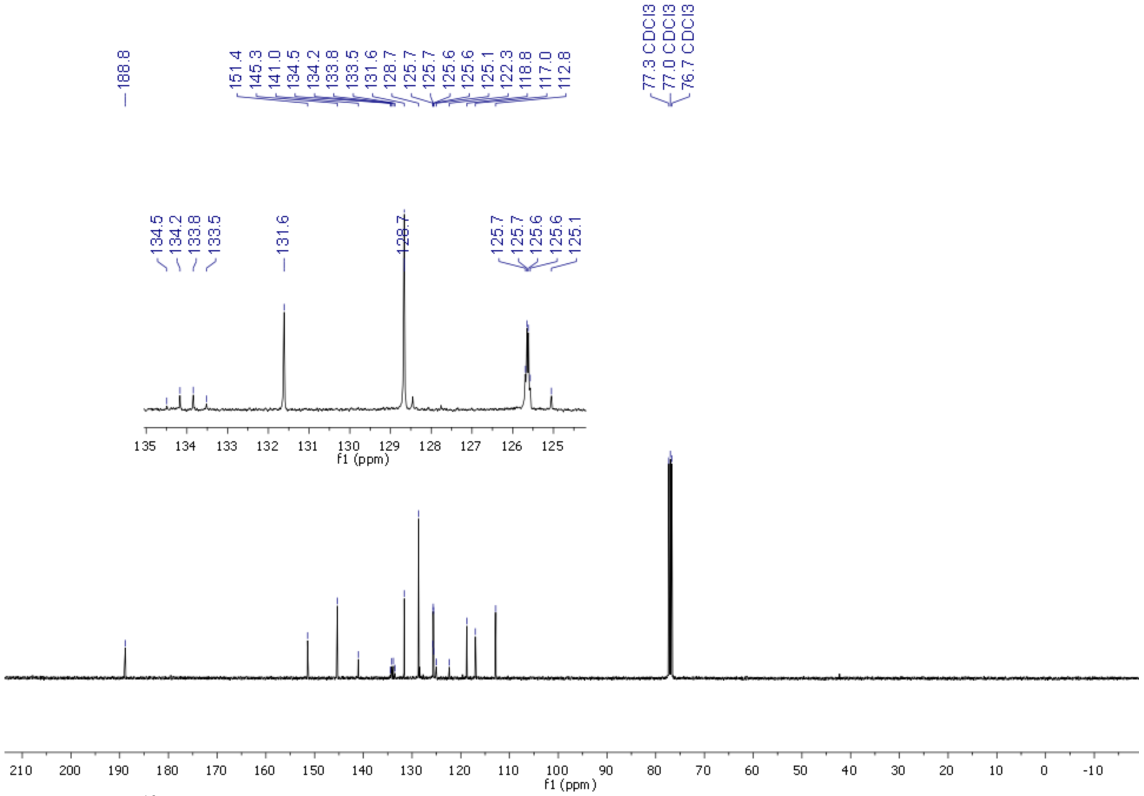

Figure S17. ^{13}C NMR (101 MHz, $CDCl_3$, ppm) of (*E*)-3-(furan-2-yl)-1-(4-(trifluoromethyl)phenyl)prop-2-en-1-one (3f).

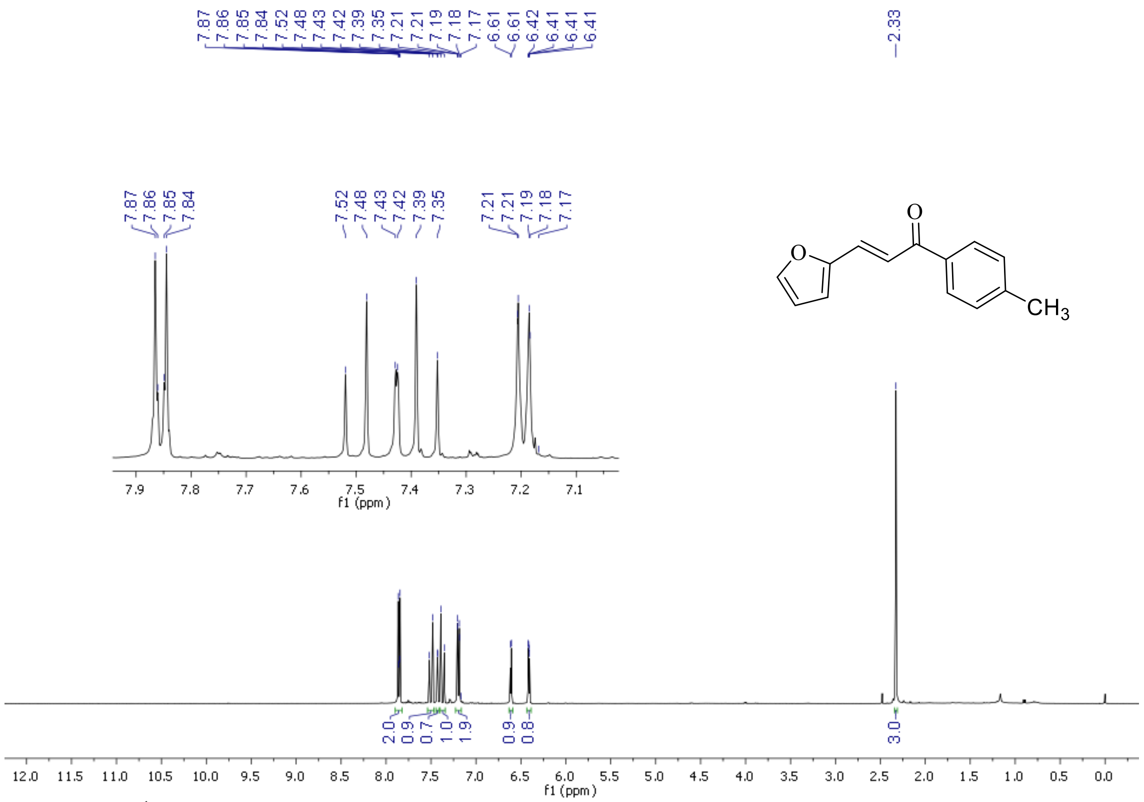

Figure S18. ^{1}H NMR (400 MHz, $CDCl_3$, ppm) of (*E*)-3-(furan-2-yl)-1-(p-tolyl)prop-2-en-1-one (3g).

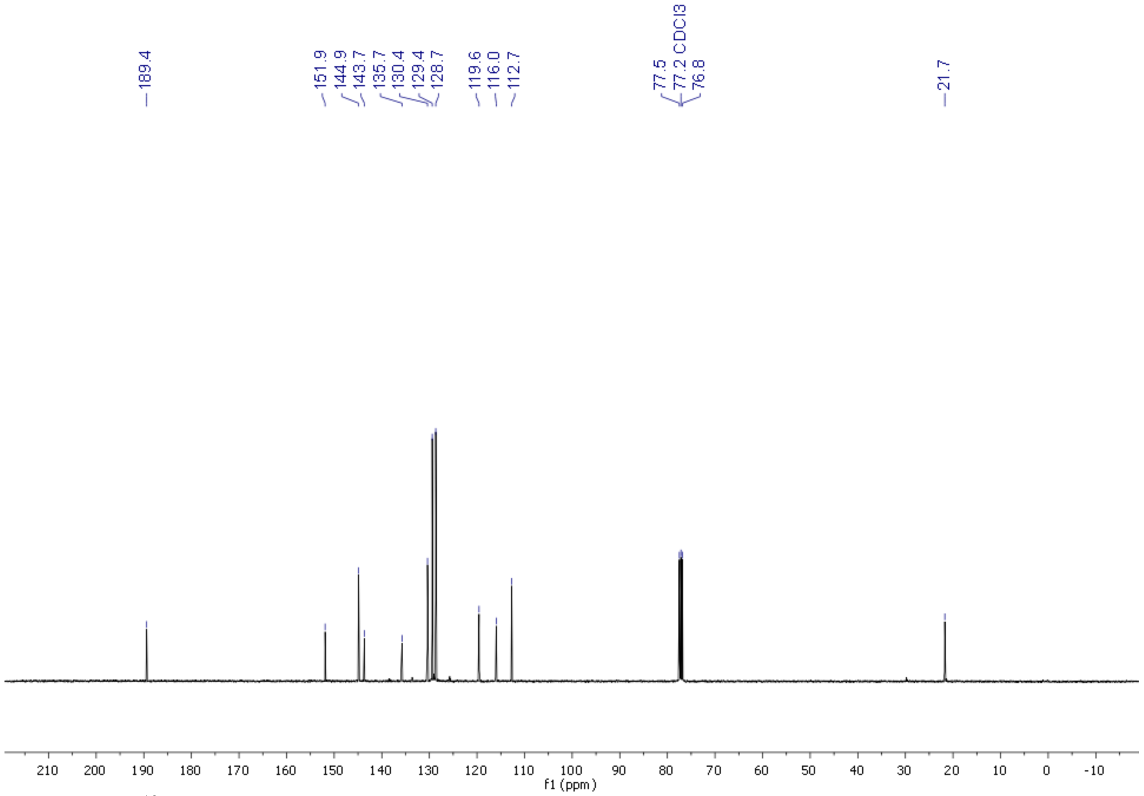

Figure S19. ^{13}C NMR (101 MHz, $CDCl_3$, ppm) of (*E*)-3-(furan-2-yl)-1-(p-tolyl)prop-2-en-1-one (3g).

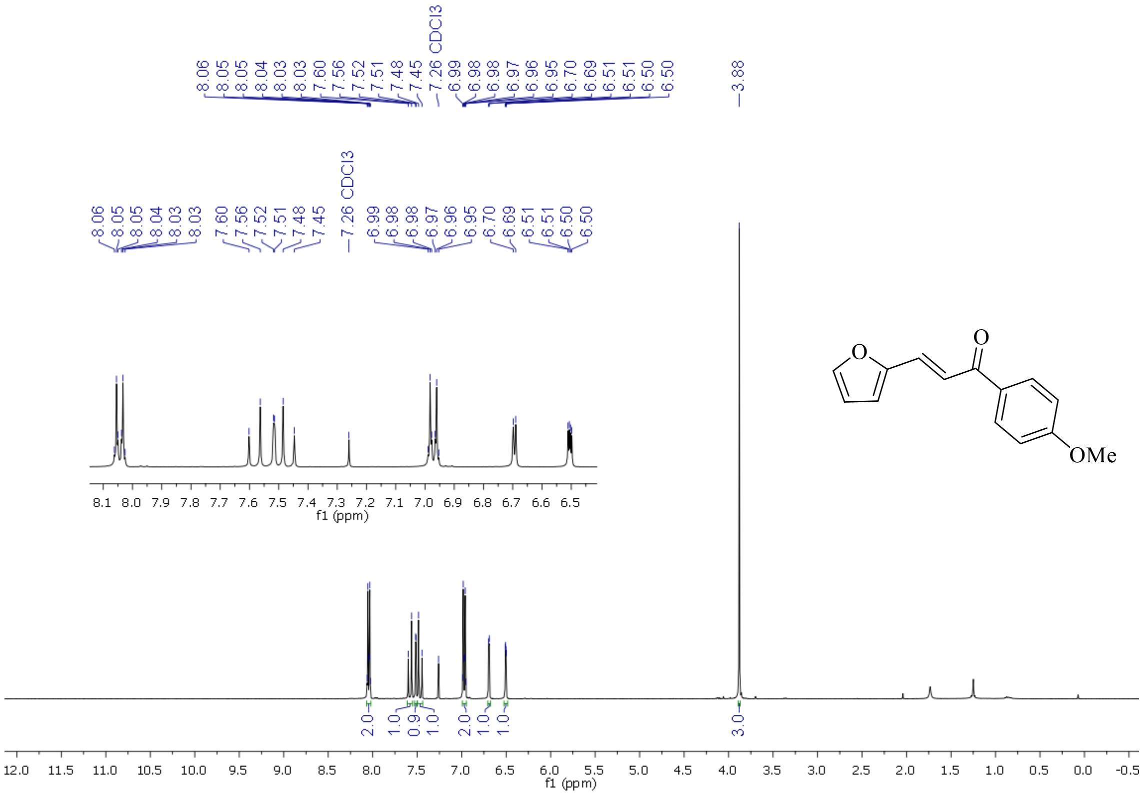

Figure S20. ^{1}H NMR (400 MHz, $CDCl_3$, ppm) of (*E*)-3-(furan-2-yl)-1-(4-methoxyphenyl)prop-2-en-1-one (3h).

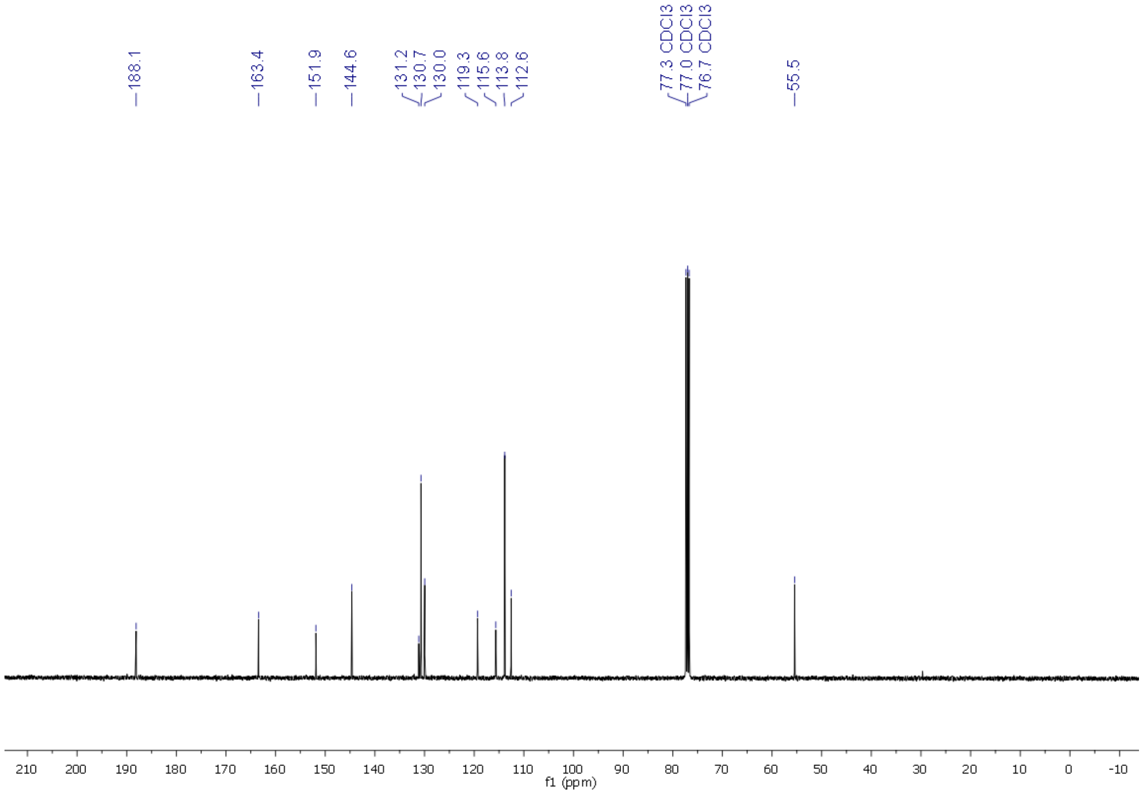

Figure S21. ^{13}C NMR (101 MHz, $CDCl_3$, ppm) of (*E*)-3-(furan-2-yl)-1-(4-methoxyphenyl)prop-2-en-1-one (3h).

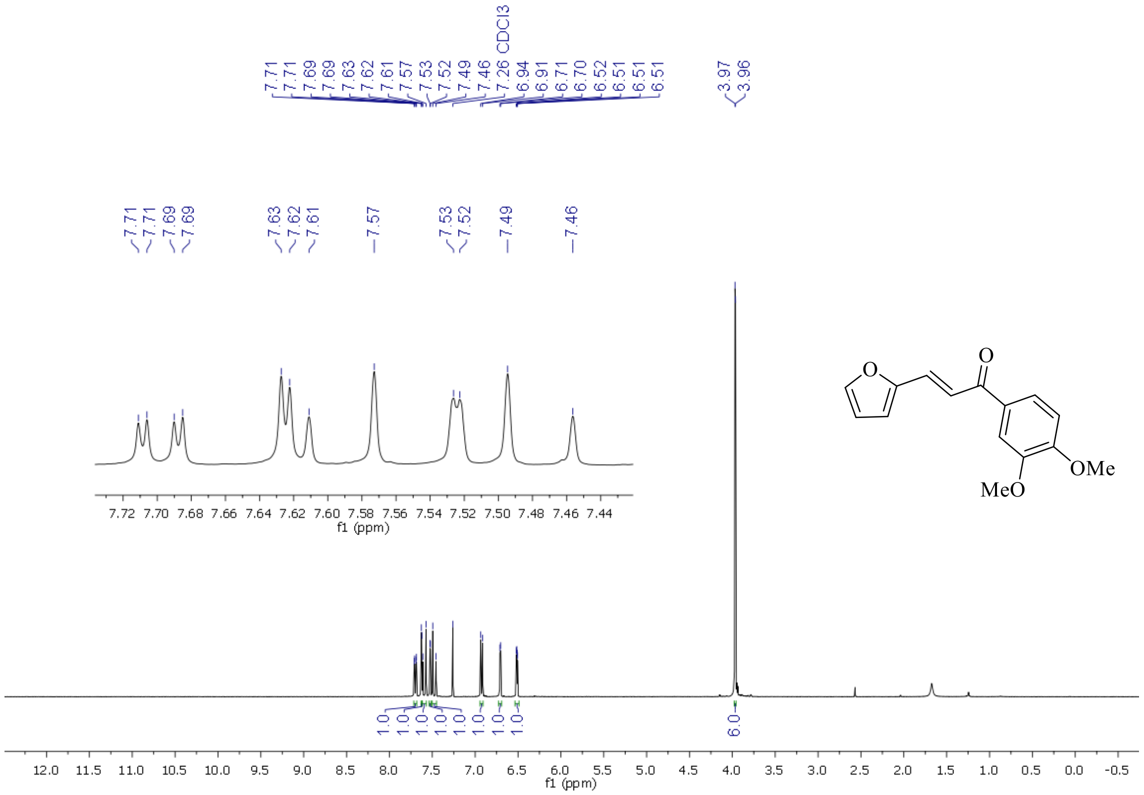

Figure S22. ^{1}H NMR (400 MHz, $CDCl_3$, ppm) of (*E*)-1-(3,4-dimethoxyphenyl)-3-(furan-2-yl)prop-2-en-1-one (3i).

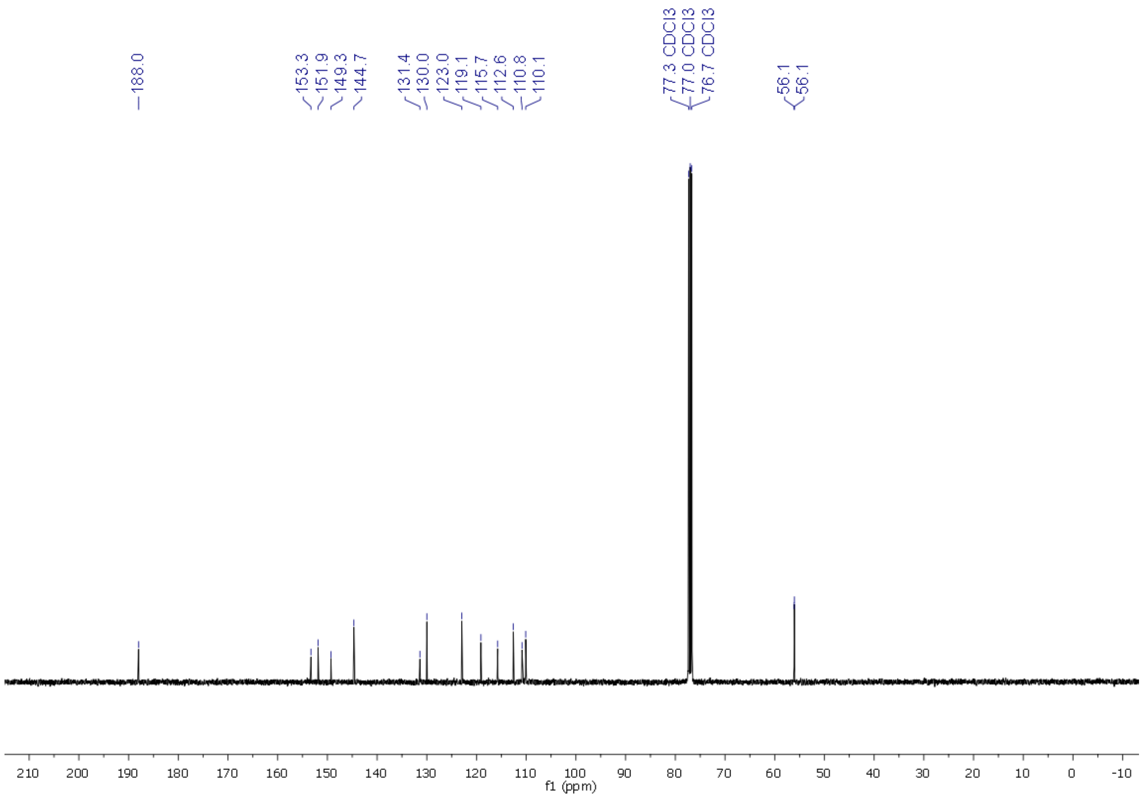

Figure S23. ^{13}C NMR (101 MHz, $CDCl_3$, ppm) of (*E*)-1-(3,4-dimethoxyphenyl)-3-(furan-2-yl)prop-2-en-1-one (3i).

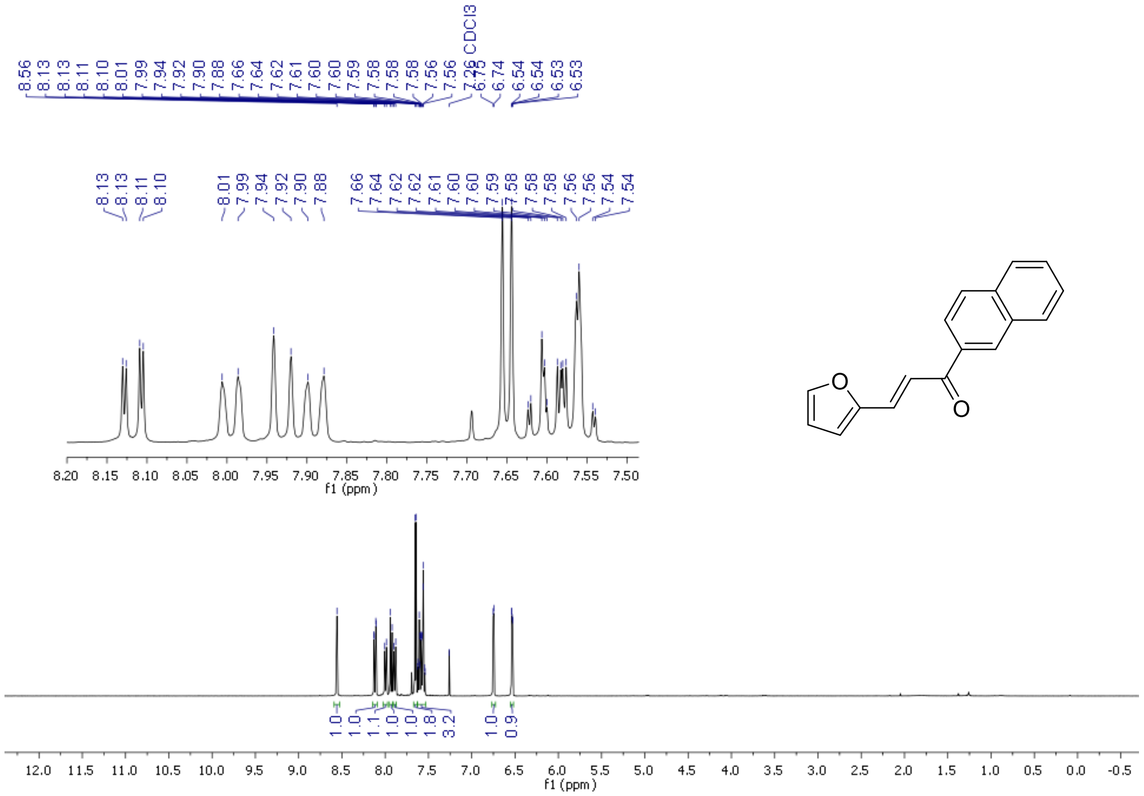

Figure S24. ^{1}H NMR (400 MHz, $CDCl_3$, ppm) of (*E*)-3-(furan-2-yl)-1-(naphthalen-2-yl)prop-2-en-1-one (3j).

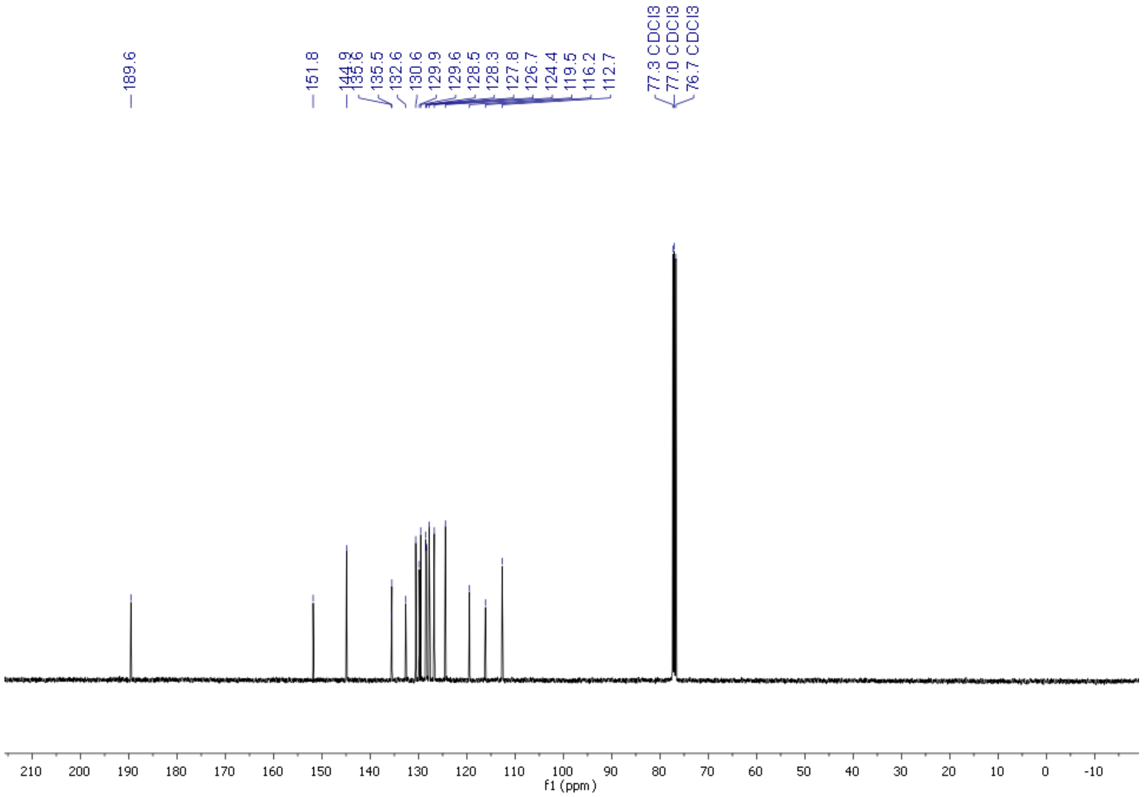

Figure S25. ^{13}C NMR (101 MHz, $CDCl_3$, ppm) of (*E*)-3-(furan-2-yl)-1-(naphthalen-2-yl)prop-2-en-1-one (3j).

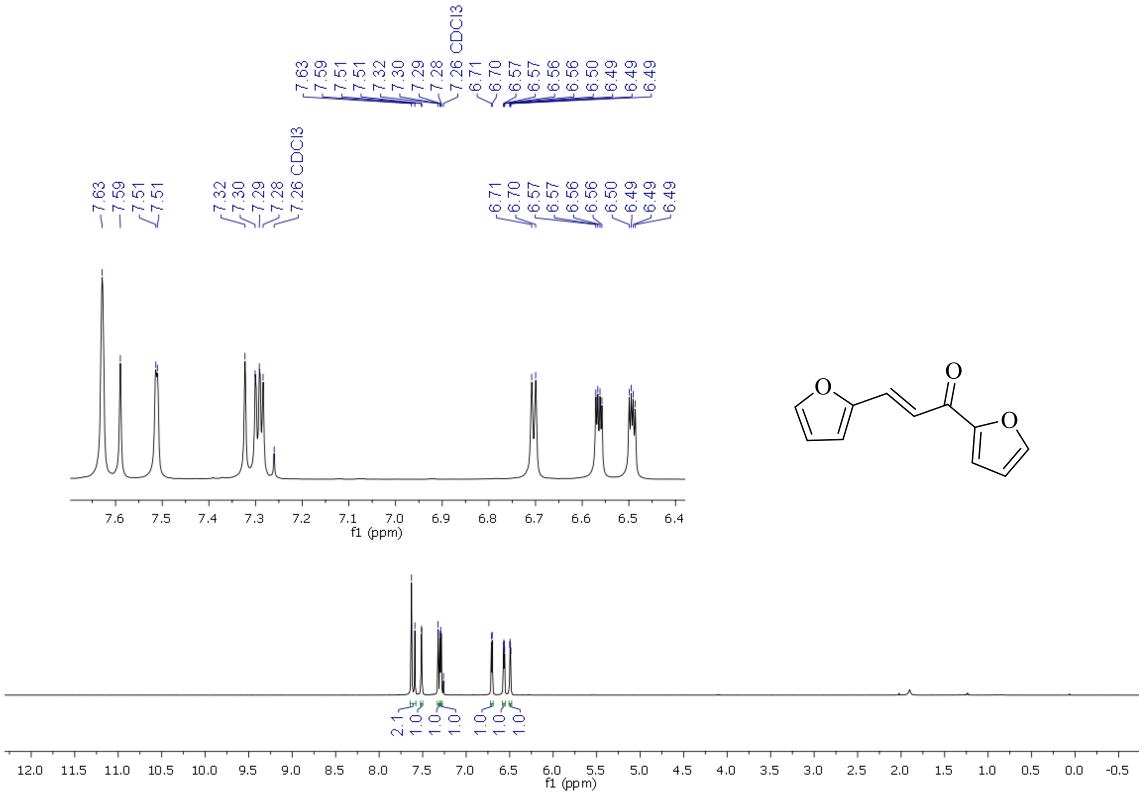

Figure S26. ^{1}H NMR (400 MHz, $CDCl_3$, ppm) of (*E*)-1,3-di(furan-2-yl)prop-2-en-1-one (3k).

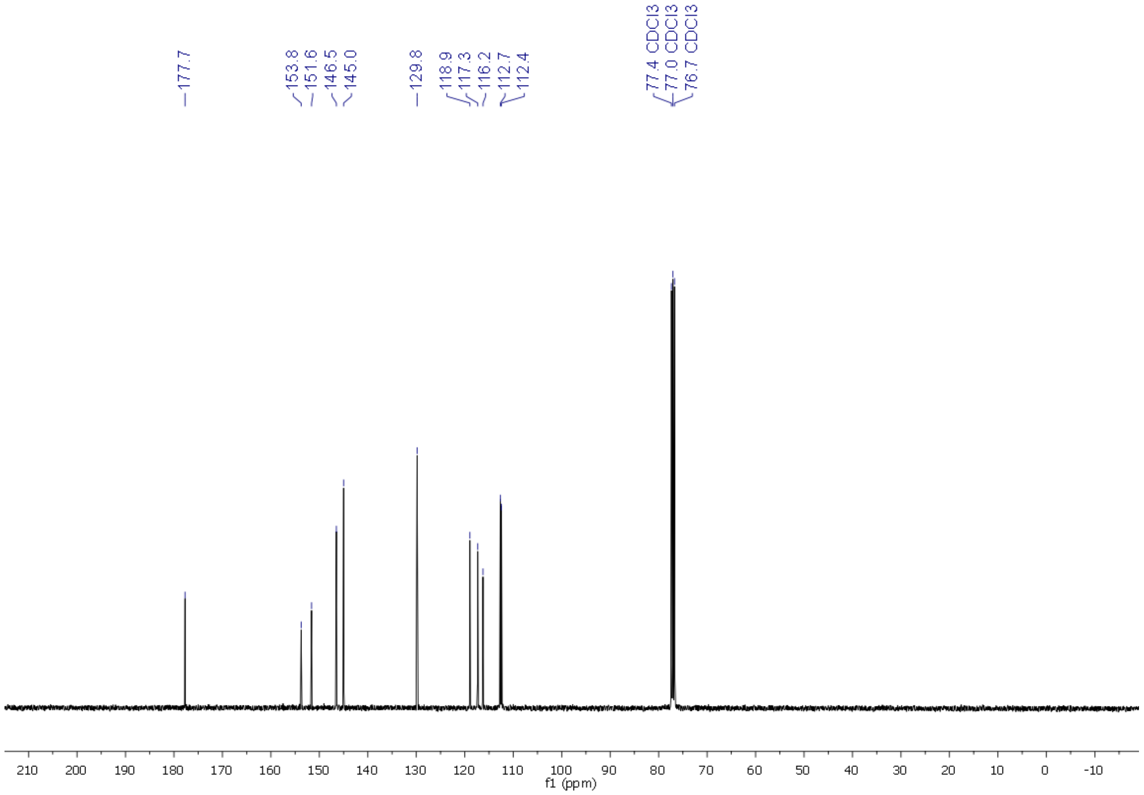

Figure S27. ^{13}C NMR (101 MHz, $CDCl_3$, ppm) of (*E*)-1,3-di(furan-2-yl)prop-2-en-1-one (3k).

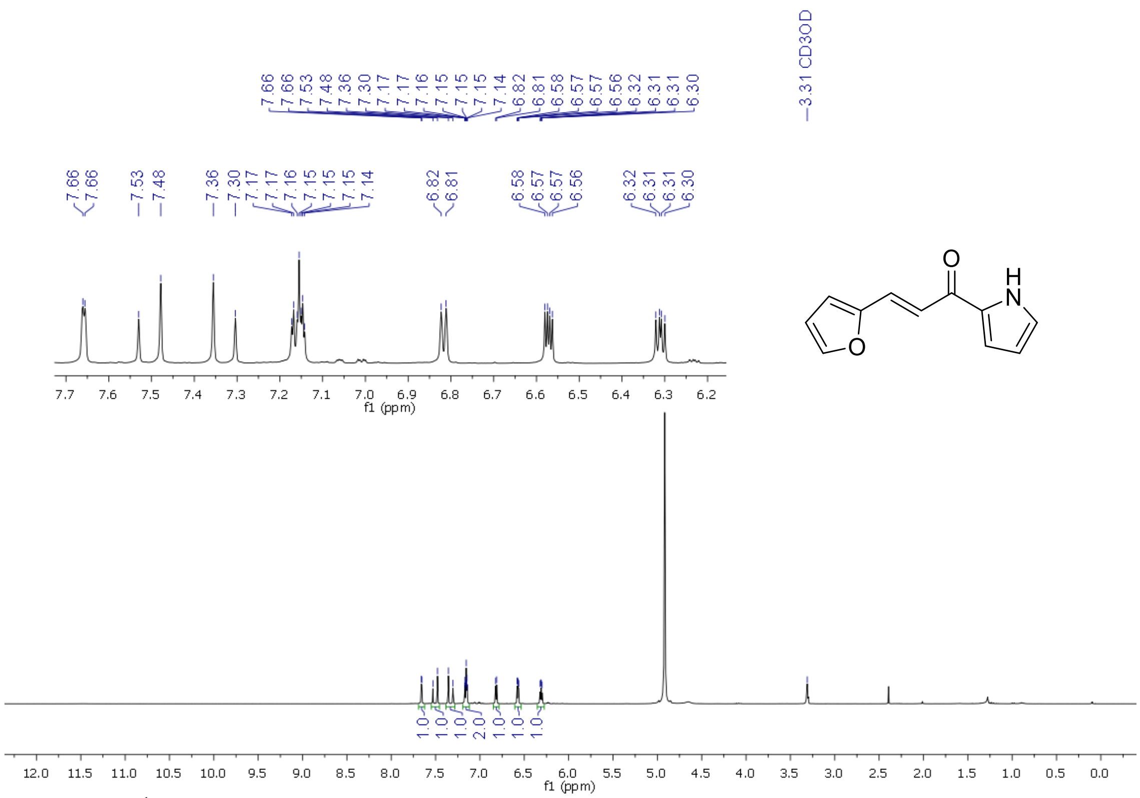

Figure S28. ^{1}H NMR (500 MHz, CD_3OD, ppm) of (*E*)-3-(furan-2-yl)-1-(1H-pyrrol-2-yl)prop-2-en-1-one (3l).

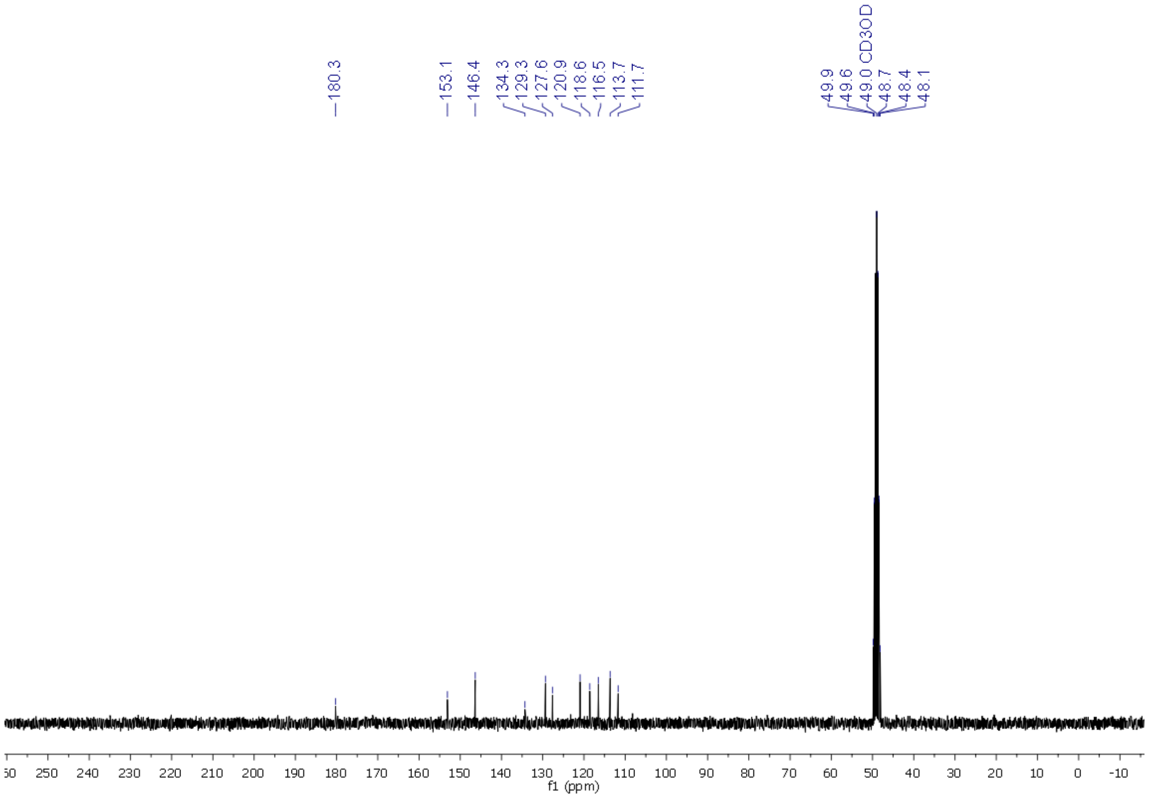

Figure S29. ^{13}C NMR (125 MHz, CD_3OD, ppm) of (*E*)-3-(furan-2-yl)-1-(1H-pyrrol-2-yl)prop-2-en-1-one (3l).

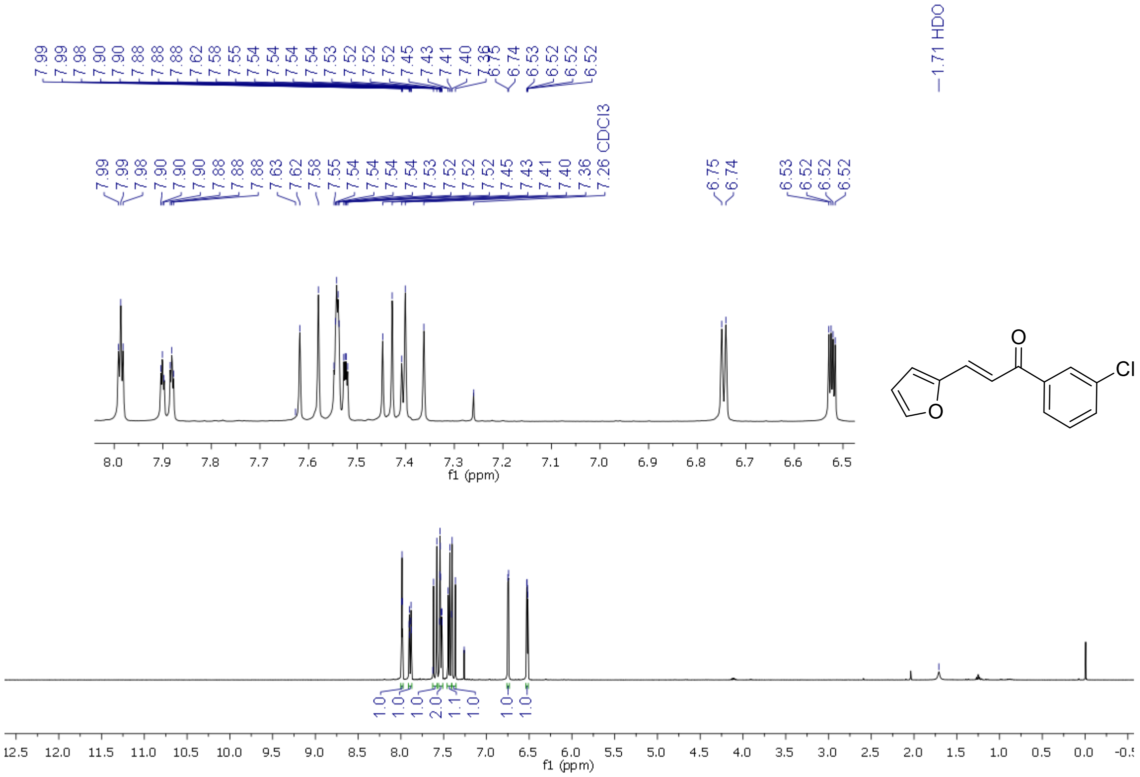

Figure S30. ^{1}H NMR (400 MHz, $CDCl_3$, ppm) of (*E*)-1-(3-chlorophenyl)-3-(furan-2-yl)prop-2-en-1-one (3m).

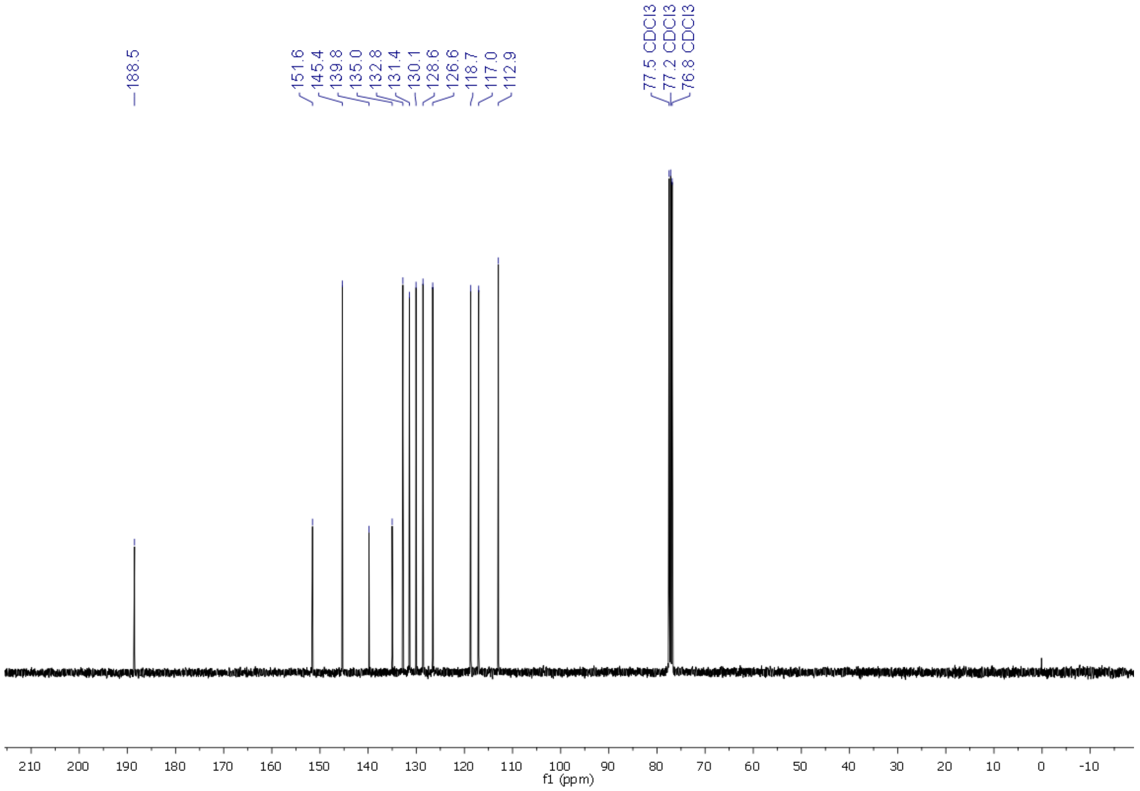

Figure S31. ^{13}C NMR (101 MHz, $CDCl_3$, ppm) of (*E*)-1-(3-chlorophenyl)-3-(furan-2-yl)prop-2-en-1-one (3m).

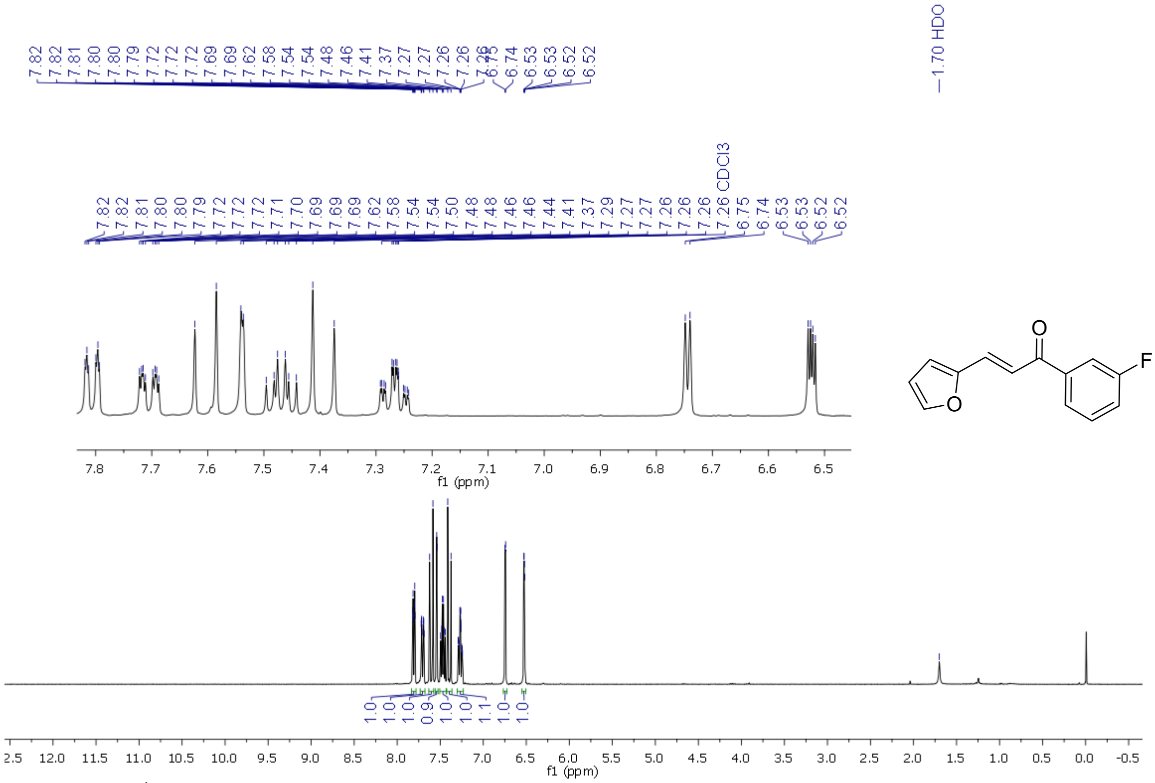

Figure S32. ^{1}H NMR (400 MHz, $CDCl_3$, ppm) of (*E*)-1-(3-fluorophenyl)-3-(furan-2-yl)prop-2-en-1-one (3n).

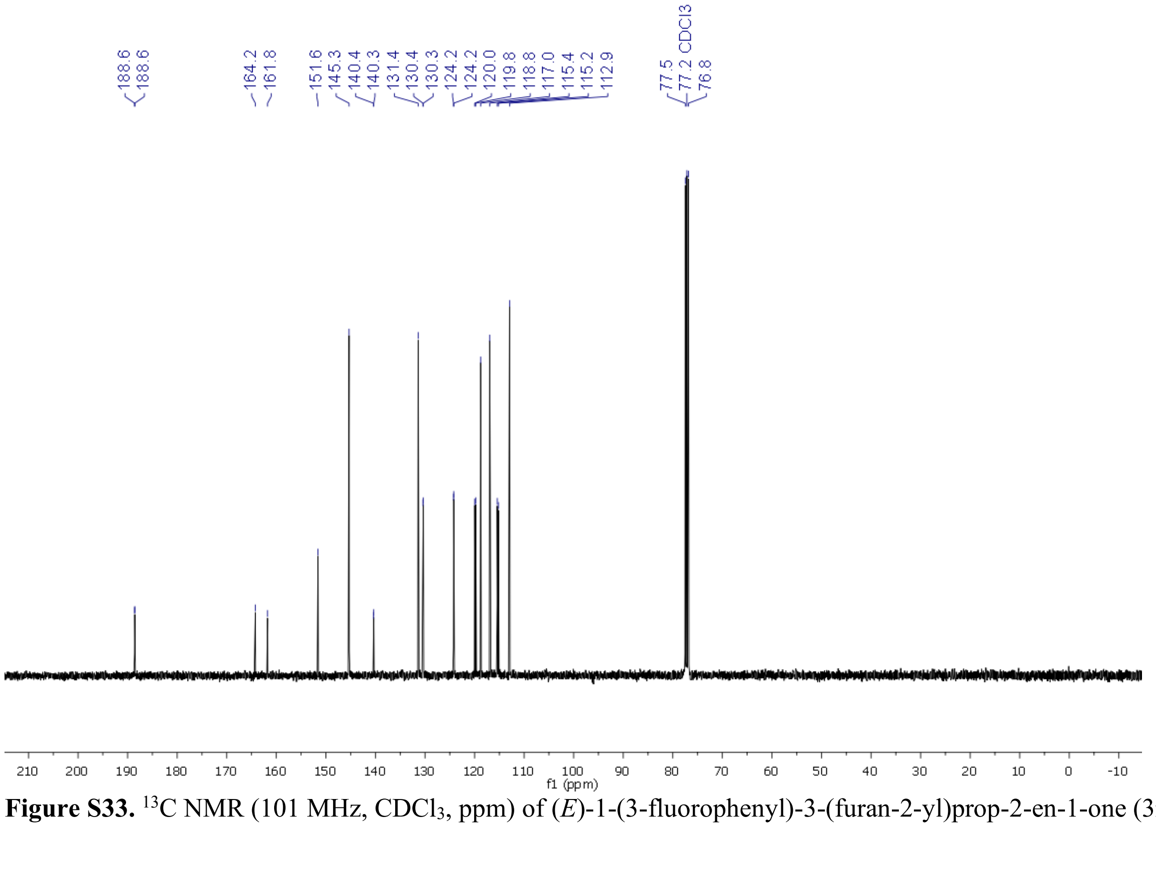

Figure S33. ^{13}C NMR (101 MHz, $CDCl_3$, ppm) of (*E*)-1-(3-fluorophenyl)-3-(furan-2-yl)prop-2-en-1-one (3n).

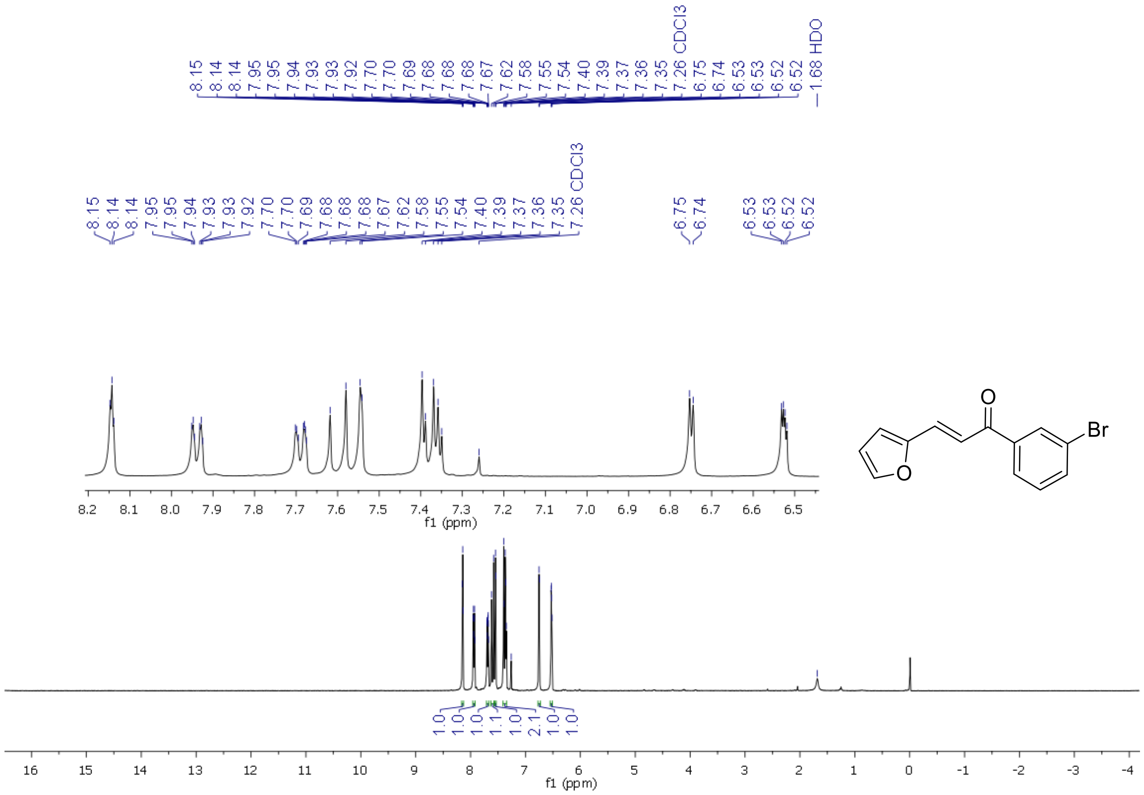

Figure S34. ^{1}H NMR (400 MHz, $CDCl_3$, ppm) of (*E*)-1-(3-bromophenyl)-3-(furan-2-yl)prop-2-en-1-one (3o).

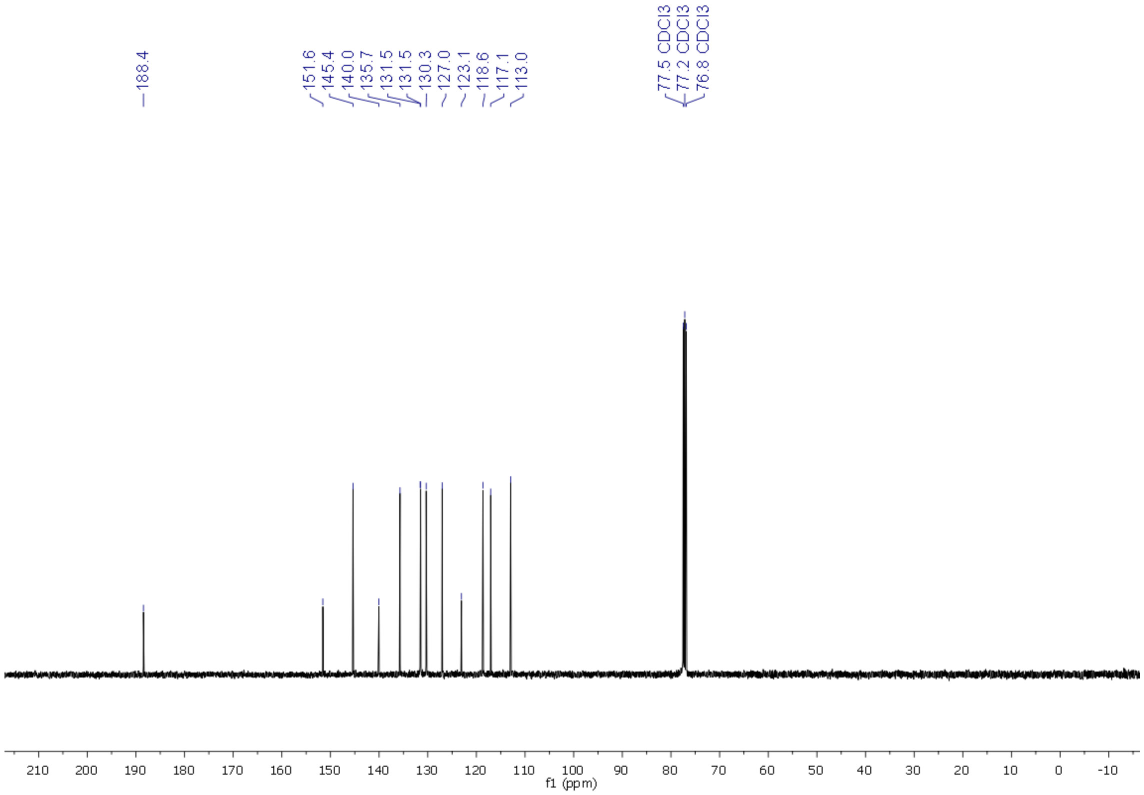

Figure S35. ^{13}C NMR (101 MHz, $CDCl_3$, ppm) of (*E*)-1-(3-bromophenyl)-3-(furan-2-yl)prop-2-en-1-one (3o).

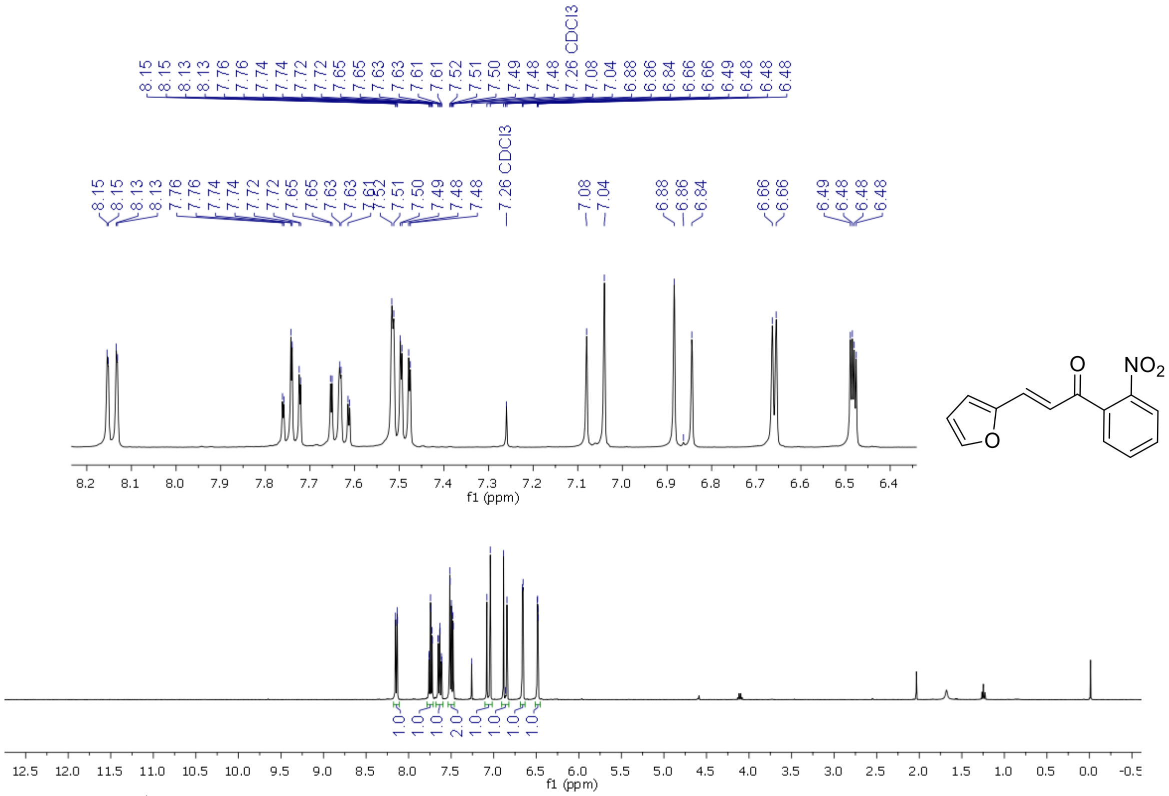

Figure S36. ^{1}H NMR (400 MHz, $CDCl_3$, ppm) of (*E*)-3-(furan-2-yl)-1-(2-nitrophenyl)prop-2-en-1-one (3p).

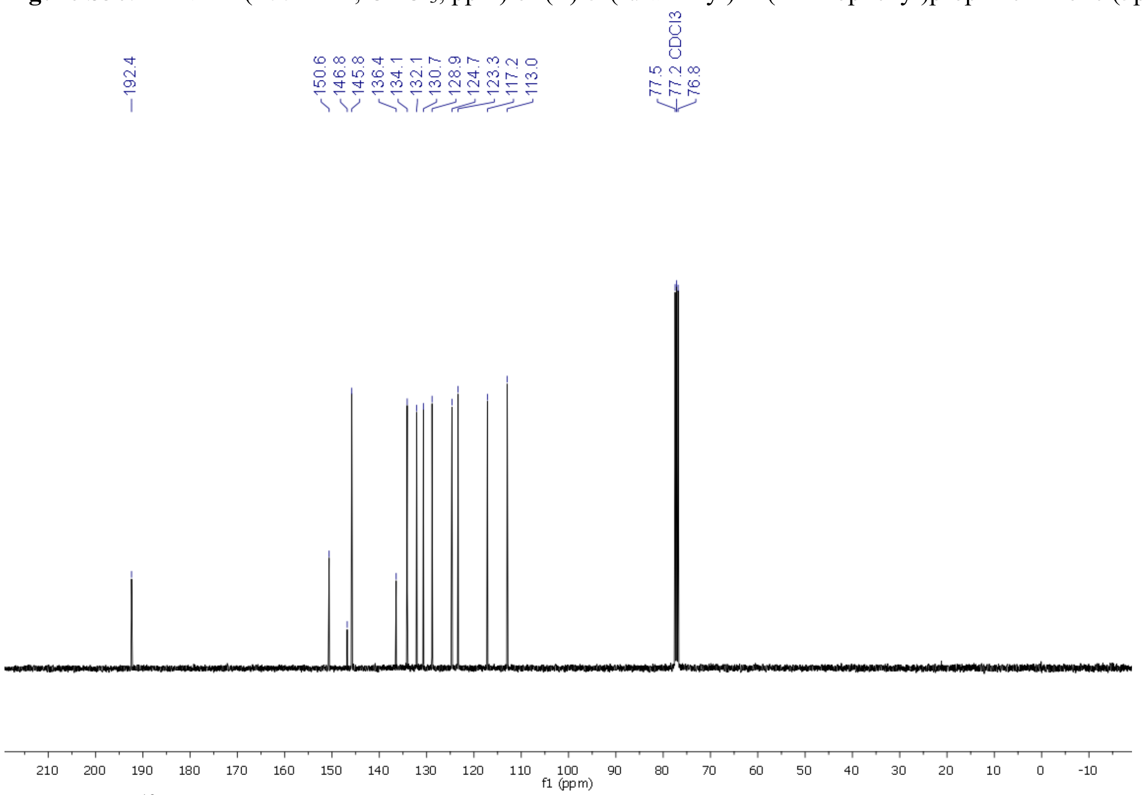

Figure S37. ^{13}C NMR (101 MHz, $CDCl_3$, ppm) of (*E*)-3-(furan-2-yl)-1-(2-nitrophenyl)prop-2-en-1-one (3p).

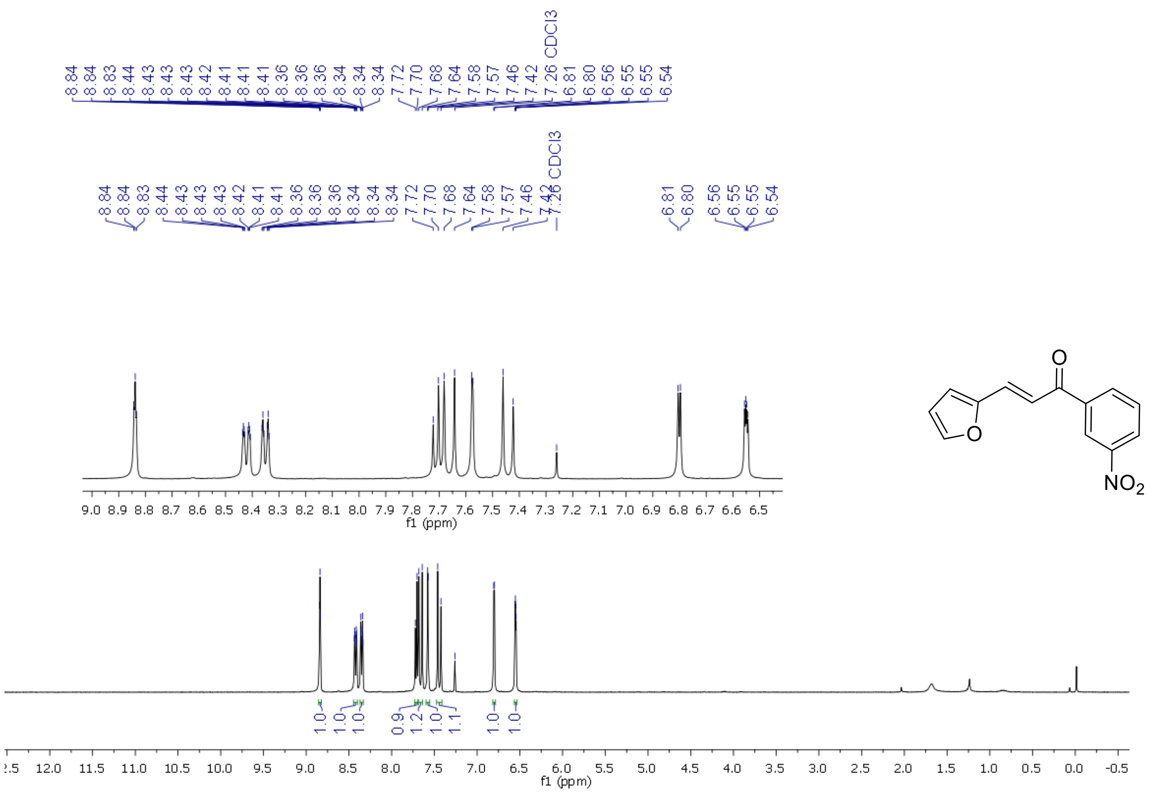

Figure S38. ^{1}H NMR (400 MHz, $CDCl_3$, ppm) of (*E*)-3-(furan-2-yl)-1-(3-nitrophenyl)prop-2-en-1-one (3q).

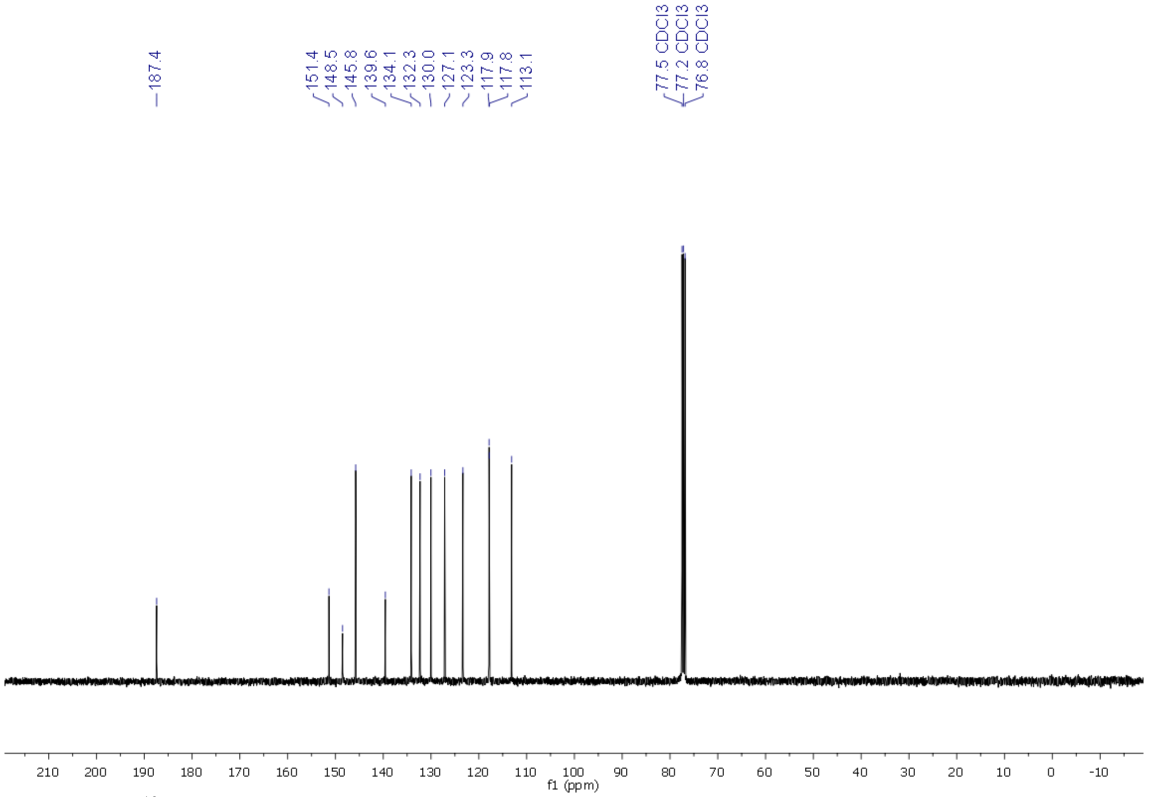

Figure S39. ^{13}C NMR (101 MHz, $CDCl_3$, ppm) of (*E*)-3-(furan-2-yl)-1-(3-nitrophenyl)prop-2-en-1-one (3q).

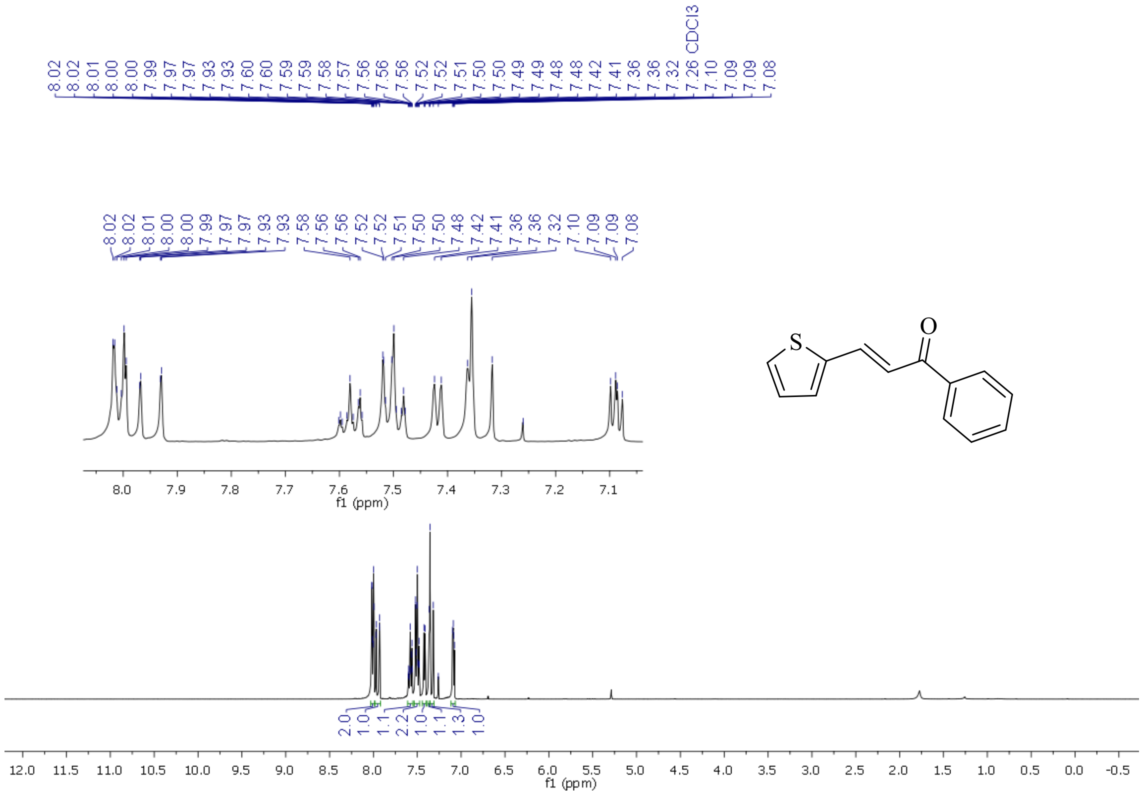

Figure S40. ^{1}H NMR (400 MHz, $CDCl_3$, ppm) of (*E*)-1-phenyl-3-(thiophen-2-yl)prop-2-en-1-one (5a).

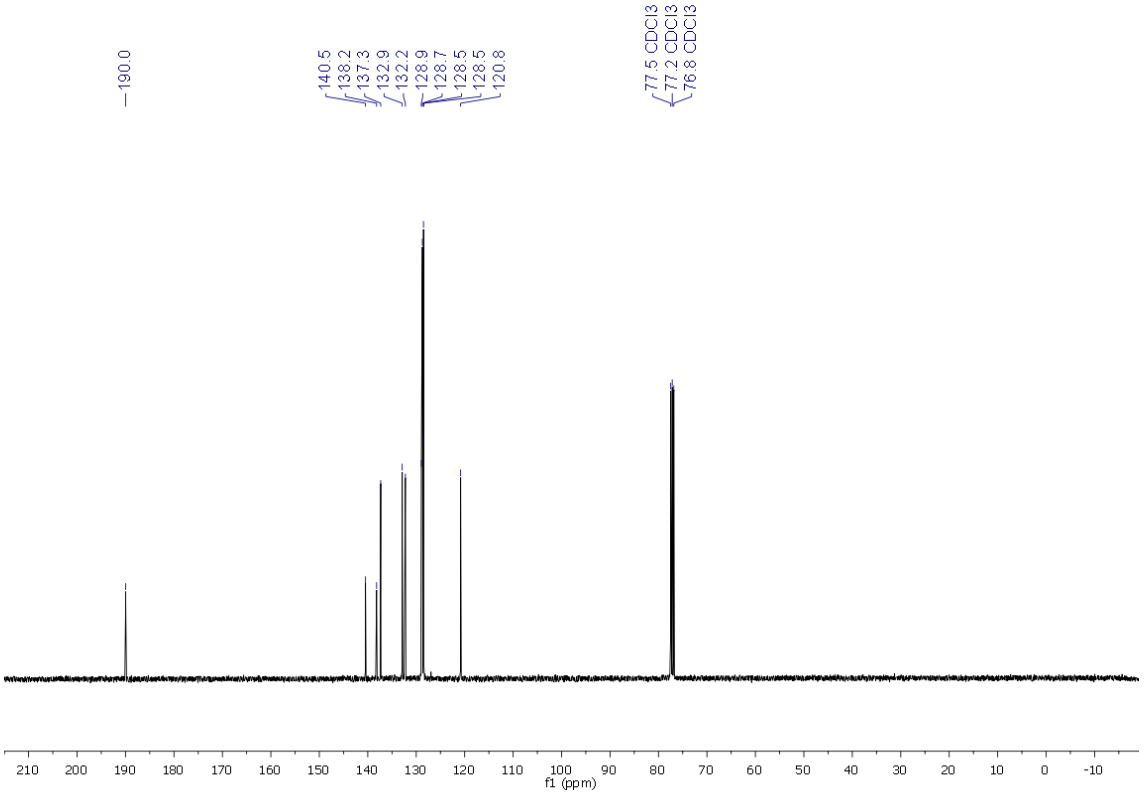

Figure S41. ^{13}C NMR (101 MHz, $CDCl_3$, ppm) of (*E*)-1-phenyl-3-(thiophen-2-yl)prop-2-en-1-one (5a).

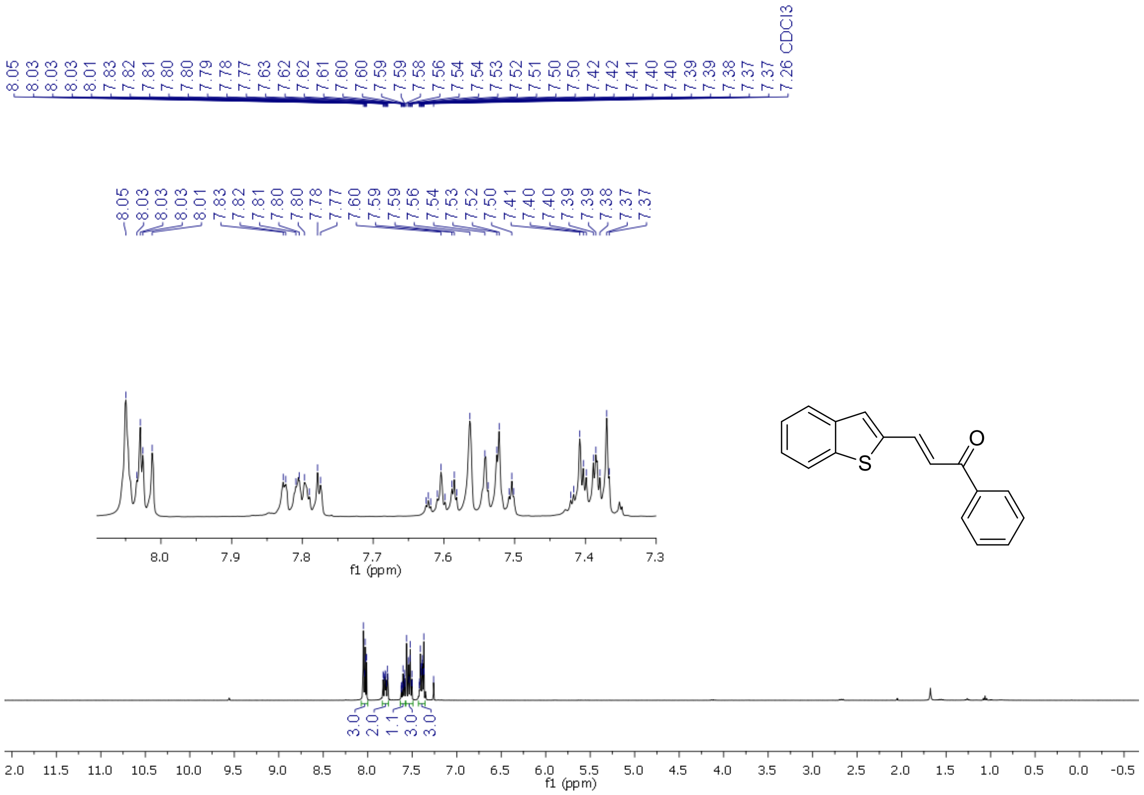

Figure S42. ^{1}H NMR (400 MHz, $CDCl_3$, ppm) of (*E*)-3-(benzo[b]thiophen-2-yl)-1-phenylprop-2-en-1-one (5b).

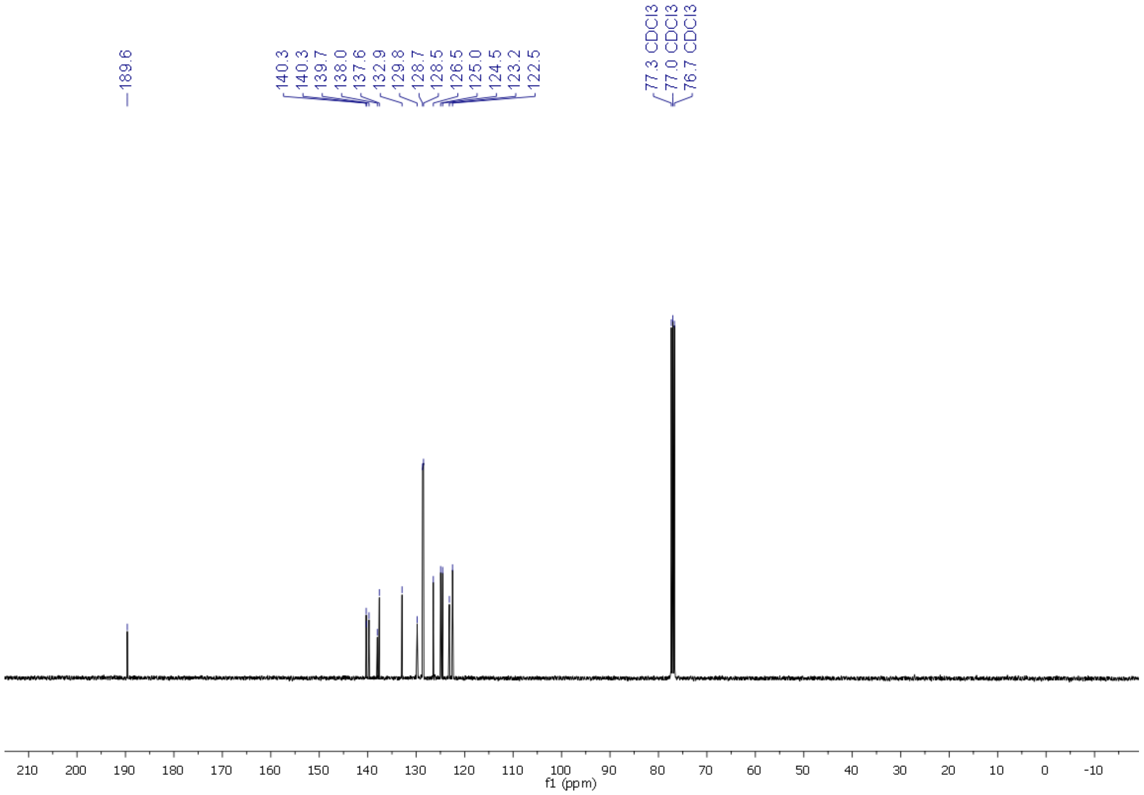

Figure S43. ^{13}C NMR (101 MHz, $CDCl_3$, ppm) of (*E*)-3-(benzo[b]thiophen-2-yl)-1-phenylprop-2-en-1-one (5b).

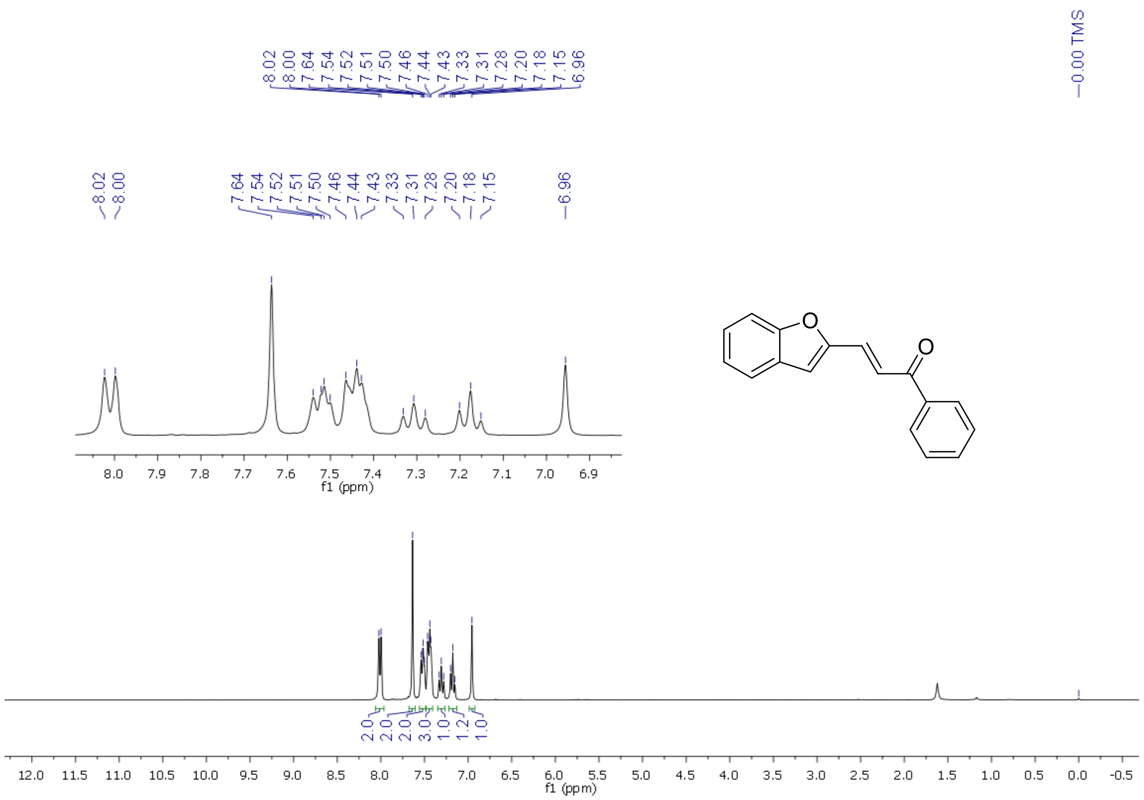

Figure S44. ^{1}H NMR (300 MHz, $CDCl_3$, ppm) of (*E*)-3-(benzofuran-2-yl)-1-phenylprop-2-en-1-one (5c).

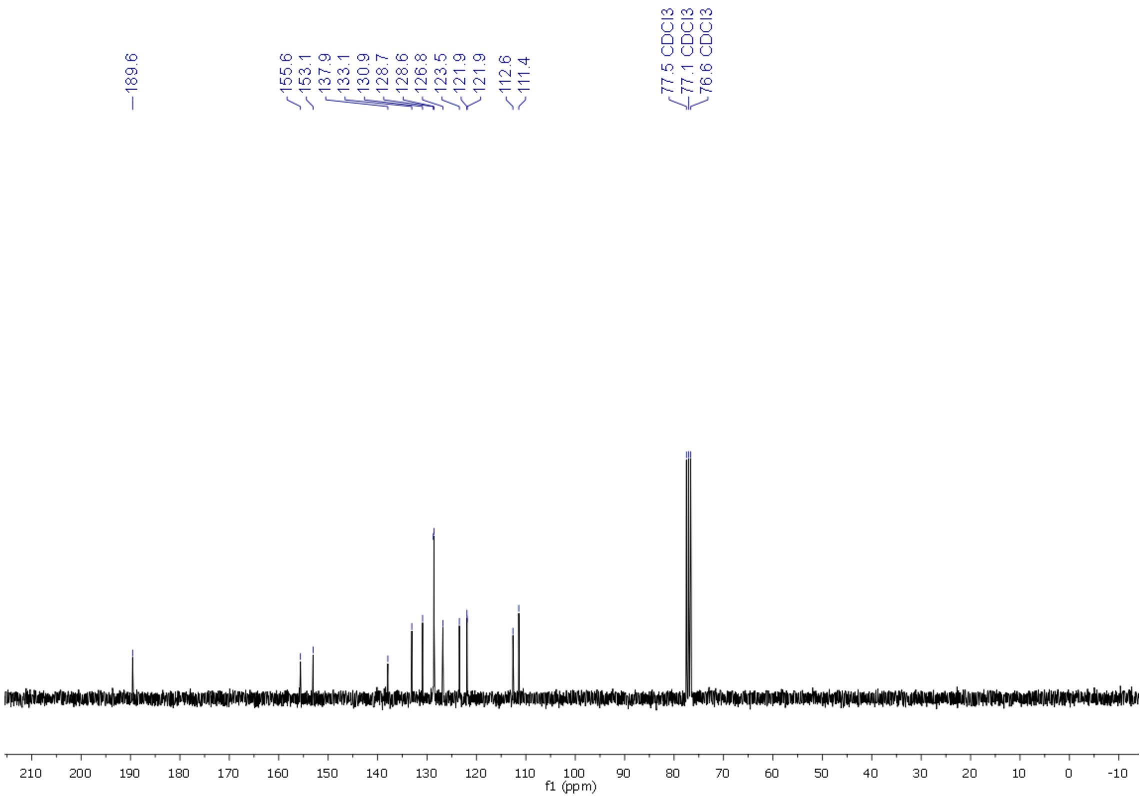

Figure S45. ^{13}C NMR (75 MHz, $CDCl_3$, ppm) of (*E*)-3-(benzofuran-2-yl)-1-phenylprop-2-en-1-one (5c).

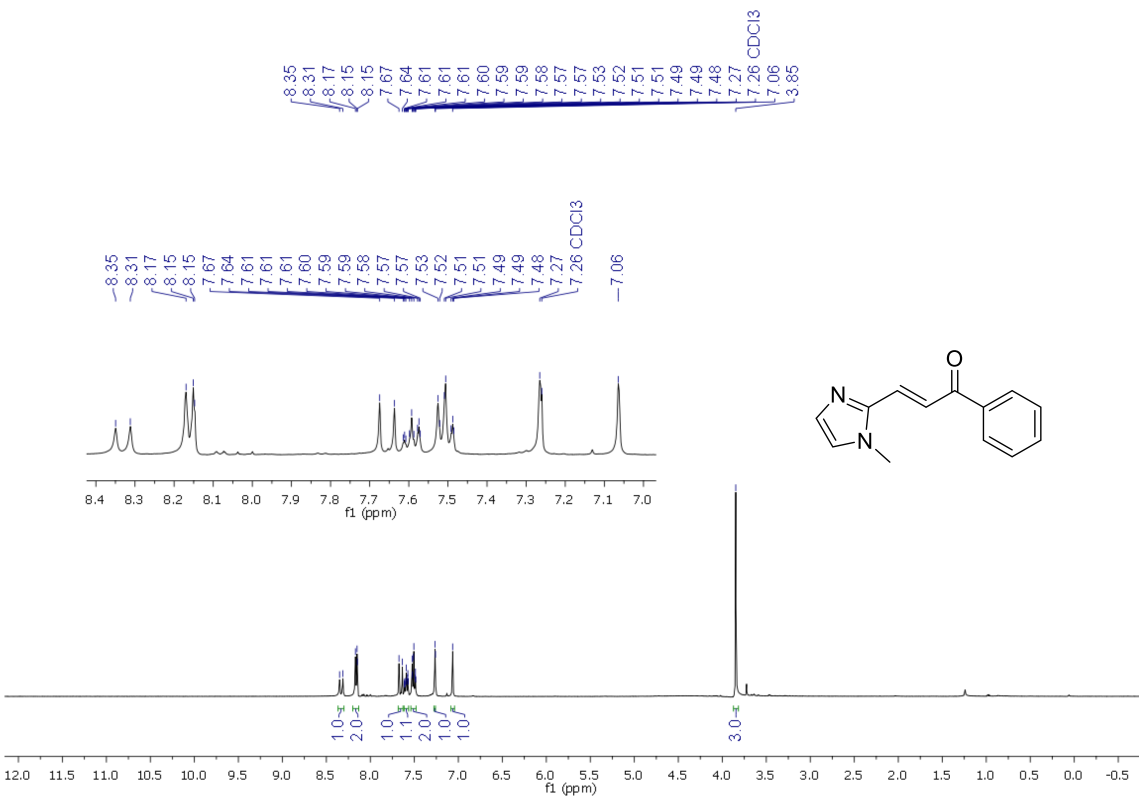

Figure S46. ^{1}H NMR (400 MHz, $CDCl_3$, ppm) of (*E*)-3-(1-methyl-1H-imidazol-2-yl)-1-phenylprop-2-en-1-one (5d).

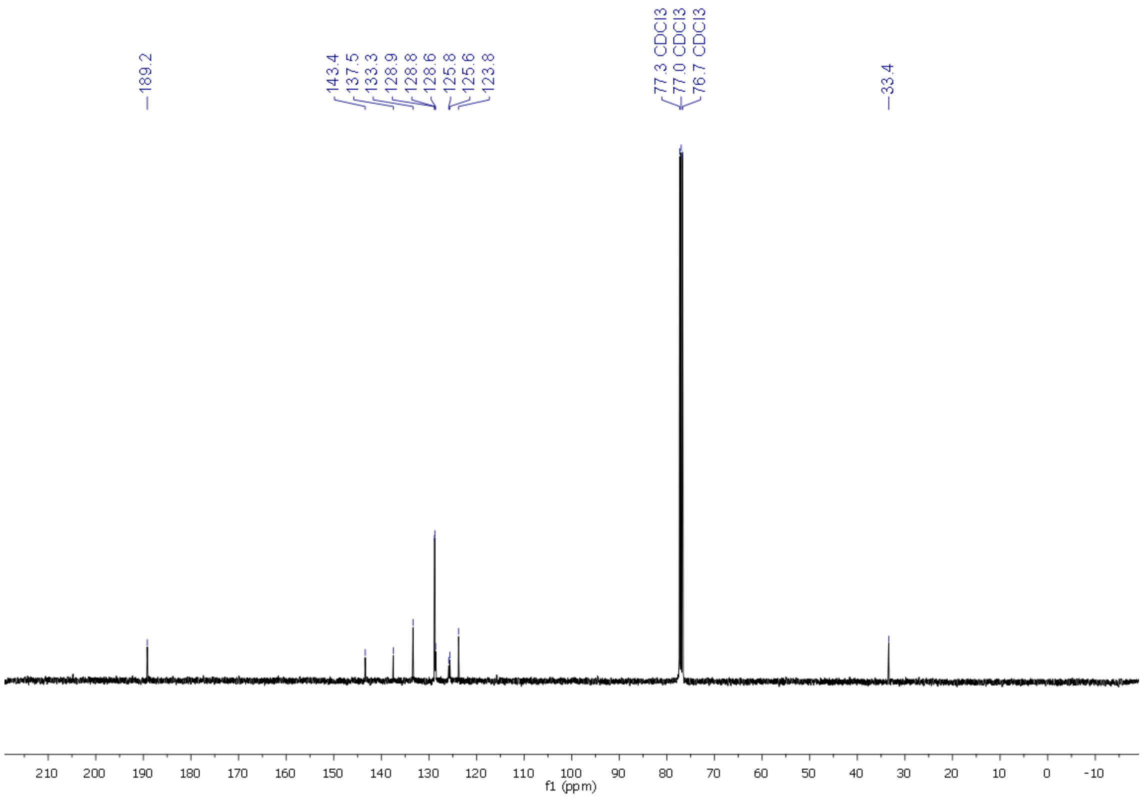

Figure S47. ^{13}C NMR (101 MHz, $CDCl_3$, ppm) of (*E*)-3-(1-methyl-1H-imidazol-2-yl)-1-phenylprop-2-en-1-one (5d).

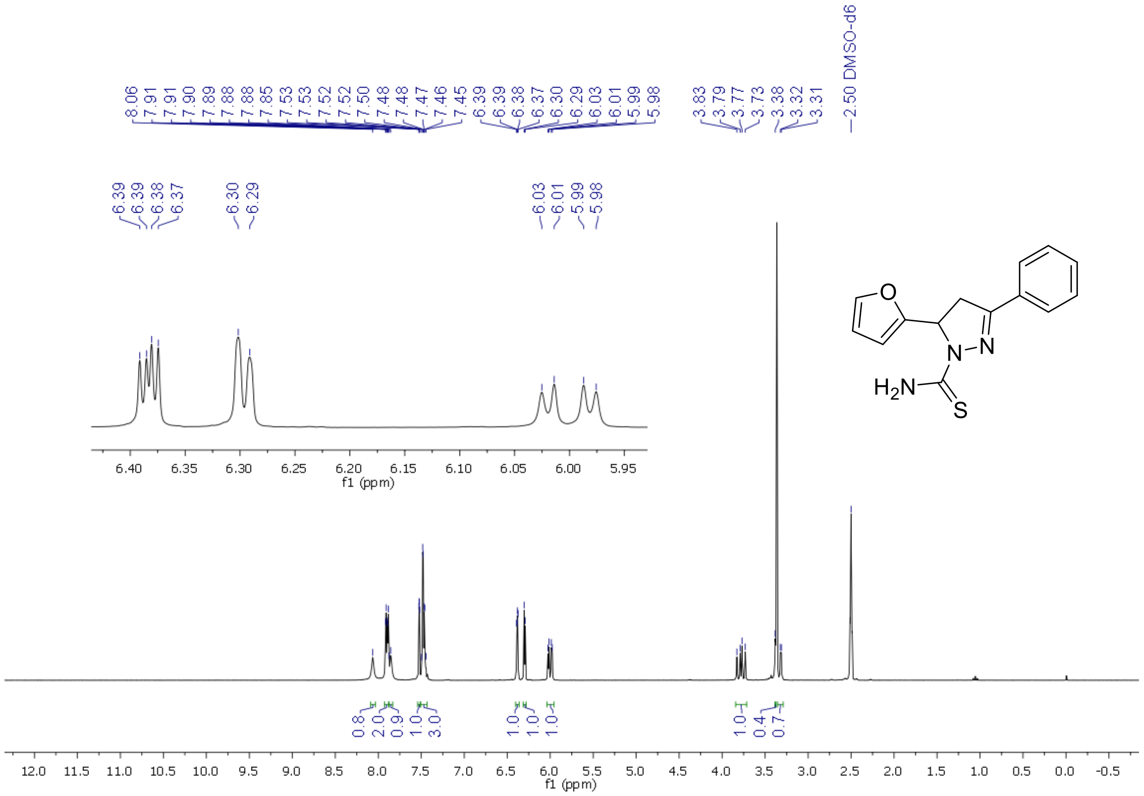

Figure S48. ^{1}H NMR (300 MHz, DMSO-d_6, ppm) of 5-(furan-2-yl)-3-phenyl-4,5-dihydro-1H-pyrazole-1-carbothioamide (6a).

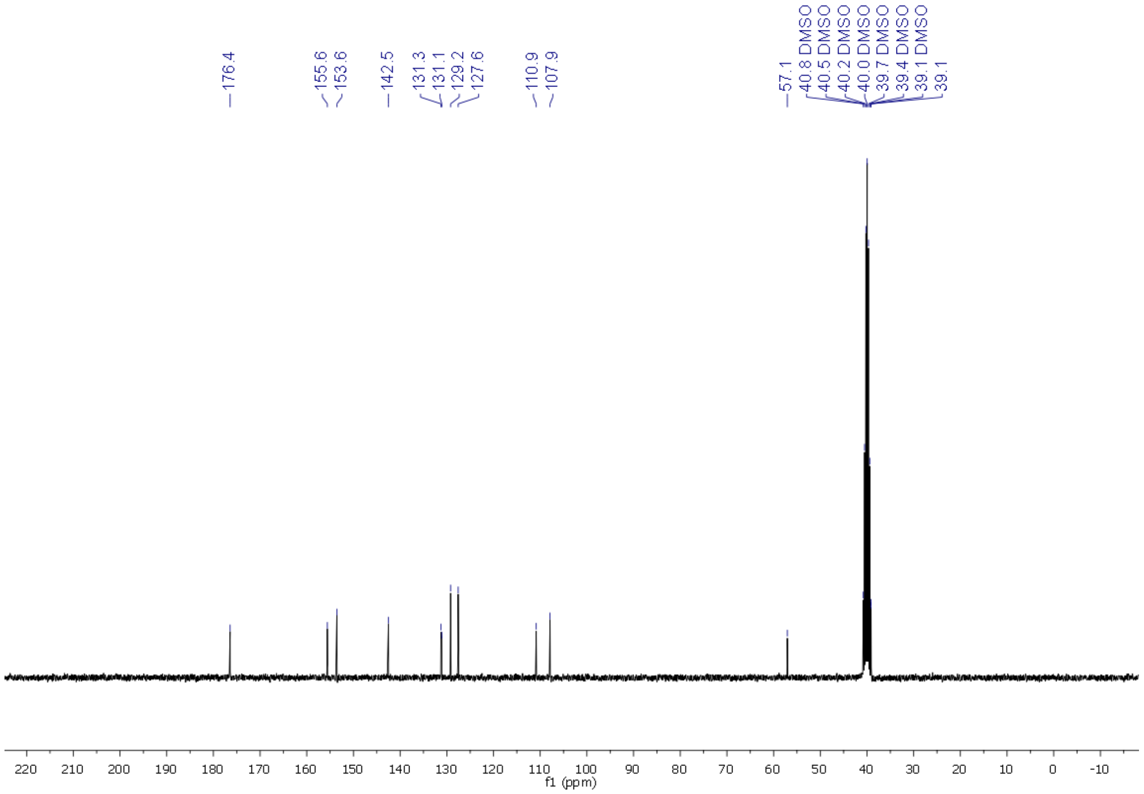

Figure S49. ^{13}C NMR (75 MHz, DMSO-d_6, ppm) of 5-(furan-2-yl)-3-phenyl-4,5-dihydro-1H-pyrazole-1-carbothioamide (6a).

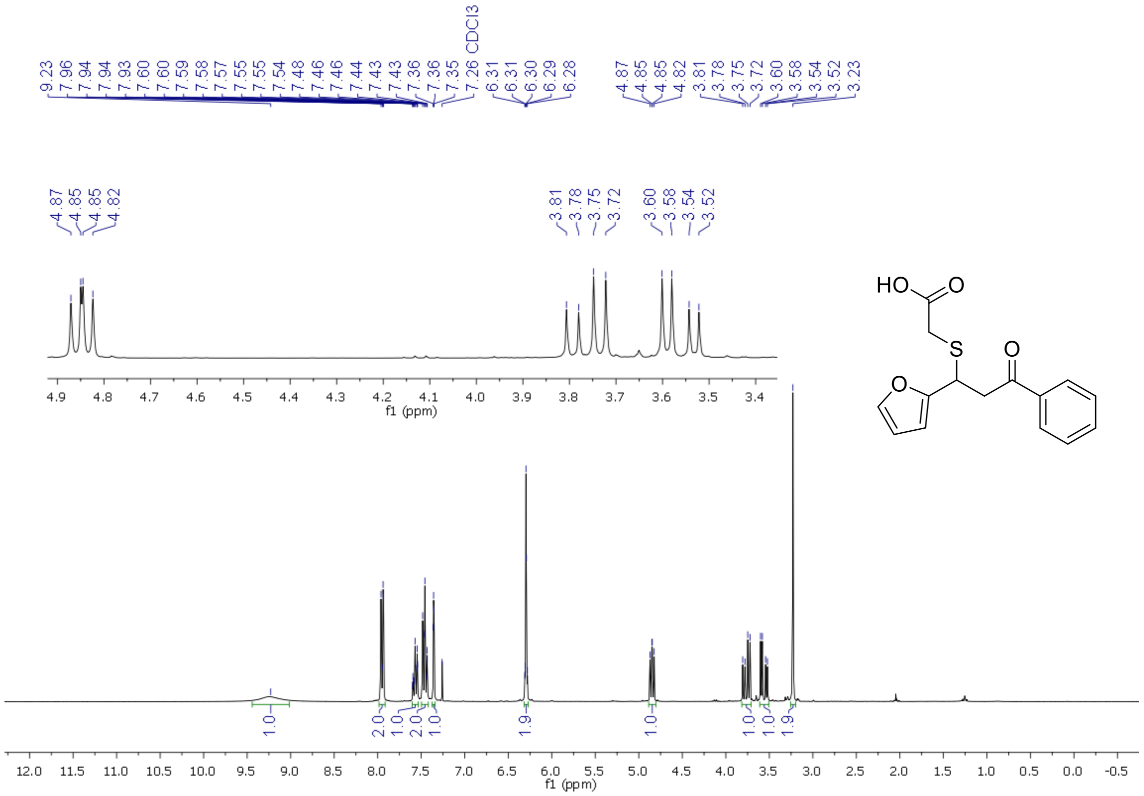

Figure S50. ^{1}H NMR (300 MHz, $CDCl_3$, ppm) of 2-((1-(furan-2-yl)-3-oxo-3-phenylpropyl)thio)acetic acid (6b).

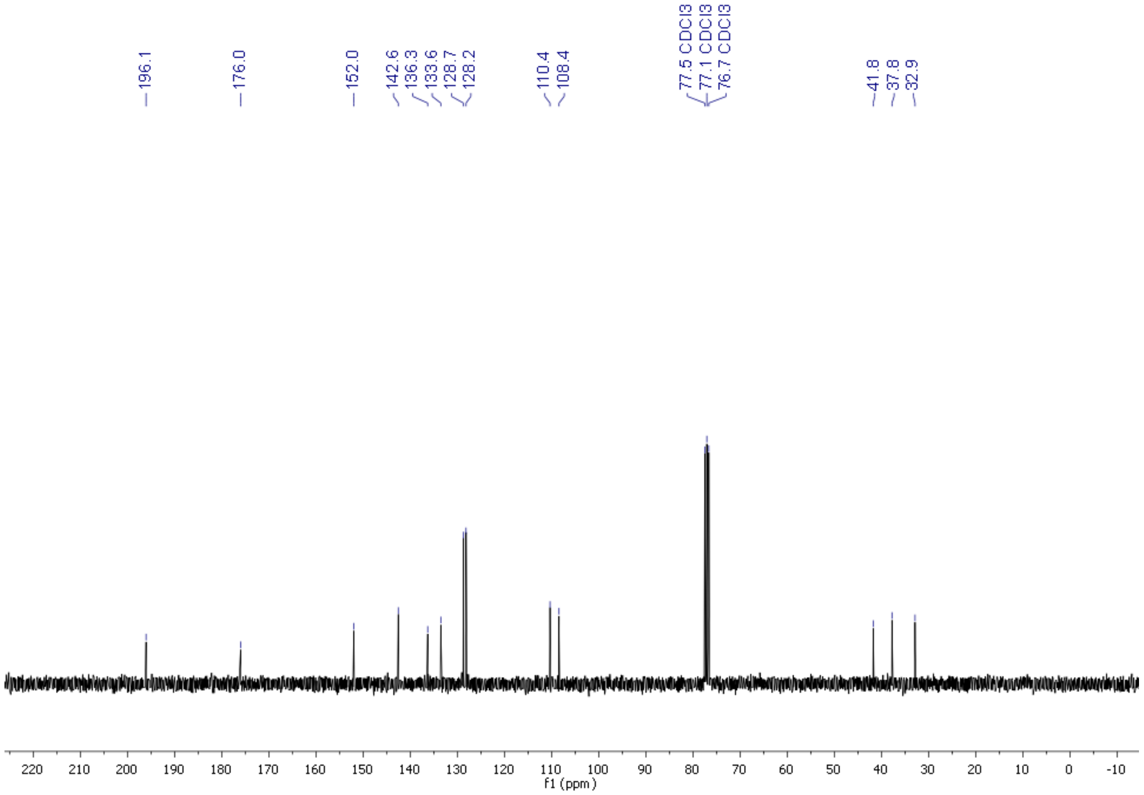

Figure S51. ^{13}C NMR (75 MHz, $CDCl_3$, ppm) of 2-((1-(furan-2-yl)-3-oxo-3-phenylpropyl)thio)acetic acid (6b).

6. References

(1) Armarego, W. L. F.; Perrin, D. D. *Purification of Laboratory Chemicals*, 4. ed., reprint.; Butterworth-Heinemann: Oxford, U. K., 2002.

(2) Silva, T. L.; Toffano, L.; Fernandes, J. B.; das Graças Fernandes da Silva, M. F.; de Sousa, L. R. F.; Vieira, P. C. Mycotoxins from Fusarium Proliferatum: New Inhibitors of Papain-like Cysteine Proteases. *Braz. J. Microbiol.* **2020**, *51* (3), 1169–1175. https://doi.org/10.1007/s42770-020-00256-7.

(3) Musumarra, G.; Ballistreri, F. P. Studies of Substituent Effects by Carbon-13 NMR Spectroscopy. Thiophene and Furan Chalcone Analogues. *Org. Magn. Reson.* **1980**, *14* (5), 384–391. https://doi.org/10.1002/mrc.1270140512.

(4) Koldobskii, A. B.; Tsvetkov, N. P.; Kalinin, V. N. Universal Method for the Functionalization of β-Bromovinyl Trifluoromethyl Ketones of Cyclobutene Series. *Dokl. Chem.* **2010**, *432* (1), 133–135. https://doi.org/10.1134/s0012500810050046.

(5) Matsuo, K.; Okumura, R.; Hayashi, H.; Aratani, N.; Jinnai, S.; Ie, Y.; Saeki, A.; Yamada, H. Phosphaacene as a Structural Analogue of Thienoacenes for Organic Semiconductors. *Chem. Commun.* **2022**, *58* (98), 13576–13579. https://doi.org/10.1039/d2cc05122b.

(6) Senaweera, S.; Weaver, J. D. S_NAr Catalysis Enhanced by an Aromatic Donor–Acceptor Interaction; Facile Access to Chlorinated Polyfluoroarenes. *Chem. Commun.* **2017**, *53* (54), 7545–7548. https://doi.org/10.1039/c7cc03996d.

(7) Wu, L.; Zhong, W.; Xu, B.; Wei, Z.; Liu, X. Synthesis and Characterization of Copper(II) Complexes with Multidentate Ligands as Catalysts for the Direct Hydroxylation of Benzene to Phenol. *Dalton Trans.* **2015**, *44* (17), 8013–8020. https://doi.org/10.1039/c5dt00575b.

(8) Mishra, A.; Rai, P.; Pandey, Y. K.; Singh, J.; Singh, J. An Eco-Sustainable Synthetic Approach for 4,5-Dihydro-1*H*-pyrazoles via DBU Catalysis in Micellar Medium. *ChemistrySelect* **2017**, *2* (34), 10979–10983. https://doi.org/10.1002/slct.201702400.

(9) Chimenti, F.; Carradori, S.; Secci, D.; Bolasco, A.; Bizzarri, B.; Chimenti, P.; Granese, A.; Yáñez, M.; Orallo, F. Synthesis and Inhibitory Activity against Human Monoamine Oxidase of N1-Thiocarbamoyl-3,5-Di(Hetero)Aryl-4,5-Dihydro-(1 H)-Pyrazole Derivatives. *Eur. J. Med. Chem.* **2010**, *45* (2), 800–804. https://doi.org/10.1016/j.ejmech.2009.11.003.

(10) Ceylan, M.; Gürdere, M. B.; Gezegen, H.; Budak, Y. Potassium–Tertiary Butoxide–Assisted Addition of Thioglicolic Acid to Chalcone Derivatives Under Solvent-Free Conditions. *Synth. Commun.* **2010**, *40* (17), 2598–2606. https://doi.org/10.1080/00397910903291129.
